# Nucleoside‐Based Hydrogel Platform Synergizes with Photothermal Effects for Enhanced Biofilm Eradication Against Periodontitis

**DOI:** 10.1002/advs.202522853

**Published:** 2025-12-20

**Authors:** Yinghui Wen, Yunfei Li, Tiannan Liu, Chongkui Sun, Hang Zhao, Yao Yuan, Shiping Yang, Tingxing Zhao, Jiang Liu

**Affiliations:** ^1^ State Key Laboratory of Oral Diseases & National Center for Stomatology & National Clinical Research Center for Oral Diseases & Research Unit of Oral Carcinogenesis and Management & Chinese Academy of Medical Sciences West China Hospital of Stomatology Sichuan University Chengdu Sichuan P. R. China; ^2^ School of Materials and Chemistry Southwest University of Science and Technology Mianyang Sichuan P. R. China; ^3^ Institute of Biomedical Engineering College of Medicine Southwest Jiaotong University Chengdu Sichuan P. R. China; ^4^ Department of Geriatrics Sichuan Provincial People's Hospital University of Electronic Science and Technology Chengdu Sichuan P. R. China

**Keywords:** antibacterial, antibiofilm, nucleoside‐based hydrogel, periodontitis, photothermal therapy

## Abstract

Periodontitis is a chronic inflammatory disease primarily driven by pathogenic biofilms, and affects more than 90% of the global population. The increasing prevalence of bacterial resistance, coupled with the protective nature of resilient biofilms, makes it challenging to achieve satisfactory therapeutic outcomes. In this study, an organic small molecule‐based photothermal reagent (**FNP**) with excellent photothermal property is designed by rational tailor of donor‐acceptor combinations, and a supramolecular nucleoside hydrogel (**ZBAg**) is developed via silver ion‐stabilized base pairing and dynamic boronate ester bonds. The **ZBAg** hydrogel exhibits a unique coordination mechanism distinct from the traditional intermolecular *i*‐motif coordination mode. The **ZBAg@FNP** hydrogel is prepared by encapsulating **FNP** within **ZBAg** hydrogel, which demonstrates excellent biocompatibility and achieves controlled Ag^+^ release triggered by localized hyperthermia. The **ZBAg@FNP** hydrogel can damage biofilm structure through photothermal therapy and then improves the penetration of Ag^+^ into the biofilms, resulting in synergistic eradication of the biofilms of oral pathogenic bacteria (*Porphyromonas gingivalis* and *Streptococcus mutans*). **ZBAg@FNP** hydrogel treatment significantly reduces the levels of proinflammatory cytokines, increases the levels of anti‐inflammatory cytokines, and reduces alveolar resorption in periodontitis of rats. This study provides a new strategy for treating periodontitis, and offers insights into the design of antibiofilm materials.

## Graphical abstract

A synergistic antibiofilm hydrogel platform (**ZBAg@FNP**) is developed by encapsulating an organic small‐molecule photothermal agent (**FNP**) within a silver ion‐mediated supramolecular hydrogel (**ZBAg**), enabling photothermal disruption of biofilm integrity and controlled Ag⁺ ion release upon irradiation. This sequential strategy achieves synergistic biofilm eradication and demonstrates therapeutic efficacy against periodontal disease.

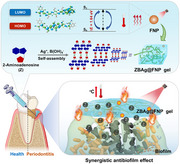

## 1 Introduction

Periodontitis, recognized by the World Health Organization as one of the three major oral diseases, is the sixth most prevalent chronic disease in the world, and affects 90% of the global population [1, 2]. Its clinical manifestations include chronic inflammation of periodontal tissues, which leads to the destruction of supporting structures such as gums, periodontal ligaments, and alveolar bone. It can even result in tooth mobility and tooth loss [3, 4]. This not only severely affects the patient's ability to chew, speak, and maintain an aesthetic appearance, but is also closely associated with the development of various systemic diseases, such as diabetes, cardiovascular diseases, and Alzheimer's disease [5, 6, 7, 8]. Periodontitis is primarily caused by bacteria and other pathogenic microorganisms that adhere to the tooth surface, and form dental plaque biofilms [9]. The main components of biofilms are extracellular polymeric substances (EPS), including polysaccharides, proteins, and extracellular DNA (eDNA), which are secreted by bacteria [10, 11]. The EPS matrix not only shields embedded bacteria and facilitates the release of pro‐inflammatory toxins but also promotes further bacterial adhesion. Eventually, they become a source of persistent infection, leading to recurrent infections in the periodontal tissues [12, 13]. As surgical treatment is invasive, leads easily to infections, and cannot ensure its therapeutic effect in advance, non‐surgical treatment forms the primary strategy for managing periodontitis. However, owing to the protective and adhesive properties of biofilms, conventional scaling and root planing methods often fail to adequately eliminate dental plaque biofilms [14, 15]. Conventional antimicrobial agents face limitations due to the protective barrier of EPS, making it difficult for them to effectively penetrate and diffuse within the biofilms [15, 16]. As a result, their efficiency in degrading oral biofilms and eradicating embedded microorganisms is low. Prolonged use of antibiotics may lead to the development of antimicrobial resistance, further compromising treatment outcomes. Therefore, although the available methods for treating periodontitis can control inflammation and alleviate symptoms to some extent, they face challenges such as the difficulty in completely removing dental plaque biofilms, high recurrence rates, and antimicrobial resistance. Hence, new therapeutic approaches or antimicrobial agents that can effectively eliminate biofilms, control infection, and reduce inflammation are needed.

Photothermal therapy (PTT), a light‐based treatment approach, uses light to activate photothermal agents that convert light energy into heat [17, 18]. This process can disrupt the structure of biofilms, which is characterized by minimal invasiveness, and a low risk of resistance [19]. However, owing to the high‐temperature requirements for effective antimicrobial treatment, excessively high temperatures may cause tissue damage, which limits the efficacy of PTT in antimicrobial applications. Silver ions (Ag^+^) are classic antimicrobial agents with several advantages, such as ease of preparation, excellent antimicrobial properties, broad‐spectrum effectiveness, and low susceptibility to resistance [20, 21]. However, the application of conventional solutions or simple physical encapsulation leads to an initial burst release and the loss of active ingredients, moreover, high concentrations of Ag^+^ released may exhibit cytotoxicity. Nucleosides, which are the basic components of DNA/RNA, possess excellent biocompatibility and biodegradability. Owing to the unique hydrogen bond donor and acceptor sites in their molecular structure, nucleosides can not only interact with Ag^+^ but also self‐assemble into supramolecular hydrogels [22, 23]. They can facilitate the controlled release of Ag^+^, improve their biocompatibility, and serve as carriers for photothermal agents. Through the photothermal effect, they disrupt biofilms and synergistically promote the penetration of Ag^+^ for antimicrobial action, enabling effective PTT and chemical antimicrobial synergistic therapy for periodontitis.

**SCHEME 1:**
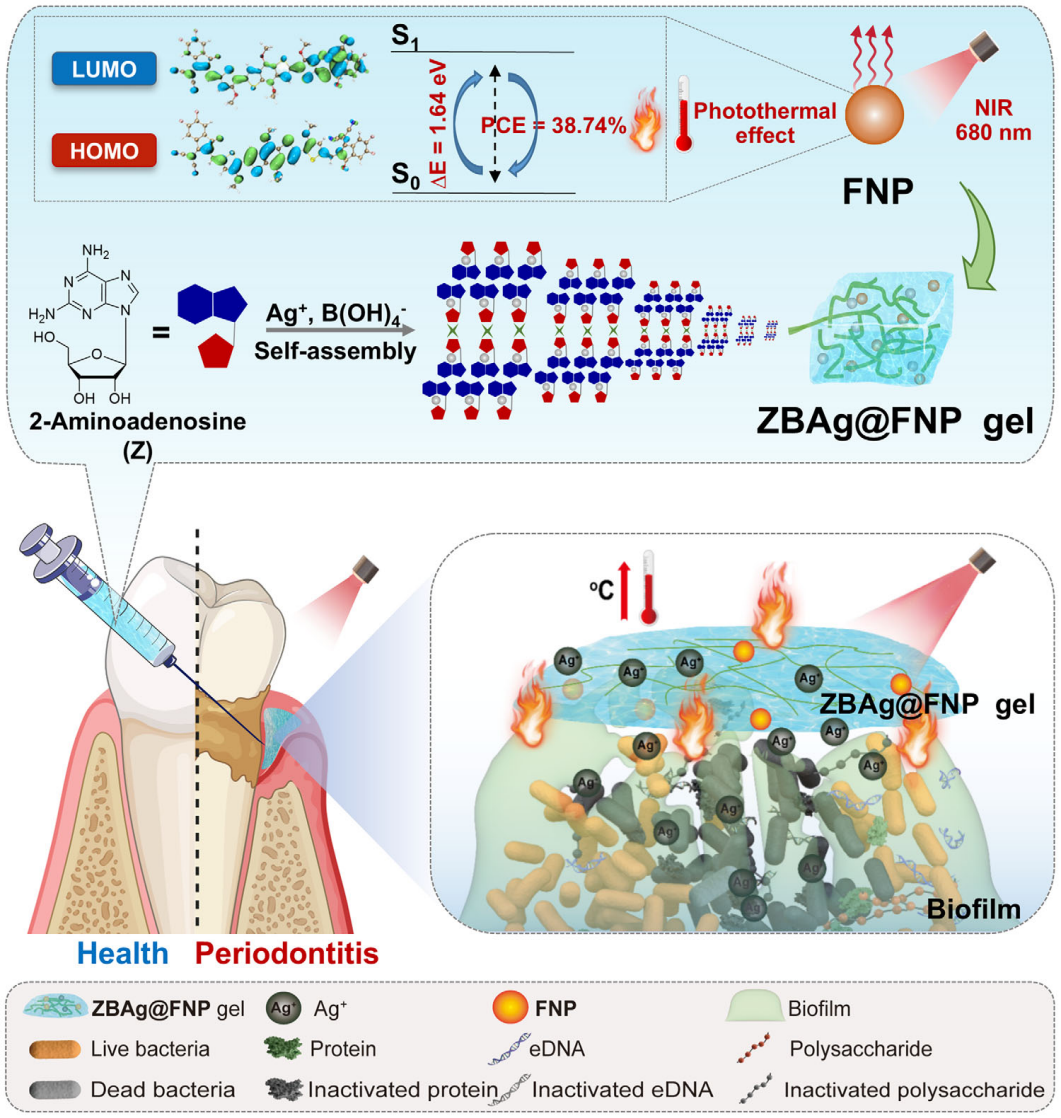
Schematic illustration of the synergistic antibiofilm effects mechanisms of **ZBAg@FNP** hydrogel on the periodontitis: **ZBAg@FNP** hydrogel inactivates EPS components (including proteins and eDNA) through localized hyperthermia to disrupt biofilm architecture, leading to enhance the permeability of Ag^+^ into biofilms. In a periodontitis rat model, this combined action of PTT and controlled Ag^+^ release from **ZBAg@FNP** hydrogel synergistically enhanced the antibiofilm efficacy, significantly suppressed inflammation, promoted alveolar bone regeneration, and restored tissue integrity, thereby demonstrating promising therapeutic efficacy against periodontal disease.

Compared to metal nanoparticles and polymer‐type photothermal agents, organic small molecule‐based photothermal agents exhibit tunable photophysical properties, definite structures, and better biodegradation properties in vivo [24, 25]. In this study, we designed organic small molecule‐based photothermal agents (**BF** and **BCl**) with near‐infrared (NIR) absorption through the rational design of donor‐acceptor combinations. **BF** and **BCl** were encapsulated by Pluronic F127 to prepare photothermal nanoparticles (**FNP** and **ClNP**), respectively, and **FNP** exhibited higher photothermal conversion efficiency (PCE) than **ClNP**. We found that 2‐aminoadenosine (**Z**), a nucleoside analog with dual NH_2_ groups and multiple hydrogen‐bonding motifs, could self‐assemble into a supramolecular hydrogel (**ZBAg**) via silver ion‐stabilized base pairing and dynamic boronate ester bonds. **FNP** was encapsulated within the **ZBAg** hydrogel to form the **ZBAg@FNP** hydrogel, which exhibited good mechanical properties, photothermal effect, and biocompatibility. The findings showed that the **ZBAg@FNP** hydrogel could inactivate major EPS components such as polysaccharides, proteins, and eDNA through the photothermal effect generated by **FNP**, disrupting the structures of the biofilms and promoting the diffusion and penetration of Ag^+^ into biofilms, resulting in strong antimicrobial activity and biofilm eradication effects against oral pathogenic bacteria, such as *Porphyromonas gingivalis* (*P. gingivalis*) and *Streptococcus mutans* (*S. mutans*). Moreover, the **ZBAg@FNP** hydrogel effectively inhibits the inflammatory response, by reducing the levels of proinflammatory cytokines and increasing the levels of anti‐inflammatory cytokines. It also promotes alveolar bone regeneration, and promotes tissue repair in a periodontitis rat model, demonstrating that its the spotential as a promising material for biofilm eradication in periodontitis (Scheme 1).

## 2 Results and Discussion

### 2.1 Design, Synthesis, and Characterization of Photothermal Agents

**FIGURE 1:**
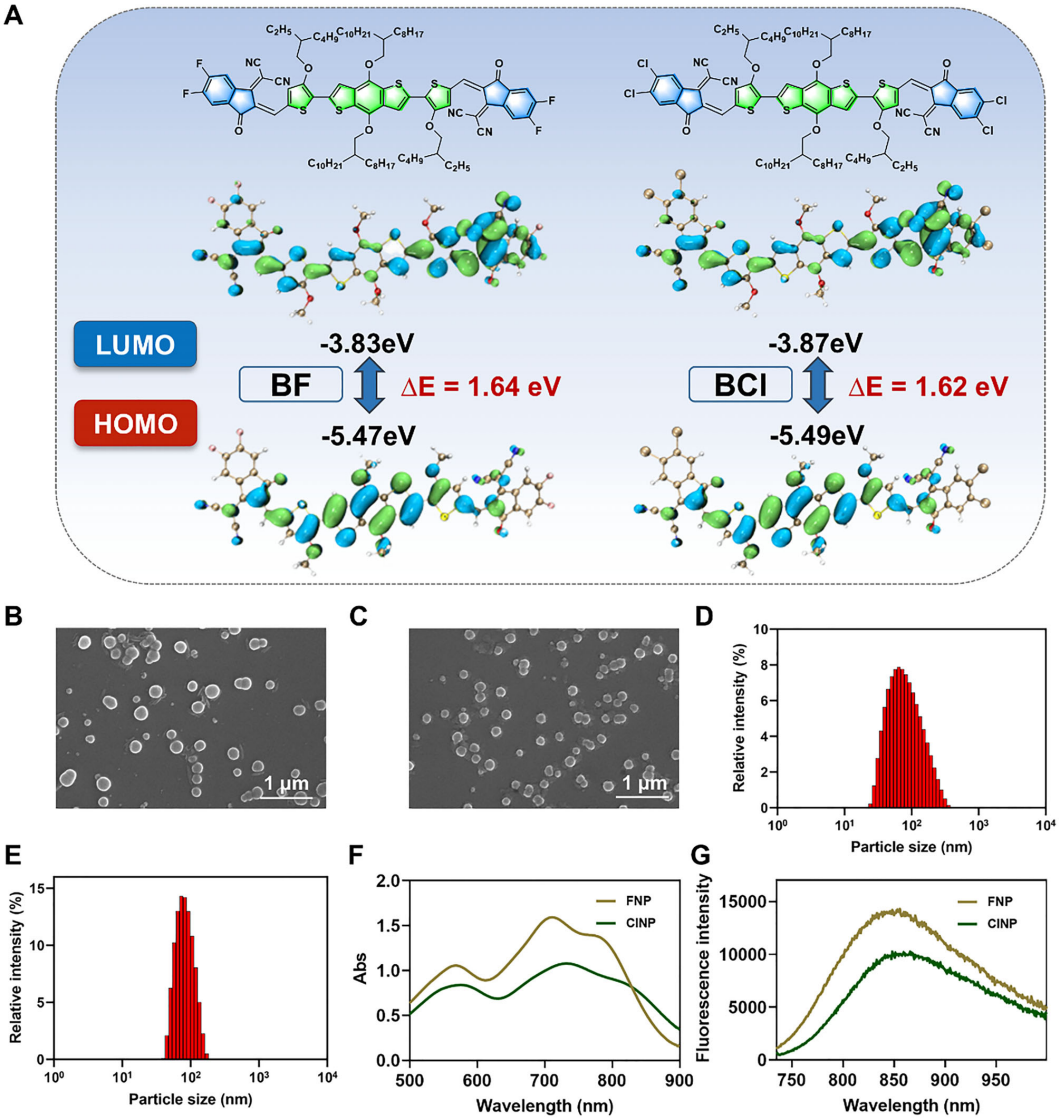
Characterization of photothermal agents. (A) The HOMO‐LUMO distributions of **BF** and **BCl**. (B) SEM image of **FNP** (scale bar: 1 µm). (C) SEM image of **ClNP** (scale bar: 1 µm). (D) Dynamic light scattering detection of **FNP**. (E) Dynamic light scattering detection of **ClNP**. (F) UV–vis absorption spectrum of **FNP** and **ClNP** at the concentrations of 20 µg mL^−1^. (G) Fluorescence spectrum of **FNP** and **ClNP** at the concentrations of 20 µg mL^−1^.

In general, the preparation process of metal/polymer nanoparticles is more complex, and their corresponding biodegradability remains a subject of debate [26, 27]. Recently, acceptor‐donor‐acceptor (A–D–A) type small molecules have received significant attention in PTT because of their diverse molecular structures and excellent photophysical properties. These molecules, through rational manipulation of electron donor/acceptor moieties, can achieve efficient photothermal conversion, and demonstrate remarkable potential in biomedical applications, including oncotherapy and antibacterial treatment [28, 29]. Initially, two small molecules (**BF** and **BCl)** were synthesized via Stille coupling and Knoevenagel condensation reactions (the detailed synthetic procedures are provided in Figures S1–S10). Then, density functional theory (DFT) calculations (Figure 1A) were employed to reveal their electronic characteristics. The HOMO/LUMO energy levels were –5.47/–3.83 eV for **BF** and –5.49/–3.87 eV for **BCl**, respectively. The LUMO orbitals were predominantly localized at the peripheral acceptor units, whereas the HOMO distributions extended across the central electron‐donating moiety and thiophene bridges. Besides, **BCl** exhibited a slightly reduced bandgap (ΔE) compared to **BF**, suggesting its stronger D–A interaction.

Subsequently, the UV–vis–NIR absorption and fluorescence spectra were measured. Both compounds of **BF** and **BCl** exhibited absorption bands in the 500–900 nm range, with **BF** showing a maximum absorption at 701 nm, while **BCl** exhibited a 20 nm redshift to 721 nm, which may be attributed to its narrower bandgap (Figure S11A). Interestingly, **BF** demonstrated superior molar extinction coefficient compared to **BCl**, indicating its enhanced light‐harvesting capability. Upon excitation at 680 nm, fluorescence emission peaks were observed at 810 nm for **BF** and 795 nm for **BCl**. The higher absorbance and larger Stokes shift of **BF** suggest a greater total excited‐state energy and enhanced non‐radiative energy dissipation, which may favor the photothermal conversion process (Figure S11B).

To address the inherent hydrophobicity of **BF** and **BCl**, we encapsulated them into nanoparticles (**FNP** and **ClNP**) using the biocompatible amphiphilic molecule of Pluronic F127 [30, 31]. Scanning electron microscopy (SEM) examinations confirmed monodisperse spherical morphologies with hydrodynamic diameters of 56.5 nm (**FNP**) and 67.5 nm (**ClNP**) (Figure 1B–E). **FNP** exhibited a red‐shifted absorption peak at 711 nm, which is 10 nm longer than that of **BF** in the solution state, along with an emission peak at 854 nm. Additionally, **ClNP** showed absorption and emission maxima at 734 and 866 nm, respectively (Figure 1F,G).

### 2.2 Preparation and Self‐Assembly Mechanism of the ZBAg Hydrogel

**FIGURE 2:**
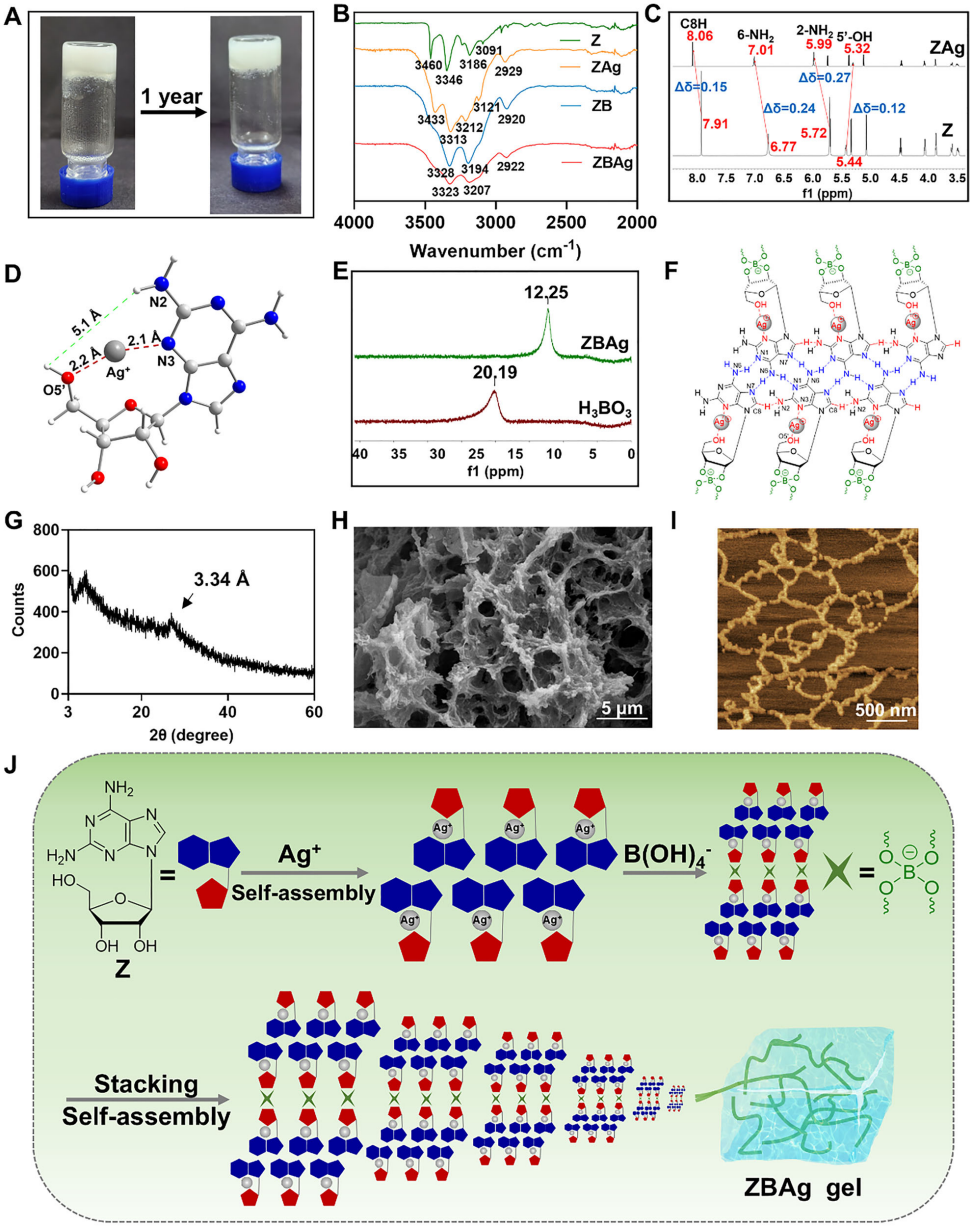
Preparation and characterization of hydrogel. (A) Photograph of **ZBAg** hydrogel. (B) FTIR spectra of **Z**, **ZB**, **ZAg** and **ZBAg** hydrogels. (C) ^1^H NMR spectra of **Z** and **ZAg** in DMSO‐*d_6_*. (D) The optimized conformations of **ZAg**. Atoms are coded as followed: red, oxygen; blue, nitrogen; white, hydrogen; light gray, carbon; dark gray, silver ion. (E) ^11^B NMR spectra of H_3_BO_3_ and **ZBAg** hydrogel. (F) The base pairing and Ag^+^ coordination sites of **ZBAg** hydrogel. (G) PXRD of **ZBAg** hydrogel. (H) SEM image of **ZBAg** hydrogel (scale bar: 5 µm). (I) AFM image of **ZBAg** hydrogel (scale bar: 500 nm). (J) Schematic illustration of the formation of **ZBAg** hydrogel.

Nucleoside‐based supramolecular hydrogels have received attention in biomedicine because of their manifold noncovalent interactions, biocompatibility, and multifunctional responsiveness [32]. Our research group designed a series of nucleoside‐based hydrogel systems demonstrating therapeutic potential for biomedical applications [33, 34, 35, 36, 37, 38]. Compound **Z**, a nucleoside analogue featuring dual NH_2_ groups, exhibits enhanced hydrogen‐bonding capacity due to its multivalent configuration. This facilitates the formation of extended hydrogen‐bonding networks, enabling superior self‐assembly compared to adenosine [39]. We found that it can self‐assemble into a supramolecular hydrogel (**ZAg**) in the presence of Ag^+^, but the system has limited stability (disintegrating within ∼5 min, Figure S12). This likely occurs because the flexible, non‐planar sugar ring and freely rotating glycosidic bond of **Z** induce different geometric conformations that exist in dynamic equilibrium, and water molecules can directly or indirectly affect the stability of the geometric conformation of nucleoside sugar ring, which also compromises the stability of the hydrogel [35]. Interestingly, we noticed that the sugar ring of **Z** contains a vicinal diol structure, which spontaneously forms dynamic covalent bonds with boronic acid under alkaline conditions [33, 40]. After introducing dynamic boronate ester bonds, the involvement of the 2'‐OH and 3'‐OH groups of the nucleoside sugar ring in the reaction can partially reduce the effect of hydration on molecular self‐assembly. Additionally, owing to the structural constraints after the reaction, the geometric conformation of the sugar ring can be stabilized. Furthermore, a single boronic acid moiety can form dynamic boronate ester bonds with two **Z** molecules, increasing intermolecular connectivity and further enhancing the self‐assembly properties of the nucleoside. Leveraging these interactions, we successfully constructed a silver‐mediated nucleoside hydrogel (**ZBAg**) with excellent stability through the incorporation of boronate ester bonds (Figure 2A).

To investigate the coordination of **Z** and Ag^+^, electrospray ionization mass spectrometry (ESI‐MS), matrix‐assisted laser desorption/ionization time‐of‐flight (MALDI‐TOF) MS, and Fourier transform infrared spectroscopy (FTIR) experiments were performed. ESI‐MS (Figure S13) and MALDI‐TOF MS (Figure S14) spectra show a peak at 390.86 and 391.0455 in **ZBAg** hydrogel, respectively, providing evidence for the complex of one **Z** coordinating one Ag^+^. As shown in Figure 2B, characteristic absorptions in the 3000–3400 cm^−1^ region for **Z** were attributed to N‐H/O‐H stretching vibrations. Upon Ag^+^ coordination (**ZAg**/**ZBAg**), the peaks assigned to OH and NH_2_ exhibited significant broadening and shifting, which indicated the amine group and hydroxy group in **ZAg**/**ZBAg** participate in complexation [41]. To further elucidate the interaction sites and binding mode, we simplified the system and conducted NMR measurements on **Z** and **ZAg**. The structural assignment of **Z** was characterized by 1D (^1^H and ^13^C) nuclear magnetic resonance (NMR) and 2D NMR (COSY, HSQC and HMBC) spectra (Figures S15–S19). The ^1^H NMR spectra recorded at different ratio of **Z**/Ag^+^ are consistent with the MS results, confirming the binding ratio of 1:1 between **Z** and Ag^+^ (Figure S20). Meanwhile, noticeable chemical shift changes were observed for the ^1^H NMR signals of C8H, 6‐NH_2_, 2‐NH_2_ and 5'‐OH (Figure 2C; Figure S20). Particularly, the signal peaks of C8H, 6‐NH_2_, and 2‐NH_2_ shifted downfield, while that of 5'‐OH shifted upfield. Our previous work has demonstrated that the base pairing in **Z** crystal primarily relies on intermolecular hydrogen bonds formed between 6‐NH_2_ and N1/N7 [42]. Additionally, 5'‐OH forms an intramolecular hydrogen bond with N3 to stabilize the sugar ring conformation, and participates in intermolecular hydrogen bonds with neighboring 2'‐OH and 3'‐OH groups to link sugar rings, thereby promoting the formation of the self‐assembled structure. Because the deshielding effect caused by hydrogen bond typically leads to downfield shifts in ^1^H NMR signals (i.e., increased chemical shifts) [43, 44, 45]. Therefore, it is speculated that Ag^+^ may coordinate with 5'‐OH, weakening or even disrupting its hydrogen bond interactions. This coordination may in turn promote the formation of noncanonical hydrogen bonds between C8H and N2, and further stabilize the hydrogen bonds between 6‐NH_2_ and N1/N7 [42]. FTIR also shows the C8‐H also attending in as the stretching band at 3091 cm^−1^ (**Z**) shifted to 3121 cm^−1^ (**ZAg**) (Δν = +30 cm^−1^) [46]. To further verify this hypothesis, we preformed structure optimization and frequency calculation at the B3LYP‐D3/def2svp level using quantum chemical methods, which revealed that the introduction of Ag^+^ leads to coordination with N3 and O5' of **Z**, which induces a rotation of the sugar ring 5'‐OH group and increases the distance between O5'H and N2H (Figure 2D). For this reason, a weak NOE signal between 5'‐OH and 2‐NH_2_, along with nearly disappeared NOE signals between 5'‐OH and 2'‐OH/3'‐OH were detected in the NOESY spectrum of **ZAg**, further supports that the original hydrogen bonds involving 5'‐OH were disrupted by the coordination of Ag^+^ (Figure S21). The variable temperature ^1^H NMR (VT ^1^H NMR) also confirmed that, compared to **Z**, stronger hydrogen bonds involving C8H, 2‐NH_2_, and 6‐NH_2_ are formed in **ZAg** [47, 48]. The chemical shift for 2‐NH_2_ and 6‐NH_2_ of **ZAg** at 55°C–65°C were comparable to those of **Z** at room temperature, and C8H, 2‐NH_2_, and 6‐NH_2_ of **ZAg** exhibited more pronounced chemical shift changes from 25°C to 95°C (Figure S22), with Δ*δ* = 0.15, 0.42 and 0.49, respectively. Briefly, Ag^+^ coordinates with **Z**, disrupting the hydrogen bonding interaction of 5'‐OH, thereby promoting the formation of a noncanonical hydrogen bond (C8H—N2) and the stabilizing of base pairing (Figure S23).

Subsequently, ^11^B NMR experiment was performed to confirm the formation of dynamic borate diester bonds. The boron peak shifts to the upfield region in ^11^B NMR after the formation of esters [49, 50, 51, 52]. In the **ZBAg** hydrogel, the peak attributed to the respective boronate ester was observed at 12.25 ppm with free H_3_BO_3_ (20.19 ppm) (Figure 2E). FTIR also confirmed the formation of boronate esters. As indicated in Figure 2B, **Z** and **ZAg** showed a significant characteristic for ν(‐OH) at 3460 and 3433 cm^−1^, respectively, while no noticeable characteristics at 3400–3500 cm–^1^ were observed for the **ZB** and **ZBAg** hydrogel, indicating that the diols in **Z** participated in the formation of the **ZB** and **ZBAg** molecular structure (Figure 2F). Furthermore, powder X‐ray diffractometry (PXRD) analysis was performed. As shown in Figure 2G, **ZBAg** hydrogel revealed a large peak at 26.68° (d = 3.34 Å), which corresponded to the *π*–*π* stacking distance between aromatic rings [53]. Since Ag^+^ carries a positive charge while boronate ester bonds are negatively charged, electrostatic interactions between Ag^+^ with boronate ester bonds, together with *π*–*π* stacking, may synergistically promote the formation of self‐assembled network in **ZBAg** hydrogel. To characterize the microstructures of the **ZBAg** hydrogel, SEM, transmission electron microscope (TEM), and atomic force microscopy (AFM) were performed. Porous structures with pore diameters of about 5 µm were detected in the **ZBAg** hydrogel (Figure 2H), indicating that enough space was available to accommodate the photothermal reagents. To obtain more detailed morphological information, the **ZBAg** hydrogel consists of nanowires with sizes ranging from 10 to 50 nm (Figure 2I; Figures S24 and S25). Overall, the above research elucidates the self‐assembly mechanism of the **ZBAg** hydrogel. Unlike the traditional intermolecular *i*‐motif coordination mode [54, 55], a novel coordination mechanism has been identified, in which Ag⁺ forms an intramolecular coordination with the N3 and O5' sites of **Z**. This Ag⁺ coordination disrupts 5'‐OH's original hydrogen bonds, leading to sugar ring conformation rotation, and a C8H—N2 hydrogen bond forms while base pairing hydrogen bonds (6‐NH_2—_N1/N7) strengthen. The free 2'‐OH and 3'‐OH connect the sugar rings via dynamic borate diester bonds, resulting in the molecular crosslinked structure of **ZBAg**. Subsequently, it assembles into nanowires via *π*–*π* stacking and electrostatic interaction. Finally, these nanowires intertwine and self‐assemble into porous structures, leading to the generation of the **ZBAg** hydrogel (Figure 2J).

### 2.3 The Mechanical Property of the Hydrogel Systems

The mechanical properties of hydrogels are important parameters for biomedical applications. Rheological measurements were performed to evaluate the viscoelastic injectability and self‐healing properties of the **ZBAg** hydrogel. The storage modulus and loss modulus, denoted as G′ and G″, respectively, were used to assess the elastic properties and fluidity [40]. **ZBAg** hydrogel possesses a higher G′ compared to G″ (Figure S26A), indicating that it continuously displays solid‐like characteristics over the entire applied frequency range. Introducing dynamic covalent bonds into the hydrogel might allow the gel‐sol transition of the hydrogel [56]. The formation of dynamic borate esters in the **ZBAg** hydrogel suggests its potential for use as an injectable hydrogel for local drug delivery. The viscosity of the **ZBAg** hydrogel decreased with increasing shear rate (Figure S26B), indicative of shear‐thinning behavior resulting from the breakdown of reversible interactions within the hydrogel. The **ZBAg** hydrogel had a higher G′ than G″ before the strain amplitude reached 15%; and it shifted from a solid‐like to a fluid‐like state after 15% stain (Figure S26C).

**FIGURE 3:**
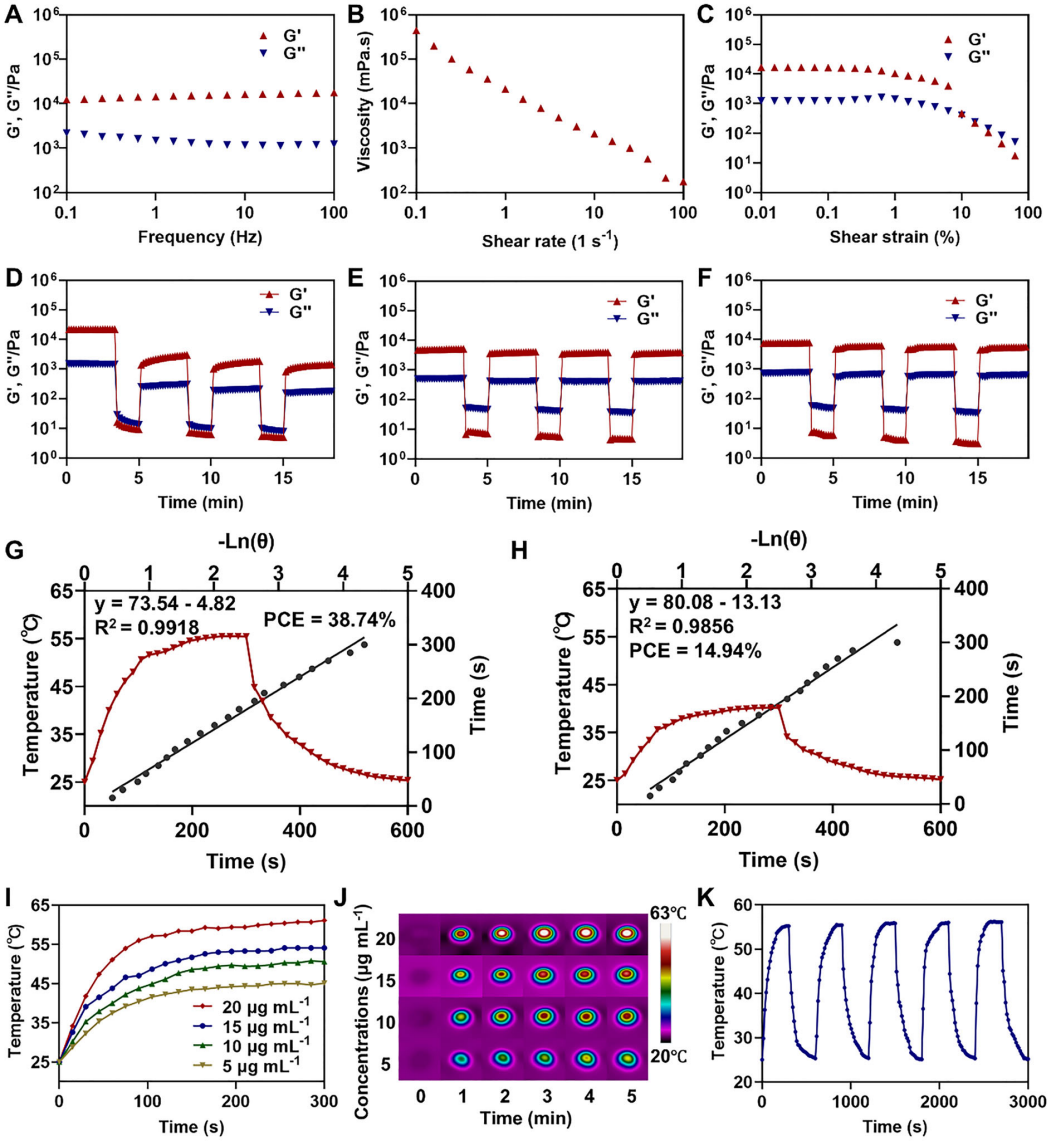
The rheological measurements and photothermal performance of hydrogel system. (A) Evolution of G’ and G″ as a function of frequency sweep for **ZBAg@FNP** hydrogel. (B) Viscosity test of **ZBAg@FNP** hydrogel. (C) Evolution of G′ and G″ as a function of strain for **ZBAg@FNP** hydrogel. (D–F) The damage‐healing property of hydrogels by continuous step strain measurements of **ZBAg** (D), **ZBAg@FNP** (E), and **ZBAg@ClNP** (F) hydrogels, respectively. (G,H) The PCE of **FNP** (G) and **ClNP** (H). (I) Temperature change curves of **ZBAg@FNP** at a series of concentrations (5, 10, 15, 20 µg mL^−1^) under 1.0 W cm^−2^ NIR irradiation. (J) Near‐infrared imaging of **ZBAg@FNP** at a series of concentrations (5, 10, 15, 20 µg mL^−1^) under 1.0 W cm^−2^ NIR irradiation. (K) Temperature elevation of **ZBAg@FNP** hydrogel (15 µg mL^−1^, 1 W cm^−2^) over five cycles of laser irradiation on/off.

Further rheological characterization of the **ZBAg** hydrogel as a carrier of **FNP** and **ClNP** was performed. After loading **FNP** and **ClNP**, the newly developed hydrogel systems were named the **ZBAg@FNP** and **ZBAg@ClNP** hydrogels, respectively. The rheological results showed that these hydrogels also exhibit solid‐like characteristics over the entire applied frequency range, and shearing‐thinning properties (Figure 3A–C; Figure S26D–F). Besides, the circle strain time sweep rheology assessments revealed that the **ZBAg** hydrogel exhibited self‐healing properties after experiencing high strain breaking (Figure 3D). These experiments confirmed that the **ZBAg** hydrogel exhibited remarkable shear‐thinning properties and the ability to rapidly revert to its gel state. Based on these findings, it can be inferred that dynamic borate ester bonds and noncovalent interactions (including hydrogen bonds, *π*–*π* stacking, coordination interactions), contribute to the stabilization of the **ZBAg** hydrogel network, resulting in its self‐healing capabilities and suitability for local administration through injection. The **ZBAg@FNP** and **ZBAg@ClNP** hydrogels displayed better self‐healing properties than the **ZBAg** hydrogel (Figure 3D–F), which may be attributed to the presence of hydrogen bonds between the hydrogel and nanoparticles. These findings indicate that encapsulating **FNP** and **ClNP** did not alter the rheological properties of the **ZBAg@FNP** and **ZBAg@ClNP** hydrogels. In fact, their self‐healing performance was enhanced, highlighting their potential for further biomedical applications.

### 2.4 Photothermal Performance of Photothermal Reagents and Hydrogel Systems

The increase in local temperature (above 50°C) generated by NIR light irradiation can be fatal through protein denaturation, structural decomposition, and hyperthermic cellular damage, ultimately killing bacteria and disrupting biofilms [57]. In this study, the photothermal effect of the loaded **FNP** and **ClNP** was considered important for the synergistic antibacterial activity of the **ZBAg@FNP** and **ZBAg@ClNP** hydrogels. To evaluate the photothermal performance of these two photothermal reagents, the temperature variations of **FNP** and **ClNP** were monitored under 680 nm laser irradiation for 5 min. The photothermal performance of photothermal agents is substantially affected by many factors, such as the molecular concentration and the irradiation parameters [58]. Clearly, **FNP** and **ClNP** exhibited temperature increases that were dependent on both concentration and laser power density, respectively (Figure S27A–F). These findings suggest that the photothermal effects of **FNP** and **ClNP** are tunable, allowing the extent of hyperthermia to be easily controlled by adjusting the irradiation conditions. Notably, at a concentration of 15 µg mL^−1^ under identical irradiation conditions, **ClNP** induced only a gradual temperature increase to 39.6°C, whereas **FNP** rapidly raised the temperature above 50°C, highlighting the superior photothermal generation capability of **FNP** (Figure S27A–D). Further research revealed that the PCE of **FNP** reached 38.74% (Figure 3G), representing a 2.6‐fold improvement compared to the 14.94% of **ClNP** (Figure 3H). These findings highlight the promising potential of **FNP** for photothermal antibacterial and antibiofilm therapies.

The photothermal effects of using the **ZBAg** hydrogel as a carrier of **FNP** and **ClNP** were investigated. The results showed that the photothermal effects of the **ZBAg@FNP** and **ZBAg@ClNP** hydrogels greatly depended on their concentrations (Figure 3I,J; Figure S28). To further evaluate the photothermal stability, the **ZBAg@FNP** and **ZBAg@ClNP** hydrogels were irradiated for five cycles (680 nm, 1.00 W cm^−2^, 5 min on and 5 min off). After five cycles of irradiation and heating‐natural cooling, the photothermal effects of the **ZBAg@FNP** and **ZBAg@ClNP** hydrogels remained relatively stable (Figure 3K; Figure S29). These findings showed that the photothermal properties of **FNP** and **ClNP** were not affected after being encapsulated into the **ZBAg@FNP** and **ZBAg@ClNP** hydrogels. As **FNP** showed better photothermal performance than **ClNP**, the **ZBAg@FNP** hydrogel was selected as a candidate for periodontitis treatment in subsequent studies.

### 2.5 Biodegradation and Biocompatibility of Hydrogel Systems

**FIGURE 4:**
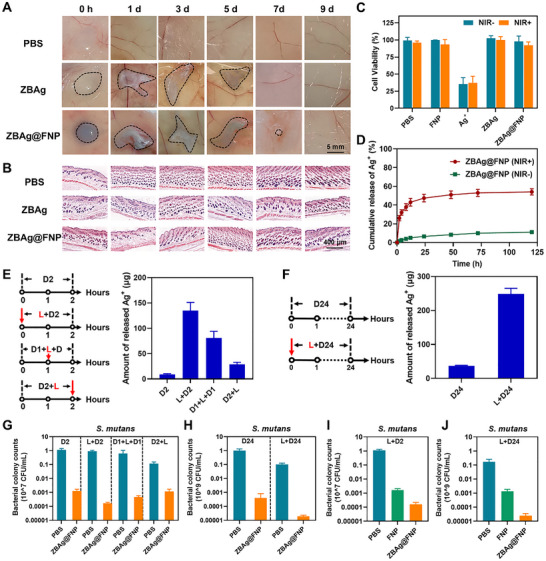
Biocompatibility and Ag^+^ release behavior of ZBAg@FNP hydrogel. (A) Images of PBS, **ZBAg** and **ZBAg@FNP** hydrogels were taken at 0, 1, 3, 5, 7, 9 d post‐injection (*n* = 5; scale bar: 5 mm). (B) H&E staining images of skin after the injection of PBS, **ZBAg** and **ZBAg@FNP** hydrogels subcutaneously (*n* = 5; scale bar: 400 µmm). (C) Cell viability of the L929 cells treated with PBS, **FNP**, Ag^+^, **ZBAg** and **ZBAg@FNP** hydrogels (*n* = 3). (D) The percentage of cumulative release of Ag⁺ relative to the initially encapsulated amount in **ZBAg@FNP** hydrogel (*n* = 3). (E, F) Schemas of giving 680 nm irradiation at 0, 1, 2, 24 h and Ag^+^ release after different treatment (*n* = 3). (G–J) The CFU of *S. mutans* treated with PBS, **FNP**, **ZBAg@FNP** with irradiation at different time point (*n* = 3). D2: Dark for 2 h. L+D2: Dark for 2 h with 5 min light at initiation. D1+L+D2: Dark for 2 h with 5 min light at mid‐hour. D2+L: Dark for 2 h with 5 min light at the post‐2 h. D24: Dark for 24 h. L+D24: Dark for 24 h with 5 min light at initiation. All statistical data are presented as mean ± standard deviation (SD).

The biodegradation and biocompatibility of hydrogels are critical for their biomedical applications, and have received considerable attention in recent years [59]. Potential toxicity may limit the application of hydrogels if refractory components remain in vivo for a long time. To evaluate the in vivo degradation performance and biocompatibility of the hydrogels, 100 µL of PBS, or the **ZBAg** or **ZBAg@FNP** hydrogels were subcutaneously injected into the backs of BALB/c mice. The PBS disappeared quickly after injection, whereas the **ZBAg** and **ZBAg@FNP** hydrogels were subcutaneously maintained for seven and nine days, respectively (Figure 4A). Hematoxylin and eosin (H&E) staining was performed to examine the compatibility of the **ZBAg** and **ZBAg@FNP** hydrogels. No obvious infection or inflammation was detected in the tissues around the injection sites (Figure 4B). In addition, we used in vivo imaging system to visualize the degradation of the **ZBAg@FNP** hydrogel. As **FNP** enables in vivo fluorescence imaging, a strong fluorescent signal was observed after subcutaneous injection of 100 µL of the **ZBAg@FNP** hydrogel in BALB/c mice (Figure S30). Over time, the fluorescence of the **ZBAg@FNP** hydrogel in vivo gradually weakened, becoming barely detectable after 9 days. These observations indicate that the **ZBAg@FNP** hydrogel was fully degraded within 9 days, which is consistent with the results from the subcutaneous hydrogel degradation experiment. A CCK‐8 assay on L929 cells was performed to further investigate the in vitro biocompatibility of the hydrogels. Compared to the control samples, the **FNP**, **ZBAg** and **ZBAg@FNP** samples exhibited negligible cytotoxicity, whereas the Ag^+^ samples cocultured with L929 cells showed less than 50% relative cell viability, suggesting that the **ZBAg** hydrogel reduces the toxicity of Ag^+^ through the slow release of ions (Figure 4C). Collectively, these results confirm that the **ZBAg@FNP** hydrogel has excellent compatibility and long‐term release capability in vivo.

### 2.6 The Synergistic Effect of Local Heat and Ag^+^ Release From the Hydrogel System

Due to the excellent antibacterial properties of Ag^+^, the release kinetics of Ag^+^ from the **ZBAg@FNP** hydrogel were investigated. X‐ray photoelectron spectroscopy (XPS) confirmed the oxidation state of silver in **ZBAg** hydrogel is consistently corresponded to Ag^+^ in AgNO_3_ (Figure S31). Subsequently, the cumulative amount of Ag^+^ released from the **ZBAg@FNP** hydrogels was investigated. The results implied that the **ZBAg@FNP** hydrogel could act as a carrier of Ag^+^ and continuously release the active antibacterial ingredient Ag^+^ (Figure 4D). An initial burst release of Ag^+^ (cumulative release: 26.05%) from the **ZBAg@FNP** hydrogel was observed within the first 2 h under NIR irradiation, followed by a slower release. In contrast, significantly less release occurred without NIR irradiation (cumulative release: 1.55%), indicating that NIR irradiation promoted rapid release of Ag^+^ from the **ZBAg@FNP** hydrogel. After this initial burst, both groups showed sustained release kinetics, with the NIR(+) group maintaining a higher release rate than the NIR(‐) group throughout the 120 h observation period (cumulative releases: NIR(+) = 54.12%; NIR(‐) = 11.09%). The results demonstrated that under NIR irradiation, local heat disrupted Ag^+^‐mediated coordination and accelerated the short‐term rapid release of Ag^+^, whereas, in the absence of light irradiation, the coordination interaction between Ag^+^ and **Z** in the hydrogel enabled long‐term sustained release, reducing premature leakage of Ag^+^ while mitigating Ag^+^‐induced cytotoxicity. The characteristics of the short‐term rapid release and long‐term stable release of the **ZBAg@FNP** hydrogel under NIR irradiation have important applications in bacterial eradication control.

To systematically analyze the optimized synergistic action of heat and Ag^+^ release in the **ZBAg@FNP** hydrogel, we monitored Ag^+^ release under controlled NIR exposure protocols (the treatments of NIR irradiation are marked as L: light; D: dark) at designated intervals during bacterial incubation (0, 1, and 2 h). As illustrated in Figure 4E, the L + D2 group achieved greater Ag^+^ release than the other groups (D2, D1+L+D1, D2+L), and the sequential release quantification revealed a distinct hierarchy: L+D2> D1+L+D1> D2+L > D2, further indicating that local heat triggered by the photothermal effect accelerated the release of Ag^+^. Additionally, the earlier the local heating occurred, the greater the quantity of Ag^+^ released. This occurred probably because the **ZBAg@FNP** hydrogel can rapidly convert light energy into heat energy under NIR light at 680 nm, and the increase in local heat caused by the photothermal effect in **FNP** accelerated the vibration of Ag^+^ and the disruption of the 3D networks of the hydrogel, thus increasing the release rate of Ag^+^. Under early‐stage irradiation (L + D2), the hydrogel undergoes immediate structural disruption. Throughout the subsequent 2 h period, Ag⁺ release occurs under this persistently disrupted state of hydrogel, resulting in elevated Ag⁺ release levels. In contrast, delayed irradiation (D1 + L + D1) preserved the hydrogel's intact structure during the pre‐irradiation phase (1 h), resulting in minimal Ag⁺ release in this 1 h period. Consequently, the cumulative Ag⁺ release over 2 h in D1 + L + D1 group was significantly reduced compared to the early irradiation group (L + D2). To probe the temporal synergy of photothermal heating and sustained long‐term release of Ag^+^, the Ag^+^ release time of the **ZBAg@FNP** hydrogel was extended to 24 h. Similarly, the L + D24 group had greater Ag^+^ release than the D24 group (Figure 4F). These results demonstrated that the local heat triggered by the photothermal effect accelerated short‐term and long‐term Ag^+^ release; the earlier the local heating occurred, the greater the quantity of Ag^+^ released.

To further elucidate the synergistic effect of localized heating and Ag^+^ release on antibacterial efficacy, the antibacterial activity of the **ZBAg@FNP** hydrogel against *S. mutans* was evaluated by different NIR irradiation treatments. Compared to the other groups, the L + D24 group showed greater antibacterial effects (Figure 4G,H). Assessment of the **FNP** and **ZBAg@FNP** hydrogels under identical irradiation conditions (L+D2 and L+D24) highlighted the enhanced antimicrobial capacity of long‐term Ag^+^ release. For *S. mutans*, the **ZBAg@FNP** hydrogel showed greater bacterial killing efficiency than **FNP** after L + D2 treatment. This disparity in performance increased with prolonged treatment (L + D24) (Figure 4I,J), with *S. mutans* biofilm assays confirming this trend (Figure S32). Therefore, the results revealed the synergistic advantages of photothermal activation and long‐term sustained release of Ag^+^ in enhancing antibacterial efficacy.

### 2.7 Systematic Evaluation of the Antibacterial and Antibiofilm Activities of the ZBAg@FNP Hydrogel In Vitro

**FIGURE 5:**
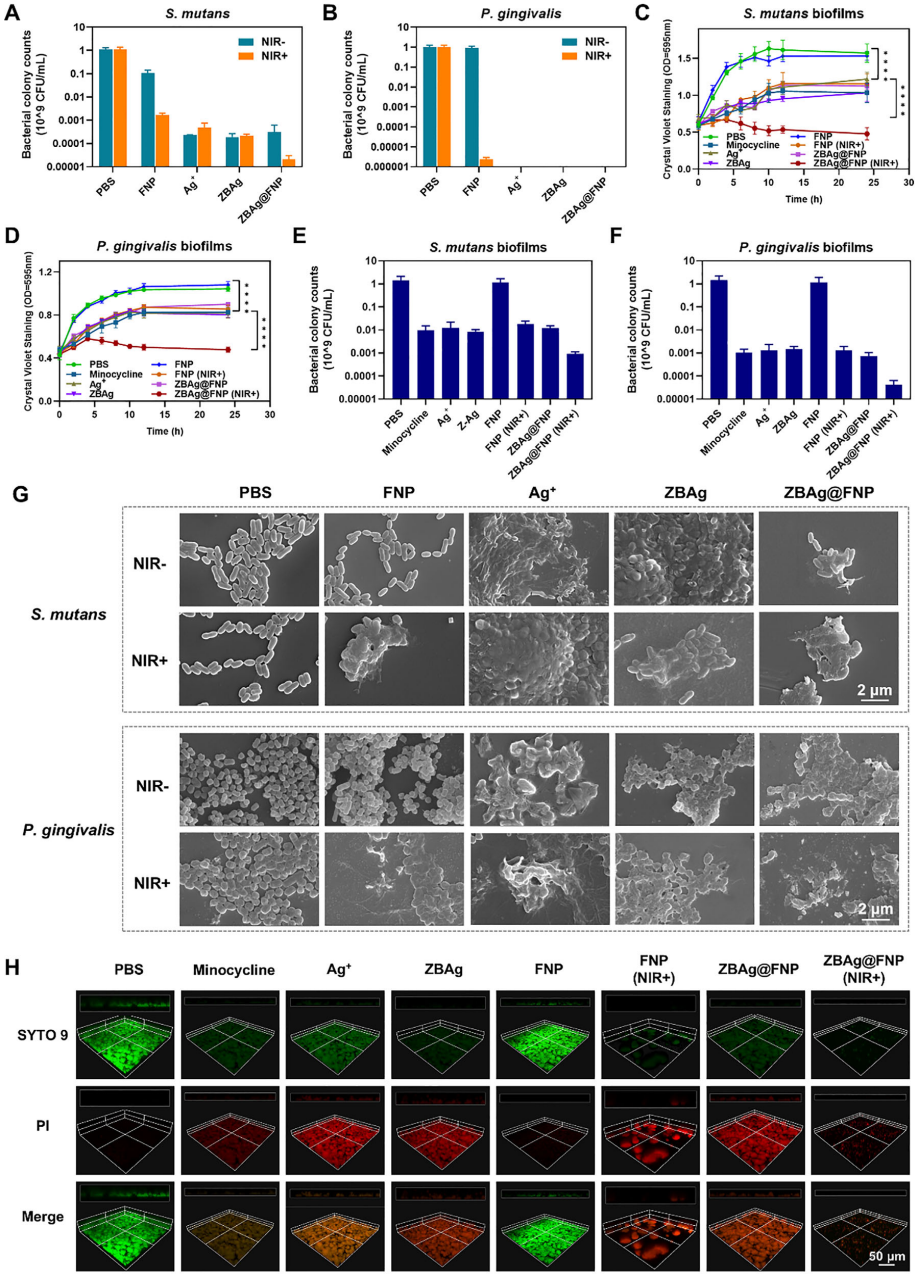
Antibacteria and antibiofilm effects of ZBAg@FNP hydrogel. (A, B) Bacterial colony counts of *S. mutans* and *P. gingivalis* treated with PBS, **FNP**, Ag^+^, **ZBAg**, and **ZBAg@FNP** for 24 h. (C, D) The growth curves of *S. mutans* and *P. gingivalis* biofilms by crystalline violet staining. (E, F) Bacterial colony counts of *S. mutans* and *P. gingivalis* biofilms treated with PBS, minocycline, **FNP**, Ag^+^, **ZBAg**, and **ZBAg@FNP** for 24 h. (G) SEM images of *S. mutans* and *P. gingivalis* treated with PBS, **FNP**, Ag^+^, **ZBAg**, and **ZBAg@FNP** for 24 h (scale bar: 2 µm) (H) CLSM images of *S. mutans* biofilms treated with PBS, minocycline, **FNP**, Ag^+^, **ZBAg**, and **ZBAg@FNP** by SYTO 9 and PI staining for 24 h (scale bar: 50 µm). Data presented as mean ± SD, *n* = 3, *p*‐values are calculated using one‐way ANOVA, ^****^ *p* < 0.0001.

To systematically assess the antibacterial activities of the **ZBAg@FNP** hydrogel against typical oral pathogens (*S. mutans* and *P. gingivalis*), six groups, namely, PBS, minocycline, Ag^+^, **FNP**, **ZBAg,** and **ZBAg@FNP** with or without NIR irradiation, were used in our study. Quantitative analysis using plate colony‐counting methods revealed that **FNP** only exhibited antibacterial effects under NIR irradiation, indicating that **FNP** possessed certain antibacterial activity through their photothermal effects (Figure 5A,B; Figure S33). The Ag^+^, **ZBAg,** and **ZBAg@FNP** groups demonstrated antibacterial effects under light and dark conditions, further confirming the superior antibacterial efficacy of Ag^+^. The photothermal effect generated by **FNP** under NIR irradiation also demonstrated antibacterial efficacy. The **ZBAg@FNP** hydrogel group treated with the NIR laser demonstrated superior antibacterial activity against planktonic bacteria compared to all other groups, suggesting that the enhanced efficacy is attributed to the synergistic interactions between localized photothermal heating and controlled Ag^+^ release. The live/dead staining results further revealed intense red fluorescence in bacterial cells in the **ZBAg@FNP** (NIR+) group, indicating significant membrane compromise and cell death (Figures S34 and S35). The SEM images revealed that the *S. mutans* and *P. gingivalis* in the PBS and **FNP** (NIR‐) groups maintained an intact bacterial morphology, whereas membrane disruption and cytoplasmic leakage were observed in the Ag^+^, **FNP** (NIR+), **ZBAg,** and **ZBAg@FNP** groups (Figure 5G). **ZBAg@FNP** (NIR+) exhibited the most pronounced structural damage and cytoplasm leakage, confirming the combinatorial bactericidal mechanism of PTT and Ag^+^ release.

As the treatment of periodontitis primarily involves removing bacterial biofilms to control infection and inflammation, we investigated the effectiveness of the **ZBAg@FNP** hydrogel against biofilms formed by *S. mutans* and *P. gingivalis*. The growth curves of the biofilms formed by *S. mutans* and *P. gingivalis* were quantified by crystal violet staining. The groups treated with minocycline, **FNP** (NIR+), Ag^+^, **ZBAg**, **ZBAg@FNP**, and **ZBAg@FNP** (NIR+) showed greater antibiofilm effects than the PBS group (Figure 5C,D). The biofilms in the **ZBAg@FNP** (NIR+) group treated with the NIR laser demonstrated stable inhibitory effects after 24 h, while the biofilms in the other treatment groups increased significantly after 2 h, which confirmed the synergistic antibiofilm effect of PTT and the sustained release of Ag^+^. The quantitative analysis of the plate colony counting method and the results of live/dead staining validated the above findings. For *S. mutans* biofilms, minocycline, **FNP** (NIR+), Ag^+^, **ZBAg**, and **ZBAg@FNP** treatments reduced bacterial colonies by about two orders of magnitude compared to the PBS group, while the reduction in the **ZBAg@FNP** (NIR+) group was significantly greater (about three orders of magnitude) (Figure 5E). For *P. gingivalis* biofilms, the above treatment groups (minocycline, **FNP** (NIR+), Ag^+^, **ZBAg**, and **ZBAg@FNP**) all reduced bacterial colonies by about three orders of magnitude compared to the PBS group, but the **ZBAg@FNP** hydrogel group had more stable and significant antibacterial effects (about four orders of magnitude reduction) (Figure 5F). The live/dead staining results revealed that the **FNP** (NIR+), Ag^+^, **ZBAg**, **ZBAg@FNP**, and **ZBAg@FNP** (NIR+) treatment groups presented red fluorescence, and the biofilms in the **FNP** (NIR+) and **ZBAg@FNP** (NIR+) groups were disrupted by PTT (Figure 5H; Figure S36). The above results confirmed that **ZBAg@FNP** (NIR+) exhibited a superior antibiofilm effect, and the enhanced efficacy was attributed to the synergistic interplay between PTT and controlled Ag^+^ release.

**FIGURE 6:**
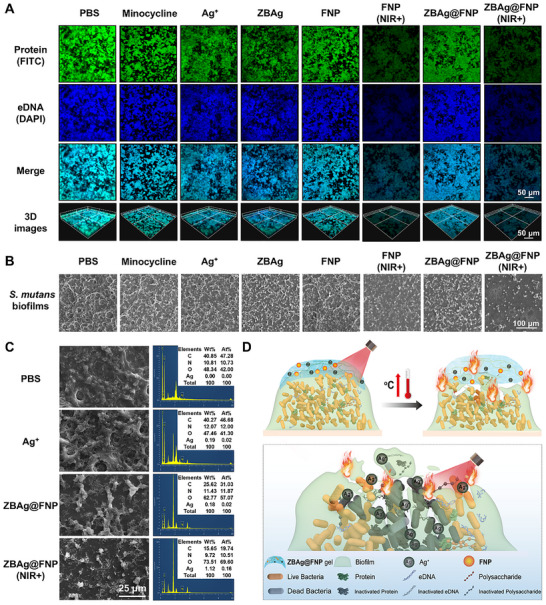
Antibiofilm mechanism of ZBAg@FNP hydrogel. (A) CLSM images of *S. mutans* biofilms treated with PBS, minocycline, **FNP**, Ag^+^, **ZBAg**, and **ZBAg@FNP** by FITC and DAPI staining for 24 h (scale bar: 50 µm). (B) SEM images of *S. mutans* biofilms treated with PBS, minocycline, **FNP**, Ag^+^, **ZBAg**, and **ZBAg@FNP** for 24 h (scale bar: 100 µm). (C) SEM images and corresponding EDS of *S. mutans* biofilms treated with PBS, Ag^+^, **ZBAg** and **ZBAg@FNP** for 2 h (scale bar: 25 µm). (K) Antibiofilm mechanism of **ZBAg@FNP** hydrogel with the synergistic effect of PTT and Ag^+^ release.

To further investigate the synergistic antibiofilm mechanism of the **ZBAg@FNP** hydrogel, the *S. mutans* biofilms were photographed using confocal laser scanning microscopy (CLSM) and SEM. Proteins and eDNA within the biofilms were fluorescently labeled with FITC and DAPI. At 24 h post‐treatment, proteins and eDNA in the **FNP** (NIR+) and **ZBAg@FNP** (NIR+) groups were significantly lower than those in the other groups (Figure 6A), which confirmed that the photothermal effects of **FNP** and **ZBAg@FNP** were better than the antibiofilm effect under light conditions by eradicating proteins and eDNA in the EPS. The polysaccharide staining results supported these findings (Figure S37). The SEM results revealed biofilm reduction in all treatment groups compared to the PBS group (Figure 6B). The **FNP** (NIR+) and **ZBAg@FNP** (NIR+) groups presented more pronounced biofilm reductions than the other treatment groups, indicating that enhanced antibiofilm eradication was mediated by the photothermal effect. The **ZBAg@FNP** (NIR+) group showed the most significant biofilm eradication, which validated the synergistic enhancement of antibiofilm effects through the combination of PTT and the sustained release of Ag^+^. To determine whether localized heat enhances Ag^+^ penetration into biofilms, energy‐dispersive spectroscopy (EDS) analysis was performed. As shown in Figure 6C, biofilms treated with PBS presented no detectable Ag signal (0.00%), whereas those treated with NIR‐activated **ZBAg@FNP** contained 0.16% Ag, which was significantly greater than that of the Ag^+^ solution group (0.02%) and non‐irradiated **ZBAg@FNP** group (0.02%). These results confirmed that photothermally generated localized heat facilitates the infiltration of Ag^+^ into biofilms.

The antibiofilm mechanism of the **ZBAg@FNP** hydrogel is summarized in Figure 6D. First, the photothermal effects of **ZBAg@FNP** strongly inactivate protein and eDNA in biofilms and destabilize the integrity of biofilms, leading to further permeation by released Ag^+^. Moreover, localized thermal energy accelerates the release of Ag^+^ from the hydrogel matrix. The released Ag^+^ ions penetrate the compromised biofilm structure, disrupt bacterial membranes and increase antibacterial efficacy. The synergistic interplay between photothermal disruption and Ag^+^‐mediated antibacterial action establishes a robust antibiofilm strategy, offering mechanistic insights for optimizing the design of antibiofilm materials.

### 2.8 Periodontitis Treatment of Hydrogels In Vivo

**FIGURE 7:**
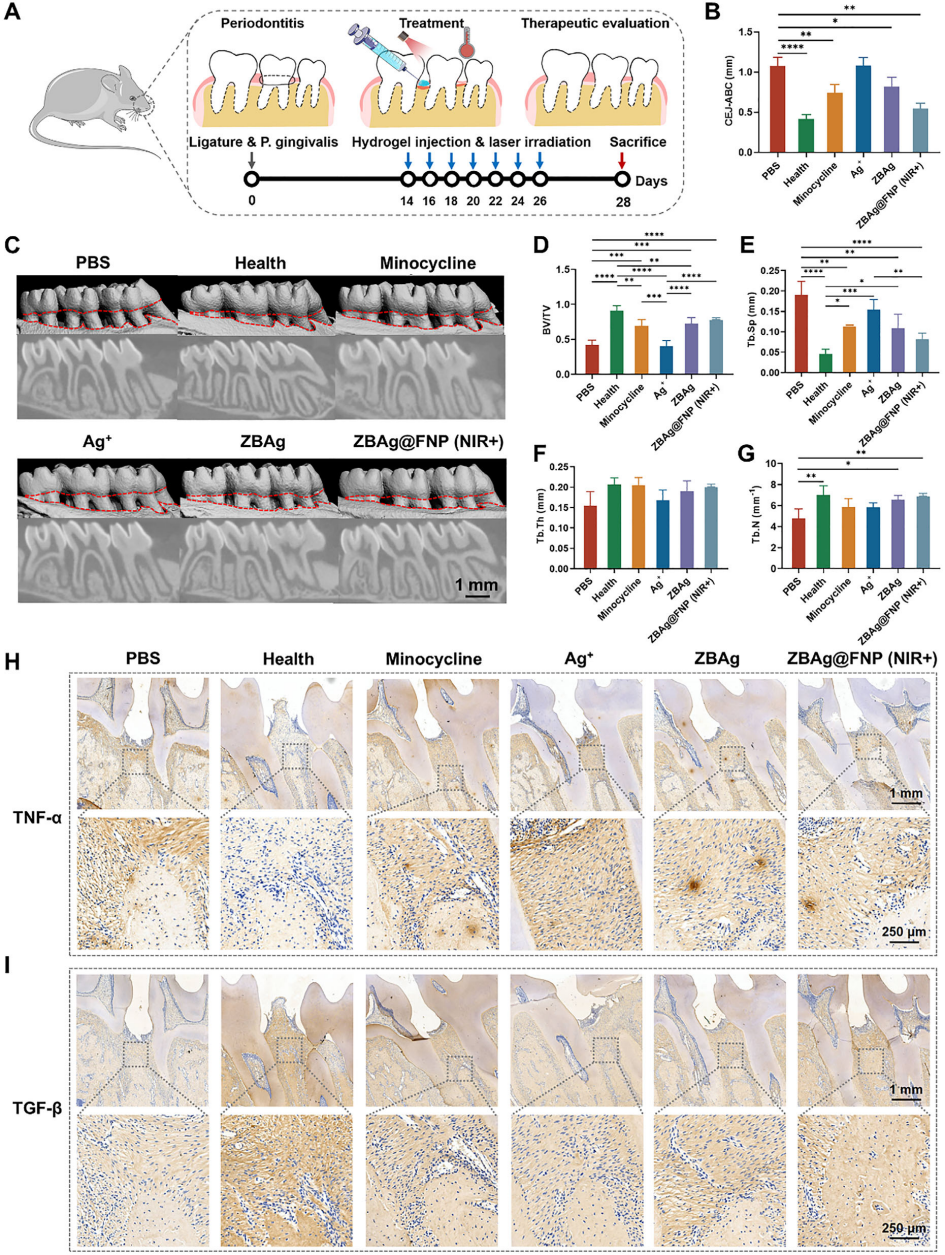
Therapeutic effect of ZBAg@FNP hydrogel in periodontitis. (A) Experimental design illustrating the timeline of periodontitis treatment in this study. (B) Quantitative assessment of the linear measurement between the alveolar bone crest (ABC) and cemento‐enamel junction (CEJ). (C) 3D reconstruction images of the maxillary molars under various treatments through Micro‐CT. The red lines denote the ABC‐CEJ spatial relationship (scale bar: 1 mm). (D) Quantitative analysis of bone volume to total volume ratio (BV/TV). (E) Quantitative analysis of trabecular separation (Tb.Sp). (F) Quantitative analysis of trabecular thickness (Tb.Th). (G) Quantitative analysis of trabecular number (Tb.N). (H) TNF‐α staining of periodontal tissue sections between the first and second molars of the maxilla (scale bars: 1 mm, 250 µm). (I) TGF‐β staining of periodontal tissue sections between the first and second molars of the maxilla (scale bars: 1 mm, 250 µm). Data presented as mean ± SD, *n* = 6, *p*‐values are calculated using one‐way ANOVA, ^*^ *p* < 0.05, ^**^ *p* < 0.01, ^***^ *p* < 0.001, ^****^ *p* < 0.0001.

A periodontitis rat model was established to evaluate the therapeutic effects of the **ZBAg@FNP** hydrogel on periodontitis. The model used in this study was approved by the West China Hospital of Stomatology, Sichuan University (WCHSIRB‐D‐2024‐627). Following acclimation for seven days, periodontitis was induced in the rats by placing 3–0 silk ligatures combined with the inoculation of *P. gingivalis*. At 14 days post‐infection, various treatments, including 20 µL of PBS, minocycline, Ag^+^, **ZBAg** hydrogel, and **ZBAg@FNP** hydrogel, were administered via injection into the periodontal pockets. The periodontal pockets in the **ZBAg@FNP** hydrogel group were irradiated with a 680 nm, 1.0 W cm^−2^ laser for 5 min, and the irradiation and administration treatments were repeated every other day for 14 days, followed by comprehensive evaluations via micro‐CT, H&E staining, Masson's trichrome staining, and TNF‐α/TGF‐β immunohistochemistry (IHC) to assess antimicrobial and regenerative outcomes (Figure 7A).

Micro‐CT analysis of the cross‐sections of the alveolar bone tissue revealed the presence of a hypointense shadow indicative of bone resorption surrounding the second molar. The PBS control group exhibited bone resorption extending to the first and third molars. Although the minocycline and **ZBAg** treatments improved bone preservation, the **ZBAg@FNP** treatment resulted in few detectable low‐density shadows on radiographic images; its therapeutic efficacy was higher than that of the minocycline and **ZBAg** treatments (Figure 7C). Quantitative analysis of the distance from the alveolar bone crest to the cementoenamel junction (ABC‐CEJ) revealed a significantly greater ABC‐CEJ distance in the PBS group, indicating considerable alveolar bone resorption, whereas the **ZBAg@FNP** hydrogel group treated with the NIR laser exhibited minimal alveolar bone resorption or reduction (Figure 7B). The quantitative morphometric parameters, bone volume to total volume ratio (BV/TV), trabecular separation (Tb.Sp), trabecular thickness (Tb.Th), and trabecular number (Tb.N), confirmed these findings. As shown in Figure 7D–G, treatment of the **ZBAg@FNP** hydrogel effectively mitigated bone loss and increased the Tb.Th and Tb.N. H&E staining (Figure S38) revealed a significant reduction in inflammatory infiltration in the treated groups.

Masson's trichrome staining (Figure S38) revealed enhanced collagen organization and improved tissue integrity, which indicated active repair mechanisms. IHC results for TNF‐α (Figure 7H) and TGF‐β (Figure 7I) demonstrated that the expression of TNF‐α, a marker of inflammation, was significantly lower in the **ZBAg@FNP** hydrogel treatment group than in the control group, whereas the expression of TGF‐β, a marker of tissue repair and regeneration, increased considerably. Quantitative analysis (Figures S39 and S40) revealed that the average optical density (AOD) and percentage of TNF‐α‐positive cells were significantly lower in the **ZBAg@FNP** hydrogel treatment group than in the control group, whereas the expression of TGF‐β increased significantly, suggesting effective suppression of inflammation and promotion of tissue healing. Additionally, no inflammatory responses were detected in the tissues, indicating the excellent biocompatibility of the **ZBAg@FNP** hydrogel in vivo (Figure S41). To summarize, the **ZBAg@FNP** hydrogel effectively eradicated biofilms through the synergistic action of PTT and the sustained release of Ag^+^ and significantly reduced alveolar bone resorption, suppressed inflammation, and enhanced tissue regeneration, collectively achieving optimal therapeutic outcomes in periodontitis treatment.

## 3 Conclusion

In this study, we designed photothermal reagents (**BF)** with a PCE of 38.74%, and developed a nucleoside supramolecular hydrogel (**ZBAg**) through silver ion‐stabilized base pairing and dynamic boronate ester bonds, which involved in multiple interactions, including hydrogen bonds, *π*–*π* stacking, coordination and electrostatic interactions. **FNP** was encapsulated into the **ZBAg** hydrogel to prepare **ZBAg@FNP** hydrogel, which was biocompatible and exhibited controlled release of Ag^+^ triggered by local heat. The **ZBAg@FNP** hydrogel showed excellent antibiofilm activity against the biofilms of *P. gingivalis* and *S. mutans*. Its antibiofilm mechanisms involve the hydrogel inactivating EPS components (including proteins and eDNA) through localized hyperthermia, disrupting biofilm architecture, and enhancing the permeability of Ag^+^ into biofilms. In a periodontitis rat model, **ZBAg@FNP** significantly suppressed inflammation, promoted alveolar bone regeneration, and restored tissue integrity, demonstrating promising therapeutic efficacy against periodontal disease. A comparison and in‐depth discussion of the **ZBAg@FNP** hydrogel with other similar studies are also presented (for discussion, see Tables S1 and S2). In conclusion, an innovative sequential antibiofilm strategy was realized through a facile and green fabricated **ZBAg@FNP** hydrogel system, which enhanced biofilm eradication by synergizing PTT with the controlled release of Ag^+^. This strategy not only offers new insights into the design of photothermal agents and antibiofilm materials, but also reveals a promising strategy for periodontal clinical applications.

## 4 Experimental Section

### 4.1 Materials

(4,8‐bis((2‐octyldodecyl)oxy) benzo [1,2‐b:4,5‐b'] dithiophene‐2,6‐diyl) bis (trimethylstannane) (Compounds **1**), 2‐(5,6‐difluoro‐3‐oxo‐2,3‐dihydro‐1*H*‐inden‐1‐ylidene)‐malononitrile (Compounds **4**), and 2‐(5,6‐dichloro‐3‐oxo‐2,3‐dihydro‐1H‐inden‐1‐yl‐idene)malononitrile (Compounds **5**) were purchased from Zhiyan (Nanjing, China), and 5‐bromo‐4‐((2‐ethylhexyl)oxy)thiophene‐2‐carbaldehyde (Component **2)** was purchased from Bide (Shanghai, China). Tetrakis (triphenylphosphine) palladium was purchased from Merck (Germany). Toluene, pyridine, dichloromethane, and tetrahydrofuran (THF) were purchased from Dingsheng (Chengdu, China). Moreover, 2‐aminoadenosine (**Z**) was purchased from TCI Shanghai (Shanghai, China). AgNO_3_ was purchased from Sinopharm (Shanghai, China). CsOH and B(OH)_3_ were purchased from J&K Scientific (Beijing, China). Dulbecco's modified Eagle's medium (DMEM), fetal bovine serum (FBS), and penicillin‐streptomycin were purchased from Gibco (USA). A Cell Counting Kit‐8 (CCK8) was purchased from DOJINDO Molecular Technology (Japan). A brain‐heart infusion (BHI) was purchased from BD Difco (USA). Menadione and hemin were purchased from Sigma–Aldrich (USA). All purchased chemical reagents were of analytical grades and were used without further purification.

### 4.2 Synthesis of **BF** and **BCl**

Under a nitrogen atmosphere, compound **1** (500 mg, 1 eq), compound **2** (287.4 mg, 2 eq), tetrakis(triphenylphosphine)palladium (104 mg, 0.2 eq), and 20 mL of anhydrous and degassed toluene were added to a two‐necked flask. The mixture was heated to 120°C under condensed reflux for 24 h, and the reaction progress was monitored by thin‐layer chromatography (TLC) using petroleum ether (PE) and dichloromethane (DCM) (2:1, Rf = 0.4). After completion, a small amount of deionized water was added. The product was extracted with DCM, dried over anhydrous sodium sulfate, filtered, and concentrated to yield the crude product. The intermediate 5,5'‐(4‐((2‐ethylhexyl)oxy)‐8‐((2‐octyldodecyl)oxy) benzo [1,2‐b:4,5‐b']d‐ithiophene‐2,6‐diyl) bis (4‐((2‐ethylhexyl)oxy) thiophene‐2‐carbaldehyde) (compound **3**) was purified via column chromatography (PE: DCM = 2:1) to afford compounds **3** (312.4 mg, 55.0% yield) (Figure S1). ^1^H NMR (600 MHz, CDCl_3_) δ: 9.81 (s, 2H), 7.83 (s, 2H), 7.53 (s, 2H), 4.22‐4.21 (d, *J* = 6.0 Hz, 4H), 4.17‐4.11 (m, 4H), 1.92‐1.84 (m, 4H), 1.36‐1.15 (m, 80H), 0.88‐0.83 (m, 24H) ppm (Figure S4).

In a separate procedure under nitrogen, compound **4** (200 mg, 4 eq), compound **3** (274 mg, 1 eq), 1 mL of anhydrous pyridine, and 20 mL of anhydrous chloroform were added to a two‐necked flask. The reaction mixture was refluxed at 65°C for 3.5 h, with progress monitored by TLC (PE: DCM = 3:2, R_f_ = 0.5). Upon completion, dilute hydrochloric acid was added dropwise, and the mixture was precipitated in 200 mL of methanol for 30 min. The solid was collected by filtration and purified via column chromatography (PE: DCM = 3:2) to afford the target compound **BF** (158.4 mg, 43.2% yield) (Figure S2). ^1^H NMR (600 MHz, CDCl_3_) δ: 8.65 (s, 2H), 8.42‐8.39 (m, 2H), 7.93 (s, 2H), 7.66‐7.64 (m, 2H), 7.57 (s, 2H), 4.24‐4.21 (m, 6H), 4.19‐4.15 (m, 2H), 1.98‐1.92 (m, 4H), 1.34‐1.22 (m, 80H), 0.89‐0.82 (m, 24H) ppm (Figure S5). ^13^C NMR (150 MHz, CDCl_3_) δ 136.43, 134.52, 114.92, 114.32, 112.63, 74.72, 39.80, 39.54, 32.10, 32.09, 31.32, 30.62, 30.33, 29.95, 29.93, 29.91, 29.89, 29.87, 29.86, 29.58, 29.56, 29.52, 29.27, 27.14, 24.20, 23.25, 22.85, 14.30, 14.28, 14.25, 11.27, 0.15 ppm (Figure S7). HRMS (MALDI‐TOF) calculated for C_100_H_126_F_4_N_4_O_6_S_4_: 1682.8496, found: 1681.7992 (Figure S9).

In a separate procedure under nitrogen, compound **5** (200 mg, 4 eq), compound **3** (239 mg, 1 eq), 1 mL of anhydrous pyridine, and 20 mL of anhydrous chloroform were added to a two‐necked flask. The reaction mixture was refluxed at 65°C for 3.5 h, with progress monitored by TLC (PE: DCM = 3:2, Rf = 0.5). Upon completion, dilute hydrochloric acid was added dropwise, and the mixture was precipitated in 200 mL of methanol for 30 min. The solid was collected by filtration and purified via column chromatography (PE: DCM = 3:2) to afford the target compound **BCl** (193.6 mg, 58.3% yield) ppm (Figure S3). ^1^H NMR (600 MHz, CDCl_3_) δ: 8.66 (s, 2H), 8.63 (s, 2H), 7.93‐7.90 (m, 4H), 7.58 (s, 2H), 4.25‐4.21 (m, 6H), 4.19‐4.16 (m, 2H), 1.97‐1.93 (m, 4H), 1.31‐1.24 (m, 80H), 0.87‐0.82 (m, 24H) ppm (Figure S6). ^13^C NMR (150 MHz, CDCl_3_) δ 138.57, 135.95, 126.78, 125.12, 114.40, 74.80, 39.76, 39.54, 32.11, 32.09, 31.30, 30.57, 30.35, 29.97, 29.95, 29.92, 29.90, 29.89, 29.86, 29.59, 29.57, 29.26, 27.14, 24.18, 23.27, 22.86, 22.85, 14.31, 14.27, 11.26, 0.15 ppm (Figure S8). HRMS (MALDI‐TOF) calculated for C_100_H_126_Cl_4_N_4_O_6_S_4_: 1749.7318, found: 1748.5241 (Figure S10).

### 4.3 Preparation and Characterization of **FNP** and **ClNP**

Compound **BF** or **BCl** (1 mg) was dissolved in 1 mL of THF, followed by the addition of 6 mg of Pluronic F‐127. The mixture was sonicated until complete dissolution. Separately, 10 mL of deionized water was placed in a vial with a magnetic stirrer. The F‐127‐containing solution was rapidly injected into the aqueous phase under vigorous stirring. The mixture was stirred at room temperature for 24 h. Then, it was dialyzed for 24 h using dialysis membranes with a molecular weight cutoff (MWCO) of 3.5 kDa, with ddH_2_O exchanged 6 times. The nanoparticle solution was filtered through a 0.45 µm syringe driven filter. Finally, the resulting nanoparticles (**FNP** or **ClNP**) were concentrated using an ultrafiltration tube (MWCO of 10 kDa) at 3000 rpm to remove excess solvent.

The absorption spectra of **FNP** or **ClNP** were recorded using a Cary Series UV–vis spectrophotometer (Agilent, USA), and the fluorescence spectra were measured by a Cary Eclipse fluorescence spectrophotometer (Agilent, USA). The particle sizes were determined using a SZ‐100V2 particle size analyzer (HORIBA, Japan), and the surface morphology was observed using an Inspect F50 scanning electron microscope (FEI, USA).

### 4.4 Preparation of the Hydrogels

(1) **ZBAg** hydrogel: A total of 28.3 mg of **Z** was dissolved in 1.7 mL of ddH_2_O and heated until it was completely dissolved. Then, 0.1 mL each of H_3_BO_3_ (0.5 m), CsOH (0.5 m), and AgNO_3_ (0.5 m) solutions were added and mixed thoroughly. The mixture was subsequently cooled to room temperature, resulting in the formation of the **ZBAg** hydrogel. Hydrogel formation was confirmed by conducting a tube‐inversion test, in which no flow indicated successful gelation. (2) **ZB** sample: A total of 28.3 mg of **Z** was dissolved in 1.8 mL of ddH_2_O and heated until it was completely dissolved. Then, 0.1 mL each of H_3_BO_3_ (0.5 m) and CsOH (0.5 m) solutions were added and mixed thoroughly. The mixture was subsequently cooled to room temperature, resulting in the formation of the **ZB** sample. (3) **ZAg** sample: A total of 28.3 mg of **Z** was dissolved in 1.9 mL of ddH_2_O and heated until it was completely dissolved. Then, 0.1 mL AgNO_3_ (0.5 m) solution were added and mixed thoroughly. The mixture was subsequently cooled to room temperature, resulting in the formation of the **ZAg** sample. (4) **ZBAg@FNP** and **ZBAg@ClNP** hydrogels: During the preparation of **ZBAg** hydrogel, **FNP** and **ClNP** solutions (15 µg mL^−1^) were added into the **ZBAg** hydrogel before cooling, thoroughly mixed, and then cooled to obtain **ZBAg@FNP** and **ZBAg@ClNP** hydrogels respectively.

### 4.5 Nuclear Magnetic Resonance (NMR) Spectroscopy of Hydrogel

Dissolve 10 mg of lyophilized **Z**, **ZAg**, and **ZBAg** in 500 µL of DMSO‐*d*_6_, respectively, and transfer them into an NMR tube. The ^1^H,^11^B, ^13^C, COSY, HSQC, HMBC, NOESY, and variable temperature (VT) ^1^H NMR spectra were acquired at 600 MHz using an AV II spectrometer (Bruker, Germany).

### 4.6 Fourier Transform Infrared Spectroscopy (FTIR) of Hydrogel

The hydrogel was freeze‐dried to obtain a xerogel, which was subsequently analyzed using a Nicolet 6700 FTIR spectrometer (Thermo Fisher Scientific, USA) in the range of 2000–4000 cm^−1^.

### 4.7 Electrospray Ionization Mass Spectrometry (ESI‐MS) of Hydrogel

Dissolve 10 mg of **Z** and **ZBAg** hydrogel in 500 µL ddH_2_O, respectively, followed by ESI‐MS analysis in TSQ Quantum Ultra instrument (Thermo Fisher, USA).

### 4.8 Matrix‐Assisted Laser Desorption/Ionization Time‐of‐Flight (MAIDI‐TOF) of Hydrogel

Dissolve 10 mg of freeze‐dried **Z** or **ZBAg** hydrogel in 0.5 mL of 50% acetonitrile. Separately, dissolve 10 mg of α‐cyano‐4‐hydroxycinnamic acid (CHCA) in 0.5 mL of 50% acetonitrile. Mix equal volumes of the two solutions above. Deposit 5 µL of the mixture onto a well of a 384‐spot ground steel target plate and air‐dry. Acquire MALDI‐TOF mass spectra in positive reflection mode using a MALDI‐8020 instrument (Shimadzu, Japan).

### 4.9 X‐Ray Photoelectron Spectroscopy (XPS) of Hydrogel

The hydrogel was freeze‐dried to obtain a xerogel, which was subsequently analyzed using an AXIS Supra X‐ray photoelectron spectrometer (Kratos, British).

### 4.10 Powder X‐Ray Diffraction (PXRD) of Hydrogel

The freeze‐dried xerogel was analyzed using an X'Pert Pro MPD PXRD diffractometer (Netherlands). PXRD patterns were recorded over a 2*θ* range of 3°–60° with a current of 40 mA and a voltage of 40 kV.

### 4.11 Morphology Characterization of Hydrogel

The morphology of the **ZBAg** hydrogel was investigated using scanning electron microscopy (SEM), atomic force microscopy (AFM), and transmission electron microscopy (TEM). For SEM imaging, hydrogel samples were freeze‐dried into xerogels, mounted on silica wafers, and sputter‐coated with gold. SEM images were obtained using an Inspect F50 scanning electron microscope (FEI, USA). AFM measurements were performed in tapping mode with an amplitude setpoint of 1 V; for these measurements, the hydrogel was diluted 50‐fold with ddH_2_O, and 10 µL of the diluted solution was deposited on a mica plate and air‐dried. TEM observations were conducted using a Talos F200S microscope (Thermo, USA).

### 4.12 Rheological Measurements of Hydrogel

The hydrogel was prepared and immediately transferred to the plate of an MCR302 rheometer (Anton Paar, Graz, Austria) equipped with a 25 mm diameter plate‐plate geometry before cooling. The plate was preheated to 75°C to prevent premature gelation. Silicone oil was applied along the plate edge to prevent the evaporation of water during testing. A frequency sweep measurement was conducted at 25°C with a constant strain of 0.1% over an angular frequency range from 0.1 to 100 rad s^−1^ to determine the elastic modulus (G′) and viscous modulus (G″). A strain sweep test was conducted at an angular frequency of 10 rad s^−1^, where the strain (γ) was gradually increased from 0.1% to 100%. The viscosity was measured under shear strain (γ) ranging from 0.1% to 100%. Additionally, the self‐healing ability of the hydrogel was assessed by alternating between γ values of 0.1% and 100% for four cycles.

### 4.13 Photothermal Performance In Vitro

The sample (200 µL) was exposed to a light laser at 680 nm, and the temperature was recorded every 30 s using a thermal imaging camera (Fluke, USA). The following experiments were conducted: (1) Photothermal reagents and hydrogels at various concentrations (5, 10, 15, 20 µg mL^−1^ **FNP** or **ClNP**) were prepared and subjected to NIR irradiation at 1 W cm^−2^ for 5 min. (2) Photothermal reagents and hydrogels (15 µg mL^−1^ **FNP** or **ClNP**) were exposed to NIR irradiation at different intensities (0.50, 0.75, 1.00, 1.25 W cm^−2^). (3) The hydrogels underwent five cycles of heating and natural cooling induced by NIR irradiation.

The photothermal conversion efficiency (PCE) of **ZBAg@FNP** and **ZBAg@ClNP** hydrogels was determined using the following Equations ((1), (2), (3), (4)).

(1) $$PCE=\frac{hS\left(T_{m}-T_{r}\right)-Q_{0}}{I\left(1-10^{-A680}\right)}$$

(2) $$\tau_{s}=\frac{m_{d}C_{d}}{hS}$$

(3) $$t=-\tau_{s}\ln\left(\frac{T_{c}-T_{r}}{T_{m}-T_{r}}\right)$$

(4) $$Q_{0}=hS\left(T_{m.w}-T_{r}\right)$$

Here, *h* denotes the heat transfer coefficient, and *S* denotes the surface area of the holder. *T*_m_ and *T*_r_ denote the peak and ambient temperatures of the photothermal materials, respectively. *Q*_0_ was measured using pure water. *I* denote the irradiation intensity. A680 denotes the absorbance at 680 nm. *M*_d_ (1 g) and *C*_d_ (4.2 J (g °C)^−1^) represent the mass and specific heat capacity of the sample, respectively. The real‐time temperature during the cooling period (*T*_c_) was recorded by the thermal imager, and *T*_m.w_ represents the maximum temperature attained by pure water under NIR irradiation.

### 4.14 Degradation of the Hydrogel In Vivo

First, the six‐week‐old female BALB/c mice were randomly divided into three groups (*n* = 5). 100 µL of PBS, **ZBAg,** and **ZBAg**@**FNP** hydrogels were injected subcutaneously into the backs of mice. The mice were then euthanized at predetermined time points (0, 1, 3, 5, 7, and 9 days), and the skin containing the residual hydrogel was photographed. The skin samples containing the hydrogel were subsequently removed and fixed in a 4% paraformaldehyde solution. After dehydration for 24 h, the tissues were embedded in paraffin, sectioned, and stained with H&E for histopathological evaluation. Furthermore, 100 µL **ZBAg@FNP** hydrogel was injected subcutaneously for visual assessment the degradation in vivo using Tanon ABL‐X6 (*n* = 5).

### 4.15 Cell Counting Kit‐8 (CCK‐8) Assays for Biological Toxicity

Cell Counting Kit‐8 (CCK‐8) assays were performed to evaluate the cytotoxic effects of the hydrogels on mouse skeletal muscle L929 cells. The cells were cultured in DMEM supplemented with 10% FBS and 1% penicillin‐streptomycin at 37°C in a 5% CO_2_ atmosphere and seeded in 96‐well plates at a density of 1 × 10^4^ cells per well. Once the cells adhered, 50 mg of hydrogel leachate was cocultured with the cells for 24 h. The cell viability was determined by measuring the optical density (OD) at 450 nm using a Varioskan Flash plate reader (Thermo Scientific, USA).

### 4.16 Silver Ion Release

A 1 mL hydrogel sample was prepared in vials, and 1 mL of ddH_2_O was added on top. At specific time intervals, 0.5 mL aliquots of the supernatant were collected for analysis, and an equal volume of fresh ddH_2_O was added to maintain a constant volume. The amount of Ag^+^ released was determined using an inductively coupled plasma emission spectrometer (PerkinElmer Avio200, USA).

### 4.17 Antibacterial Activity Assay

*Streptococcus mutans* (*S. mutans*) was cultured at 37°C in a BHI medium, while *Porphyromonas gingivalis* (*P. gingivalis*) was cultured at 37°C in an atmosphere of 80% N_2_, 10% H_2_, and 10% CO_2_ in a medium consisting of BHI, 1 µg mL^−1^ menadione, and 5 µg mL^−1^ hemin. A 1 mL bacterial suspension (1 × 10^6^ CFU mL^−1^) was transferred to a 15 mL centrifuge tube, after which 50 mg of PBS, AgNO_3_, minocycline, **FNP**, **ZBAg** hydrogel, or **ZBAg@FNP** hydrogel was added. After incubation for 24 h, antibacterial evaluations were performed as follows: (1) The coculture solution was diluted, and 100 µL of the diluted suspension was inoculated on a BHI agar plate and incubated for about three days for colony‐forming unit (CFU) counting. (2) Bacteria were stained using the Live/Dead BacLight Bacterial Viability Kit (L7012; Invitrogen, USA). After staining with SYTO 9 and propidium iodide (PI) for 15 min in the dark and rinsing with PBS, a 5 µL aliquot was placed on glass slides for imaging via confocal laser scanning microscopy (CLSM; FV3000, Olympus, Japan). (3) A small aliquot of the bacterial suspension was deposited on a sterile glass slide and fixed in a 2.5% glutaraldehyde solution for 4 h. After washing three times with PBS, the slides were dehydrated by immersion in a series of ethanol solutions with increasing concentrations (30%, 40%, 50%, 60%, 70%, 80%, 90%, and 100%), each for 10 min. The slides were air‐dried, gold‐coated, and examined via SEM (INSPECT F, FEI, USA) to observe the surface morphology of the bacteria.

### 4.18 Antibiofilm Activity Assay

The bacterial suspension in the logarithmic growth phase was adjusted to 1 × 10^5^ CFU mL^−1^. An 18 mm sterile slide was placed in a 12‐well plate, and 2 mL of the bacterial suspension was added to allow biofilm formation for 48 h. Subsequently, 50 mg of PBS, AgNO_3_, minocycline, **FNP**, the **ZBAg** hydrogel, or the **ZBAg@FNP** hydrogel was added, and the biofilms were further incubated for 24 h to allow treatment. The antibiofilm evaluations included the following steps: (1) The biofilm was collected from the slide, diluted with PBS, inoculated onto a BHI agar plate, and incubated for about three days to count CFU. (2) The biofilm was stained with SYTO 9 and PI, followed by imaging via CLSM. (3) The biofilm was fixed on the slide with 2.5% glutaraldehyde for 4 h, washed with PBS, and dehydrated with increasing concentrations of ethanol solutions (30% to 100% for 10 min each). Then, the slides were air‐dried, gold‐coated, and examined via SEM to examine the surface morphology of the biofilms.

### 4.19 Periodontitis Treatment In Vivo

The clinical model used in this study was approved by the West China Hospital of Stomatology, Sichuan University (WCHSIRB‐D‐2024‐627). All animal procedures conformed to the guidelines of the Ethics Committee for Animal Care and Use. A periodontitis model was established in eight‐week‐old female Sprague–Dawley rats. After acclimation for one week under controlled conditions (21°C, 50% humidity, 12‐h/12‐h light/dark cycle), the rats were anesthetized using isoflurane. A 3–0 silk suture impregnated with *P. gingivalis* was ligated between the first and second maxillary molars to induce periodontitis. Then, 14 days after infection, the rats were randomly divided into five groups (*n* = 6). 20 µL of PBS, minocycline, Ag^+^, the **ZBAg** hydrogel, or the **ZBAg@FNP** hydrogel was injected into the periodontal pockets. In the **ZBAg@FNP** hydrogel group, the periodontal pockets were irradiated with a laser at 680 nm and 1.0 W cm^−2^ for 5 min. Irradiation and treatment were repeated every other day for 14 days. Every three days, the ligatures were examined, and ligatures that became loose or detached were promptly replaced. After 14 days, periodontal tissues from the maxillary molar area were collected and processed for H&E, Masson, and immunohistochemical staining for TNF‐α and TGF‐β, whereas maxillary bone samples were collected for micro‐CT analysis (Quantum GX2, PerkinElmer, USA). Additionally, tissues from the heart, liver, spleen, lungs, and kidneys were obtained and stained with H&E to evaluate toxicity in vivo.

### 4.20 Statistical Analysis

Data are presented as mean ± standard deviation (SD). All quantitative experiments in this study were conducted with a minimum of three replicates. Statistical differences between groups were evaluated using one‐way analysis of variance (ANOVA) in GraphPad Prism 6.0. Significance thresholds were set at *p* < 0.05 (^*^), *p* < 0.01 (^**^), *p* < 0.001 (^***^), and *p* < 0.0001 (^****^), where *p* > 0.05 were not considered statistically significant.

## Conflicts of Interest

The authors declare no conflict of interest.

## Supporting information




**Supporting File**: advs73425‐sup‐0001‐SuppMat.pdf.

## Data Availability

The data that support the findings of this study are available from the corresponding author upon reasonable request.
