# Supplementary material for: Nucleoside‐Based Hydrogel Platform Synergizes with Photothermal Effects for Enhanced Biofilm Eradication Against Periodontitis
Source: Adv Sci (Weinh). 2025 Dec 20;13(13):e22853. doi: 10.1002/advs.202522853 (PMC12955991; doi:10.1002/advs.202522853)
Supplement: Supplementary file 1 — Supporting File: advs73425‐sup‐0001‐SuppMat.pdf. [file ADVS-13-e22853-s001.pdf]

Supporting Information

Nucleoside-based Hydrogel Platform Synergizes with Photothermal Effects for Enhanced Biofilm Eradication against Periodontitis

Yinghui Wen, Yunfei Li, Tiannan Liu, Chongkui Sun, Hang Zhao, Yao Yuan, Shiping Yang, Tingxing Zhao*, and Jiang Liu**

Yinghui Wen, Tiannan Liu, Hang Zhao, Yao Yuan, Jiang Liu*
State Key Laboratory of Oral Diseases & National Center for Stomatology & National Clinical Research Center for Oral Diseases & Research Unit of Oral Carcinogenesis and Management & Chinese Academy of Medical Sciences, West China Hospital of Stomatology, Sichuan University, Chengdu 610041, Sichuan, P. R. China
E-mail: liujiang@scu.edu.cn

Yunfei Li, Tingxing Zhao*
School of Materials and Chemistry, Southwest University of Science and Technology, Mianyang 621010, Sichuan, P. R. China
E-mail: tingxingzhao@swust.edu.cn

Shiping Yang*
Institute of Biomedical Engineering, College of Medicine, Southwest Jiaotong University, Chengdu 610031, Sichuan, P. R. China
E-mail: yspscu@163.com

Chongkui Sun
Department of Geriatrics, Sichuan Provincial People's Hospital, University of Electronic Science and Technology, Chengdu 610072, Sichuan, P. R. China

Yinghui Wen, Yunfei Li, Tiannan Liu contributed equally to this work.

Nucleoside-based Hydrogel Platform Synergizes with Photothermal Effects for Enhanced Biofilm Eradication against Periodontitis

Supplementary Figures 4

Figure S1. Synthesis of compound **3**. 4

Figure S2. Synthesis of **BF**. 4

Figure S3. Synthesis of **BCl**...4

Figure S4. ^{1}H NMR spectrum of compound **3** in $CDCl_3$...5

Figure S5. ^{1}H NMR spectrum of compound **BF** in $CDCl_3$.......................................5

Figure S6. ^{1}H NMR spectrum of compound **BCl** in $CDCl_3$. 6

Figure S7. ^{13}C NMR spectrum of compound **BF** in $CDCl_3$.....................................6

Figure S8. ^{13}C NMR spectrum of compound **BCl** in $CDCl_3$. 7

Figure S9. The MALDI-TOF mass spectra of **BF**. 7

Figure S10. The MALDI-TOF mass spectra of **BCl**. 8

Figure S11. The optical properties characterization of **BF** and **BCl**.........................9

Figure S12. Photograph showing disintegration of **ZAg** hydrogel after 5 minutes...9

Figure S13. The ESI-MS spectra of **Z** and **ZBAg** hydrogel.................................10

Figure S14. The MALDI-TOF spectra of **Z** and **ZBAg** hydrogel.. 10

Figure S15. ^{1}H NMR spectrum of **Z** in DMSO-d_6 and the corresponding ^{1}H NMR signals assignment in **Z** molecular structure...11

Figure S16. ^{13}C NMR spectrum of **Z** in DMSO-d_6 and the corresponding ^{13}C NMR signals assignment in **Z** molecular structure...11

Figure S17. COSY spectrum of **Z** in DMSO-d_6. 12

Figure S18. HSQC spectrum of **Z** in DMSO-d_6. 12

Figure S19. HMBC spectrum of **Z** in DMSO-d_6. 13

Figure S20. ^{1}H NMR titration was performed to determine the chemical shift of **Z** in DMSO-d_6 at the different ratio of **Z**/Ag^+ (from 1:0 to 1:2.4). 14

Figure S21. NOESY spectra of **Z** (A) and **ZAg** (B) in DMSO-d_6..........................15

Figure S22. VT ^{1}H NMR spectra of **Z** (A) and **ZAg** (B) in DMSO-d_6 were recorded from 25 to 95 °C...16

Figure S23. The base pairing and Ag^+ coordination sites of **ZAg**. 17

Figure S24. SEM image of **ZBAg** hydrogel (scale bar: 500 nm). 18

Figure S25. TEM image of **ZBAg** hydrogel (scale bar: 200 nm). 18

Figure S26. The rheological measurements of **ZBAg** and **ZBAg@ClNP** hydrogels. 19

Figure S27. The photothermal performance of **FNP** and **ClNP**.. 20

Figure S28. The photothermal performance of **ZBAg@ClNP** hydrogel. (A) Temperature change curves of **ZBAg@ClNP** hydrogel at a series of concentrations

(5, 10, 15, 20 μg ml^{-1}) under 1.0 W cm^{-2} NIR irradiation. (B) Near-infrared imaging of **ZBAg@ClNP** hydrogel at a series of concentrations (5, 10, 15, 20 μg ml^{-1}) under 1.0 W cm^{-2} NIR irradiation. 21
Figure S29. Temperature elevation of **ZBAg@ClNP** hydrogel (15 μg ml^{-1}, 1 W cm^{-2}) over five cycles of laser irradiation on/off. 22
Figure S30. The *in vivo* fluorescence images of mice at 0, 1 ,3, 5, 7 and 9 days after subcutaneous injection of 100 μL **ZBAg@FNP** hydrogel. 22
Figure S31. XPS spectra of $AgNO_3$ and **ZBAg** hydrogel. 23
Figure S32. The CFU of *S. mutans* biofilms treated with PBS, **FNP**, **ZBAg@FNP** with irradiation at different time point.. 23
Figure S33. Photographs of bacterial colony in agar plate of *S. mutans* and *P. gingivalis* treated with PBS, **FNP**, Ag^+, **ZBAg**, and **ZBAg@FNP** for 24 h. 24
Figure S34. Fluorescence images of *S. mutans* by staining live bacteria (SYTO 9) and dead bacteria (PI) (scale bar: 75 μm). 25
Figure S35. Fluorescence images of *P. gingivalis* by staining live bacteria (SYTO 9) and dead bacteria (PI) (scale bar: 75 μm). 26
Figure S36. CLSM images of *P. gingivalis* biofilms treated with PBS, minocycline, **FNP**, Ag^+, **ZBAg**, and **ZBAg@FNP** for 24 h by staining live bacteria (SYTO 9) and dead bacteria (PI) (scale bar: 50 μm). 27
Figure S37. CLSM images of *S. mutans* biofilms treated with PBS, minocycline, **FNP**, Ag^+, **ZBAg**, and **ZBAg@FNP** for 24 h by staining live bacteria (SYTO 9) and polysaccharide (calcofluor) (scale bar: 50 μm)..28
Figure S38. Histopathological characterization of periodontal tissue sections between the first and second molars of maxilla including hematoxylin and eosin (H&E) and Masson images (scale bars: 1 mm, 250 μm). 29
Figure S39. Quantification of the average optical density (AOD) and positive staining percentage in TNF-α immunohistochemical staining. 29
Figure S40. Quantification of the AOD and positive staining percentage in TGF-β immunohistochemical staining. 30
Figure S41. H&E staining images of organs from periodontitis rats subjected to different treatments (scale bar: 400 μm). 30
Supplementary Discussion 31
Tabel S1. A comparison of the antibacterial photothermal hydrogel in our manuscript with other literature reports. 33
Tabel S2. A comparison of the photothermal agents in our manuscript with other literature reports. 36
References 43

Supplementary Figures

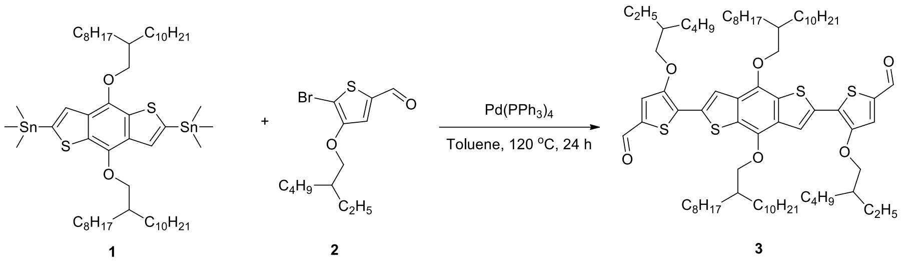

Figure S1. Synthesis of 5,5'-(4-((2-ethylhexyl)oxy)-8-((2-octyldodecyl)oxy) benzo [1,2-b:4,5-b'] dithiophene-2,6-diyl) bis (4-((2-ethylhexyl)oxy) thiophene-2-carbaldehyde) (compound 3).

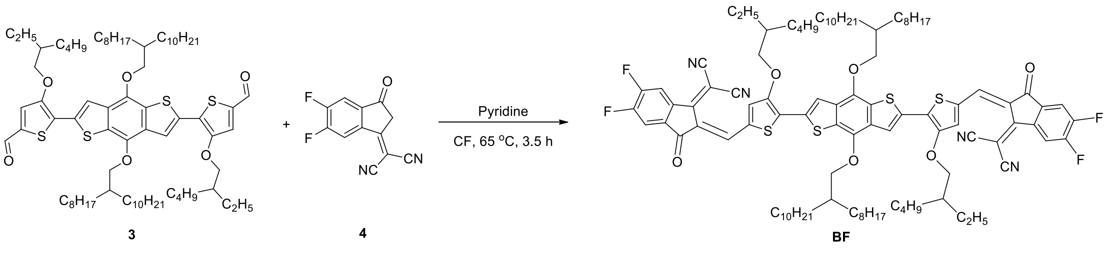

Figure S2. Synthesis of BF.

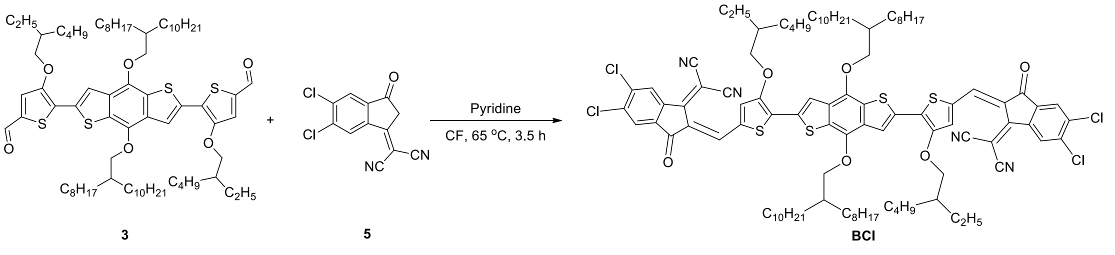

Figure S3. Synthesis of BCl.

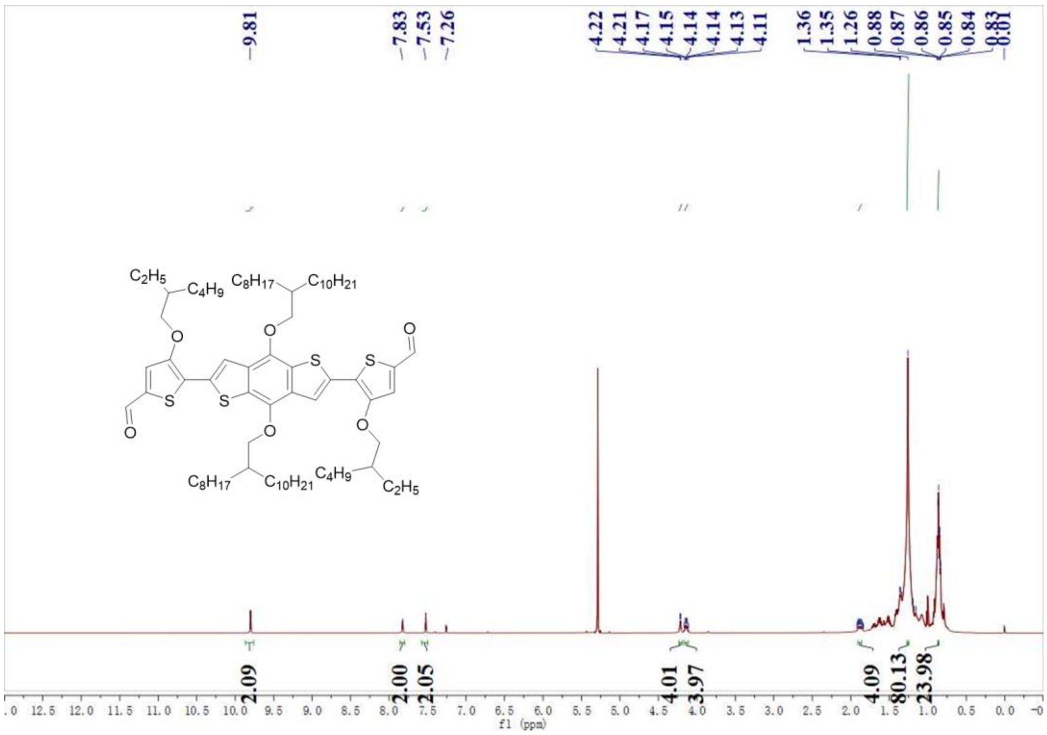

Figure S4. ^{1}H NMR spectrum of 5,5'-(4-((2-ethylhexyl)oxy)-8-((2-octyldodecyl)oxy)benzo[1,2-b:4,5-b']d-ithiophene-2,6-diyl)bis(4-((2-ethylhexyl)oxy)-thiophene-2-carbaldehyde) (compound 3) in $CDCl_3$.

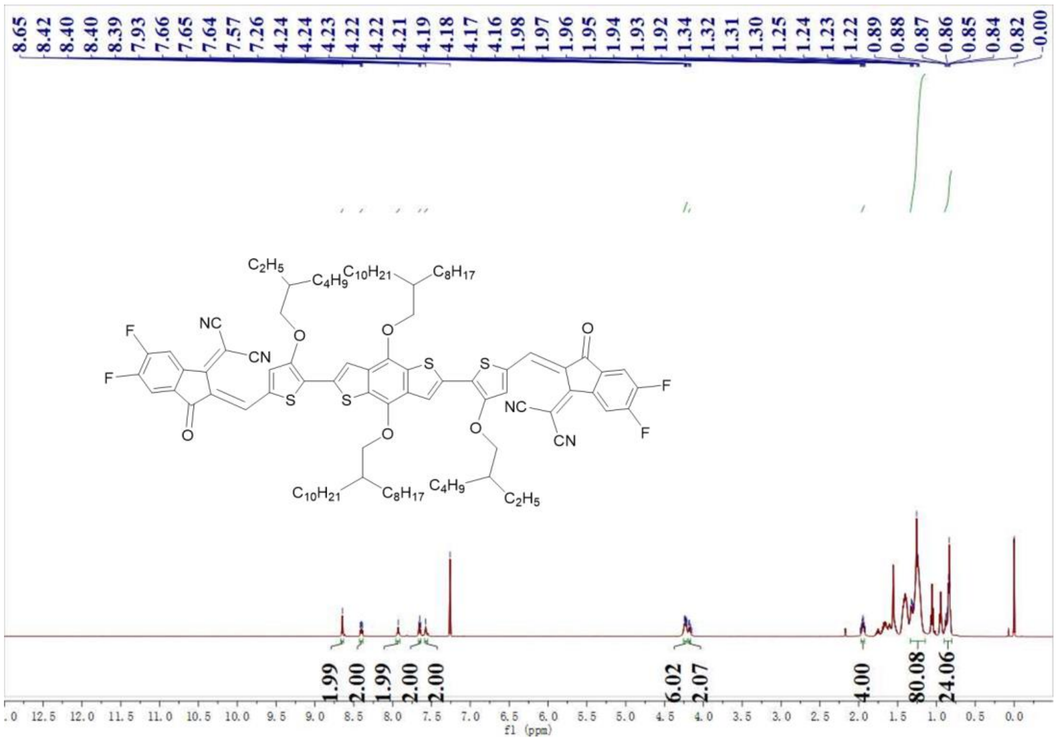

Figure S5. ^{1}H NMR spectrum of compound BF in $CDCl_3$.

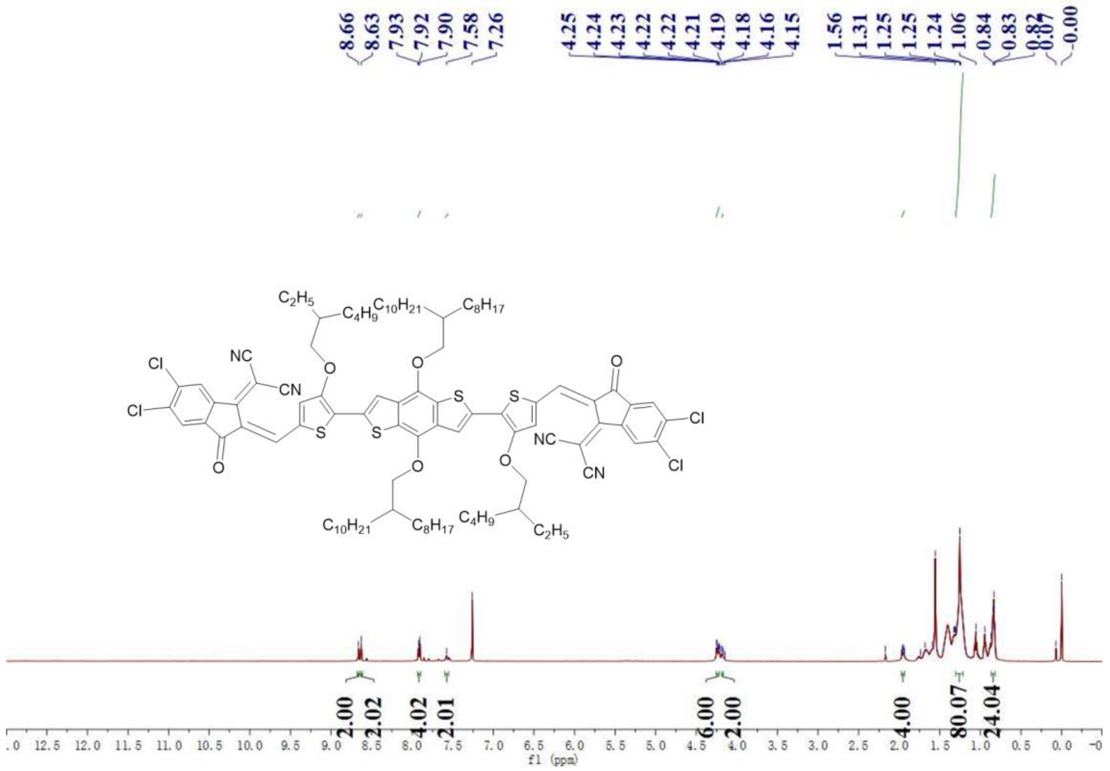

Figure S6. ^{1}H NMR spectrum of compound BCl in $CDCl_3$.

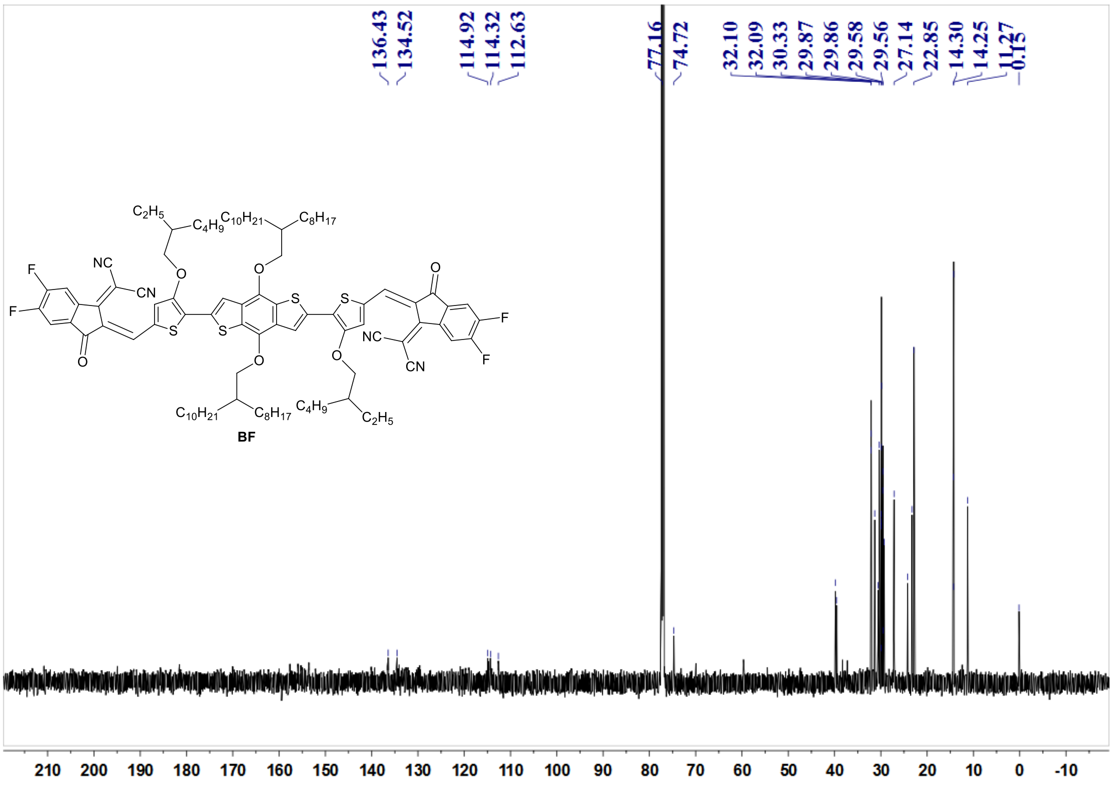

Figure S7. ^{13}C NMR spectrum of compound BF in $CDCl_3$.

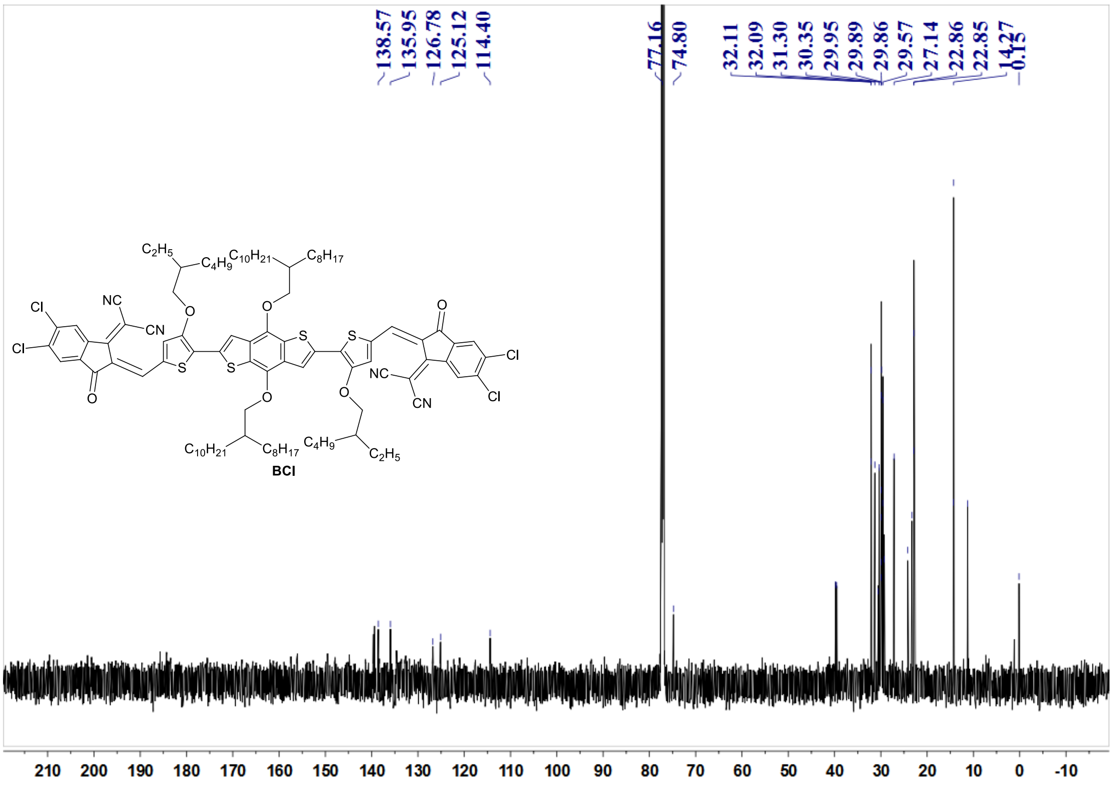

Figure S8. ^{13}C NMR spectrum of compound BCl in $CDCl_3$.

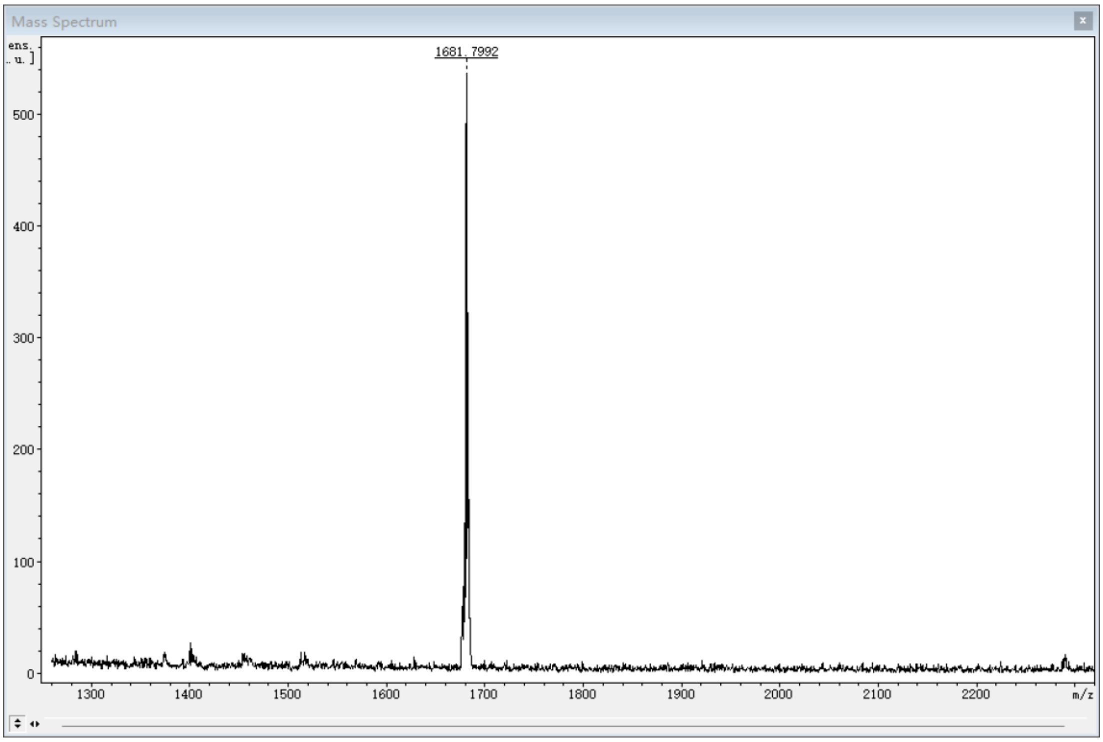

Figure S9. The MALDI-TOF mass spectra of BF.

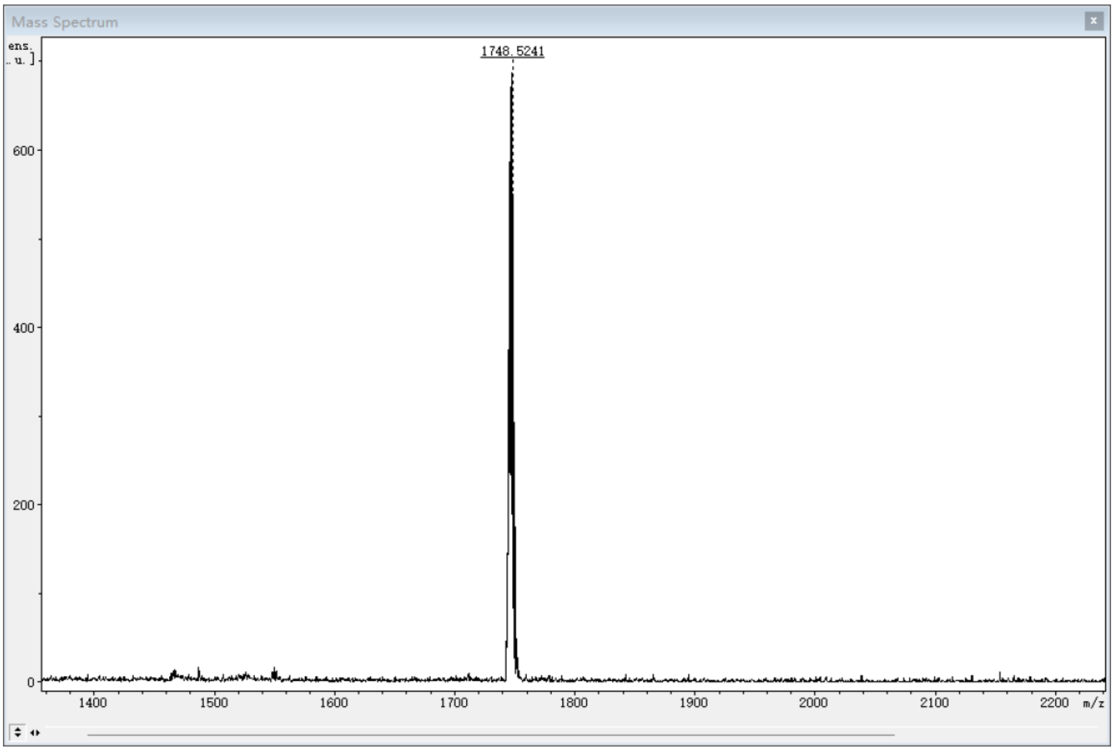

Figure S10. The MALDI-TOF mass spectra of BCl.

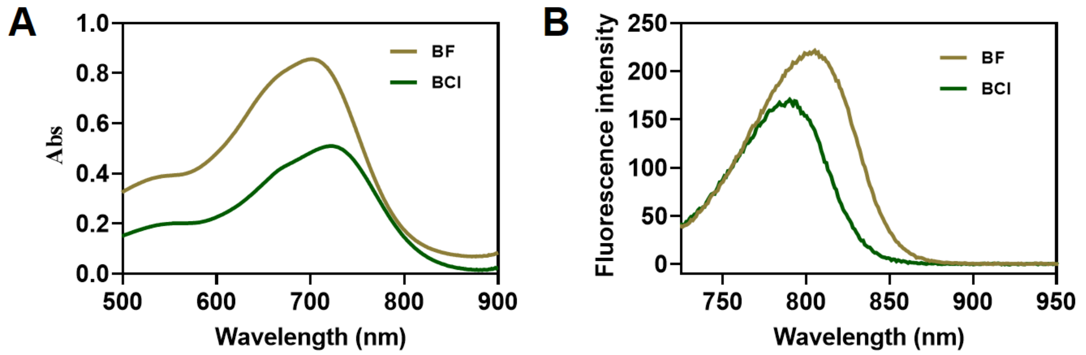

Figure S11. The optical properties characterization of BF and BCl. (A) Ultraviolet-visible absorption spectrum of BF and BCl. (B) Fluorescence spectrum of BF and BCl.

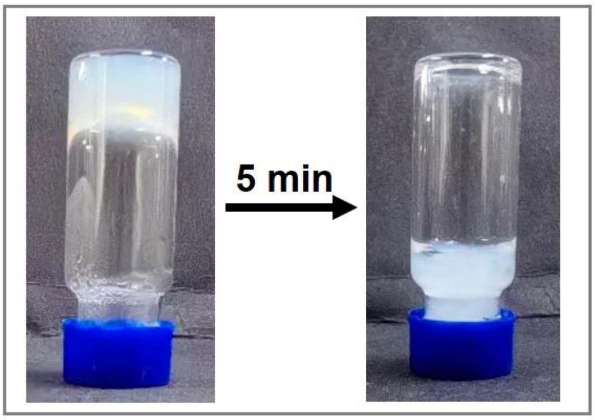

Figure S12. Photograph showing disintegration of ZAg hydrogel after 5 minutes.

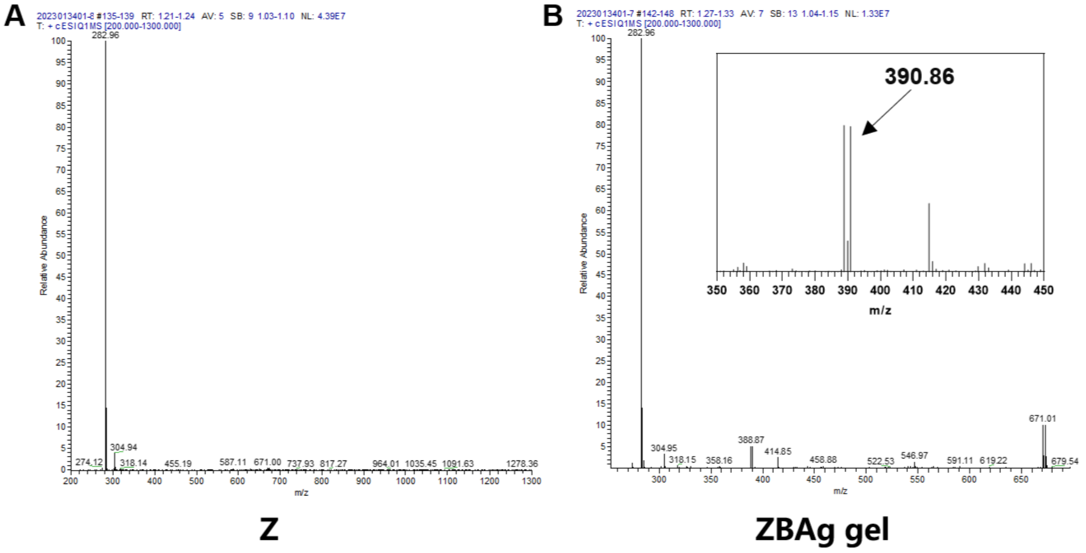

Figure S13. The ESI-MS spectra of Z and ZBAg hydrogel. (A) The ESI-MS spectrum of Z; (B) The ESI-MS spectrum of ZBAg hydrogel.

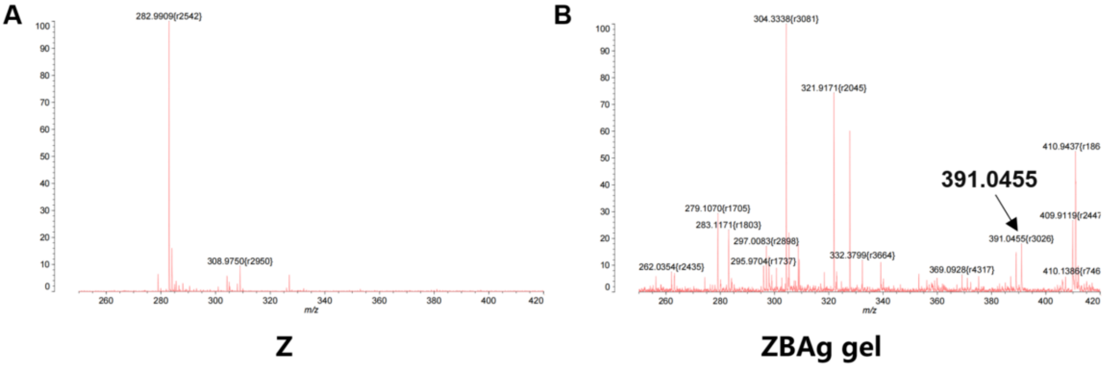

Figure S14. The MALDI-TOF spectra of Z and ZBAg hydrogel. (A) The MALDI-TOF spectrum of Z; (B) The MALDI-TOF spectrum of ZBAg hydrogel.

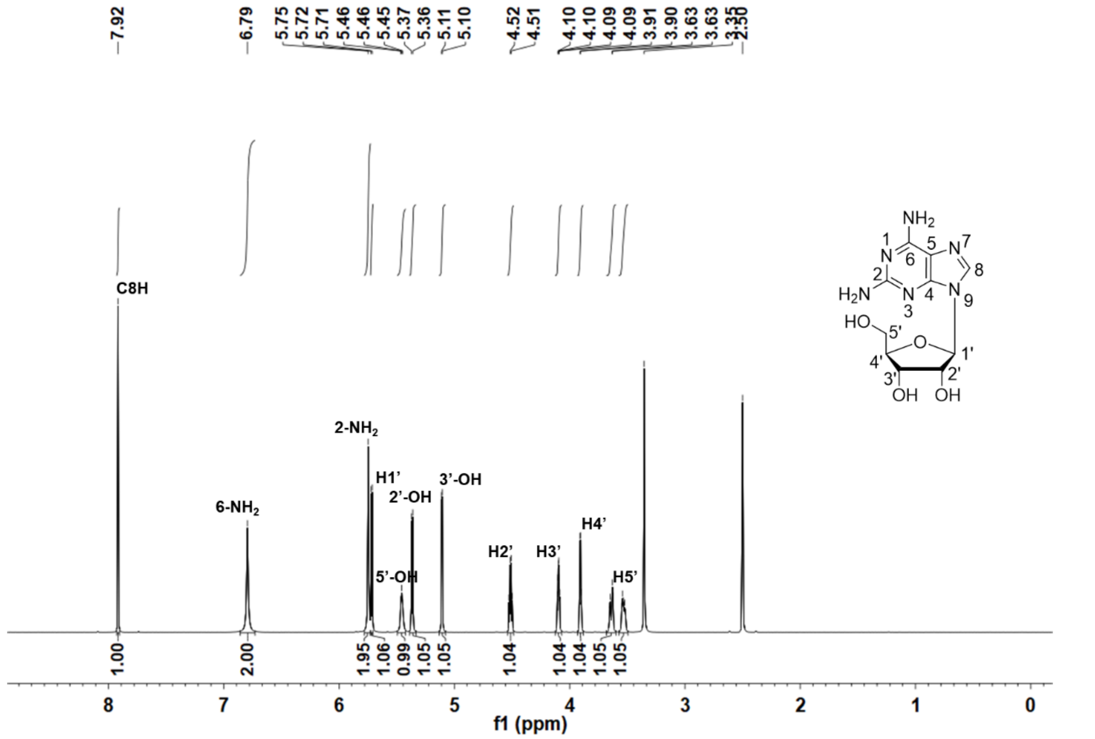

Figure S15. **^{1}H NMR spectrum of Z in DMSO-d_6 and the corresponding ^{1}H NMR signals assignment in Z molecular structure.**

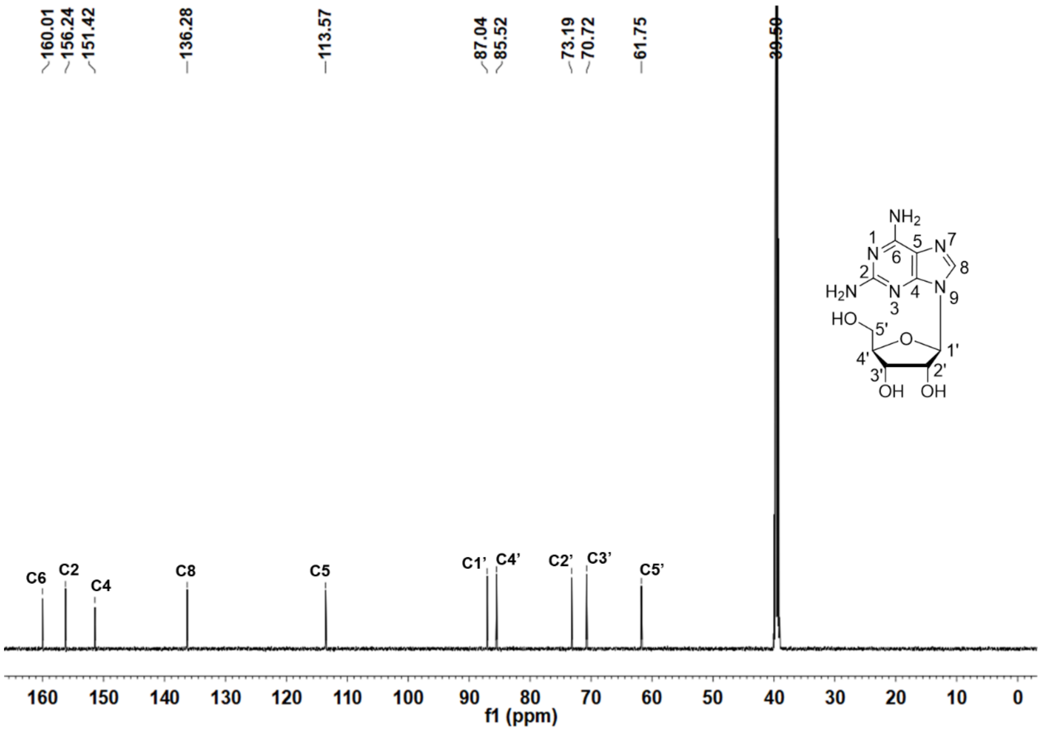

Figure S16. **^{13}C NMR spectrum of Z in DMSO-d_6 and the corresponding ^{13}C NMR signals assignment in Z molecular structure.**

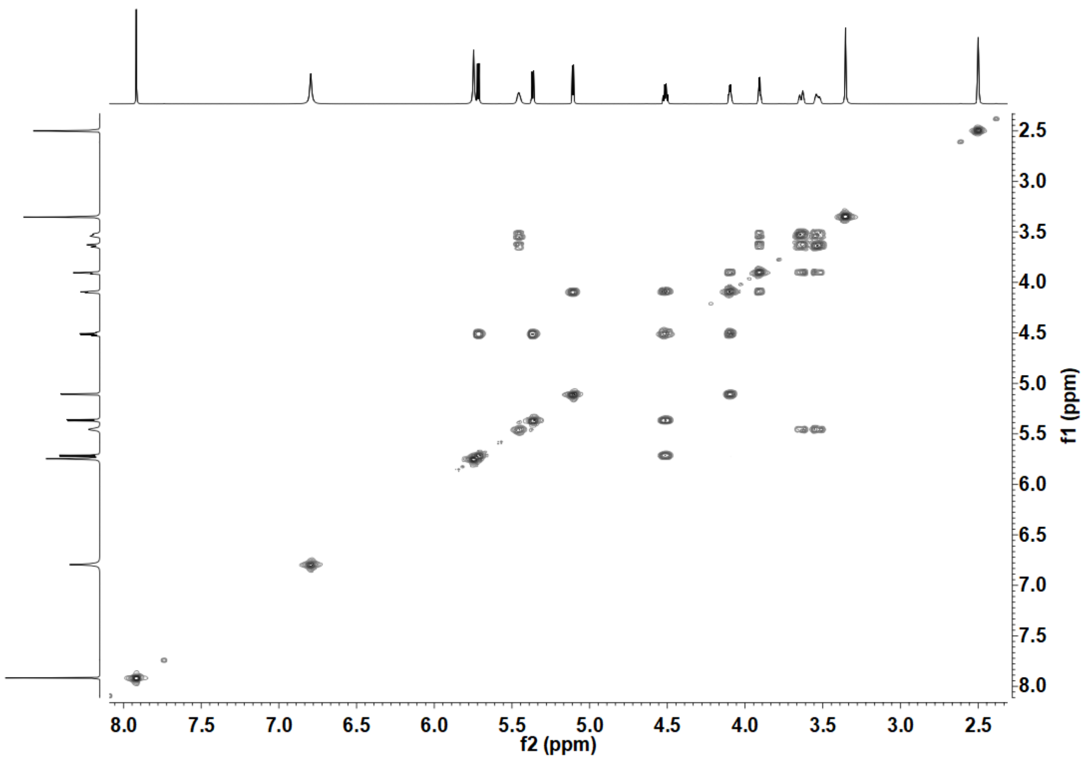

Figure S17. COSY spectrum of Z in DMSO-d_6.

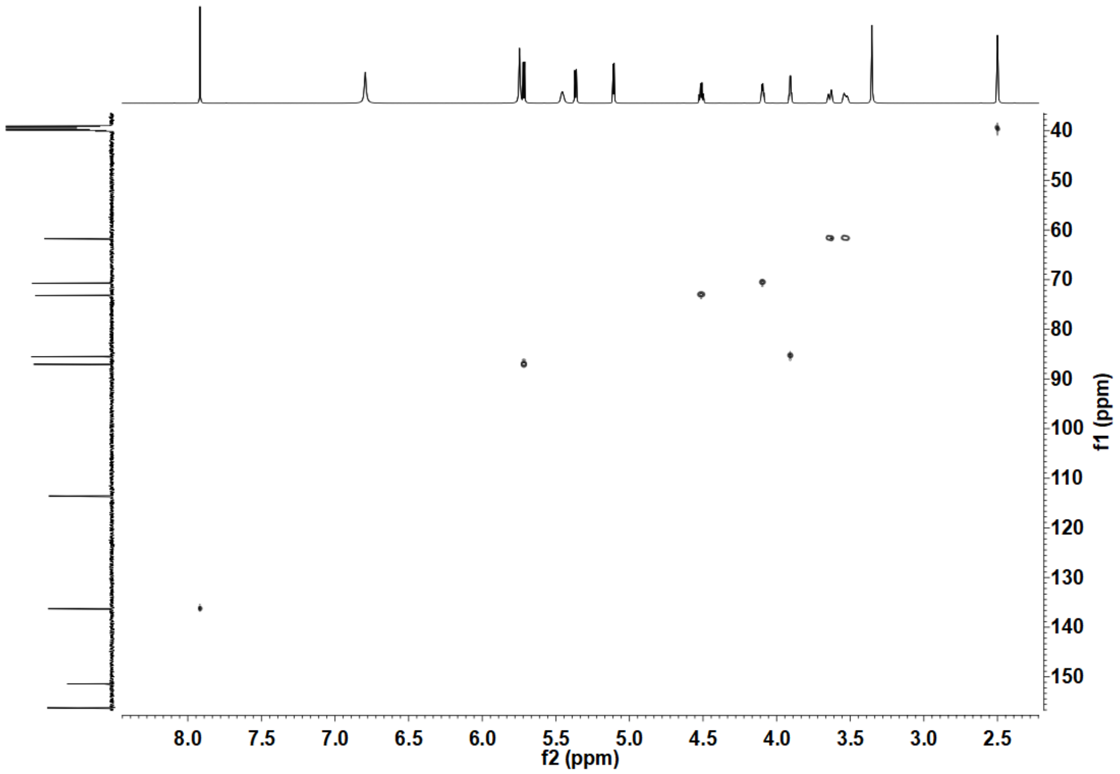

Figure S18. HSQC spectrum of Z in DMSO-d_6.

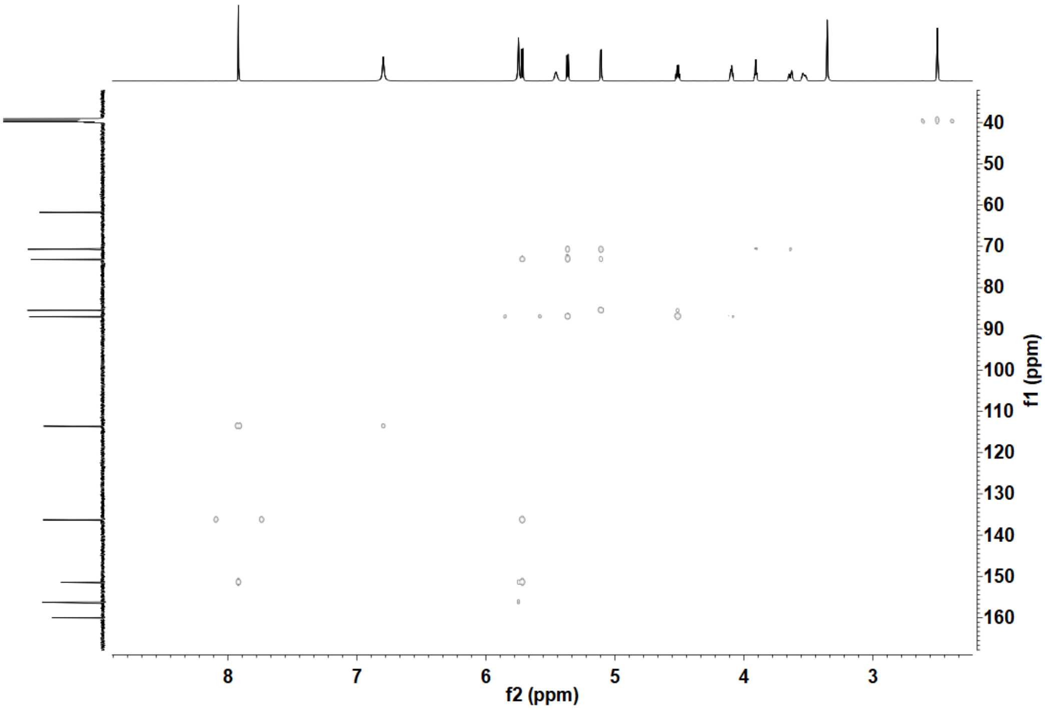

Figure S19. HMBC spectrum of Z in DMSO-d_6.

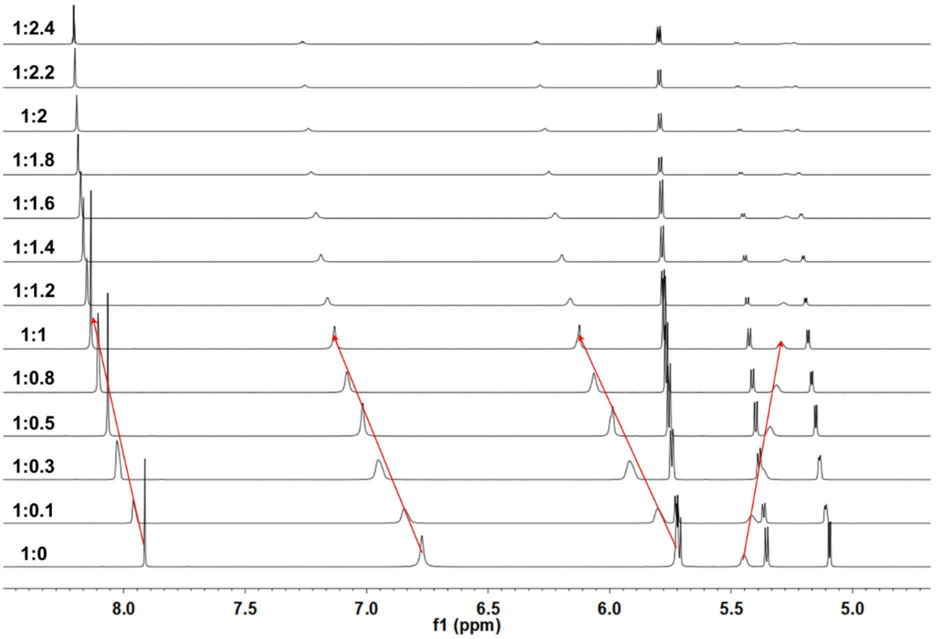

Figure S20. ^{1}**H NMR titration was performed to determine the chemical shift of Z in DMSO-*d*$_6$ at the different ratio of Z/Ag^{+} (from 1:0 to 1:2.4). The red arrows indicate the four signals with the most significant chemical shift changes, corresponding to** ^{1}**H NMR signals of C8H, 6-NH$_2$, 2-NH$_2$ and 5'-OH from left to right.**

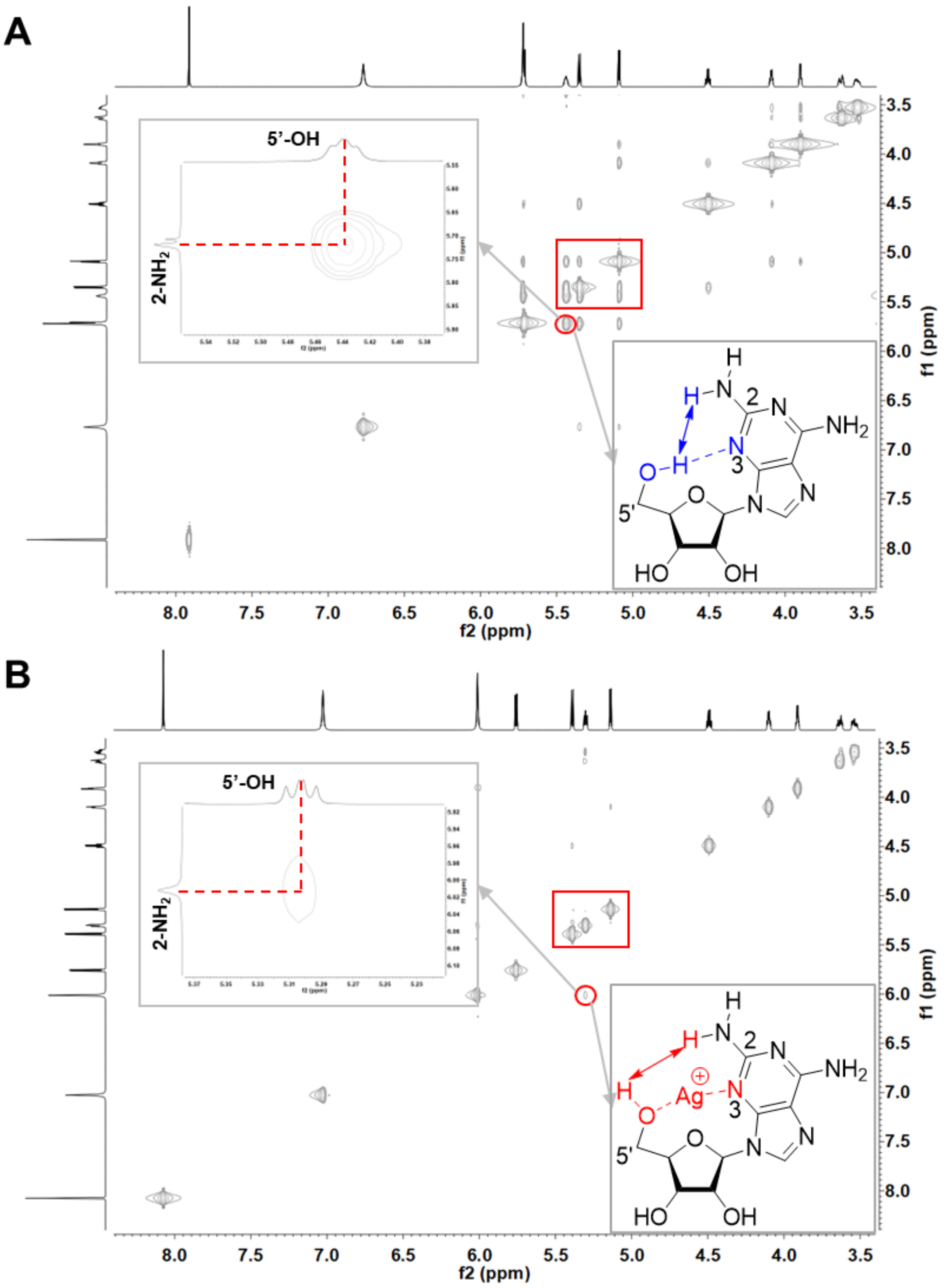

Figure S21. NOESY spectra of Z (A) and ZAg (B) in DMSO-*d*$_6$. The red boxes highlight the NOE signal region between 5'-OH, 2'-OH and 3'-OH. The red circles indicate the NOE signal between 5'-OH and 2-NH$_2$, the insets show a magnified view of the signal and a schematic diagram of the interaction.

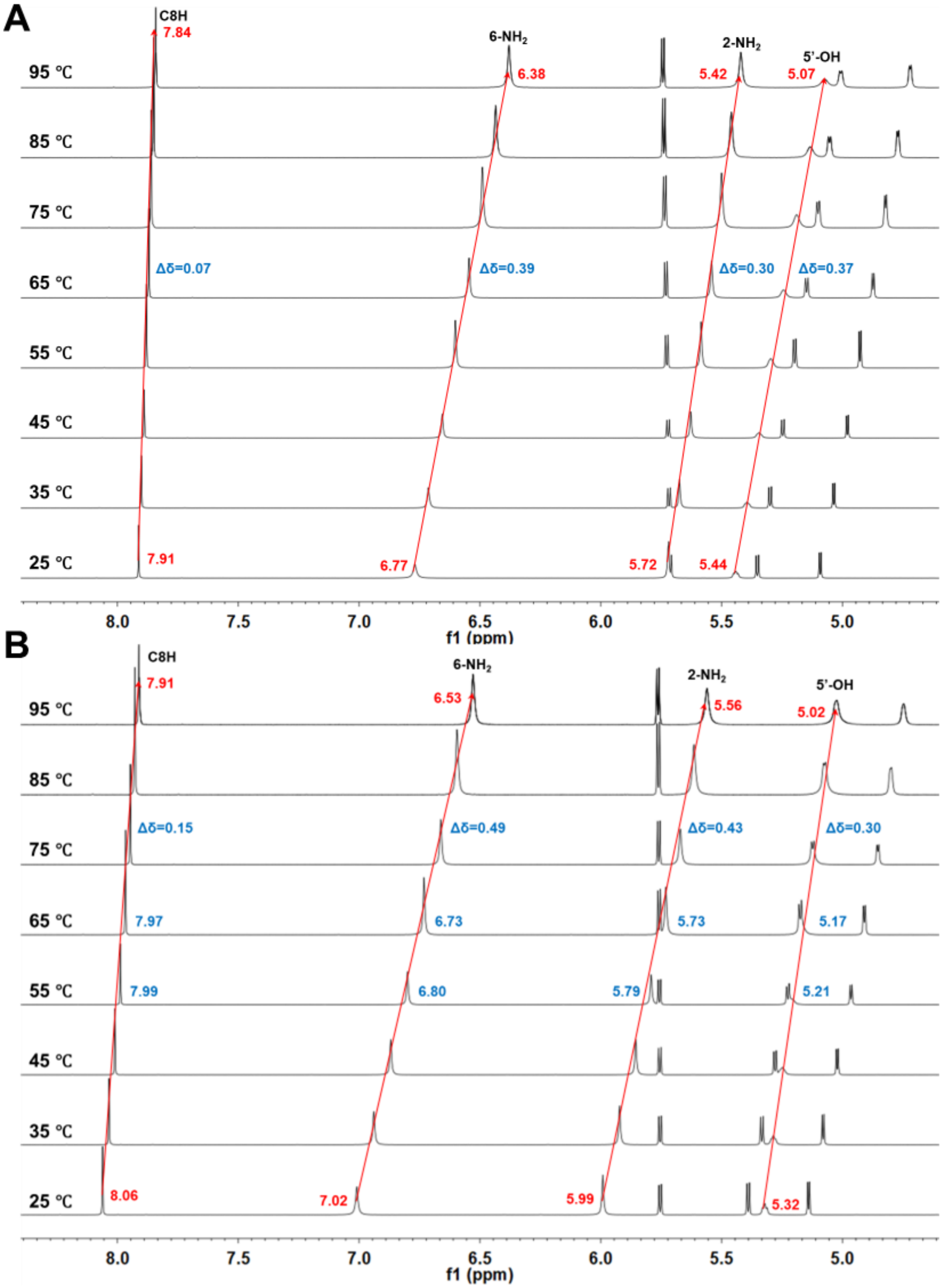

Figure S22. VT ^{1}H NMR spectra of Z (A) and ZAg (B) in DMSO-d_6 were recorded from 25 to 95 °C.

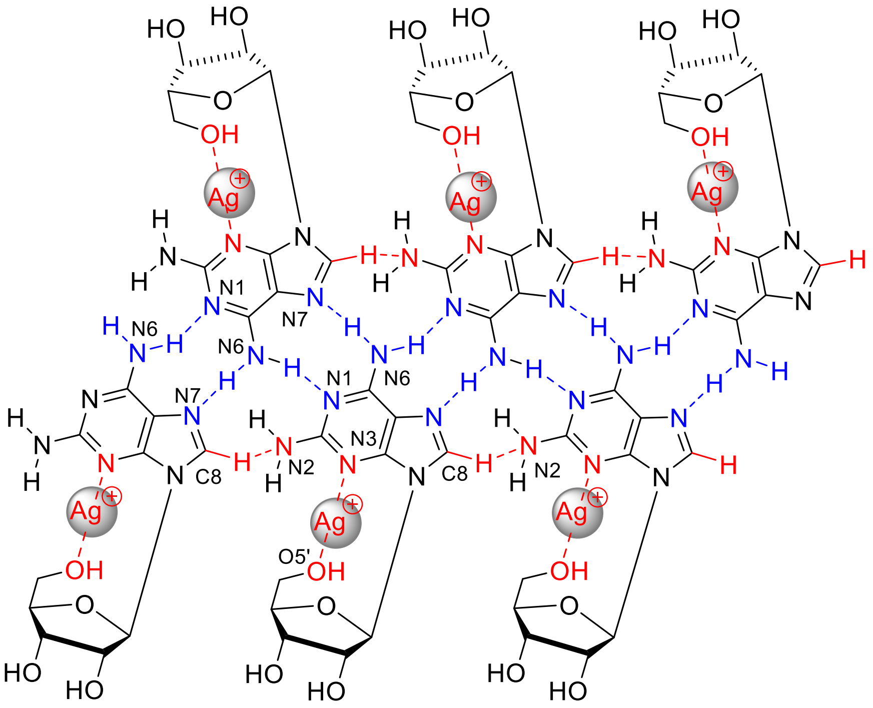

Figure S23. The base pairing and Ag^{+} coordination sites of ZAg.

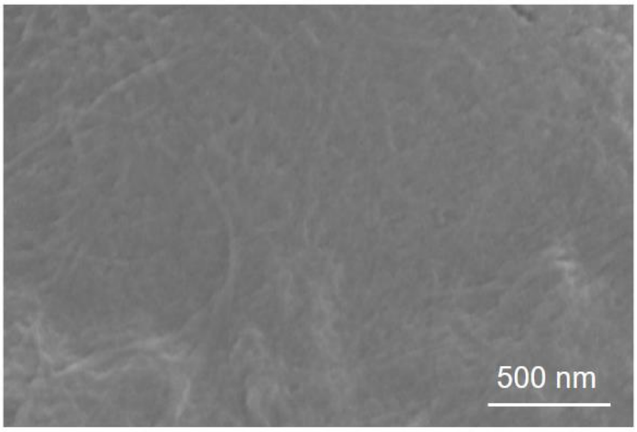

Figure S24. SEM image of ZBAg hydrogel (scale bar: 500 nm).

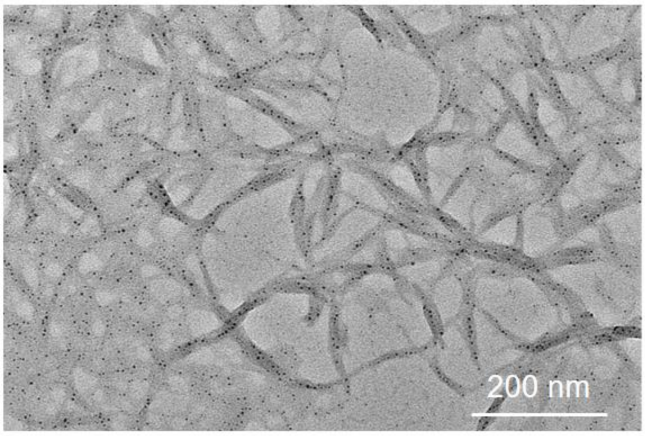

Figure S25. TEM image of ZBAg hydrogel (scale bar: 200 nm).

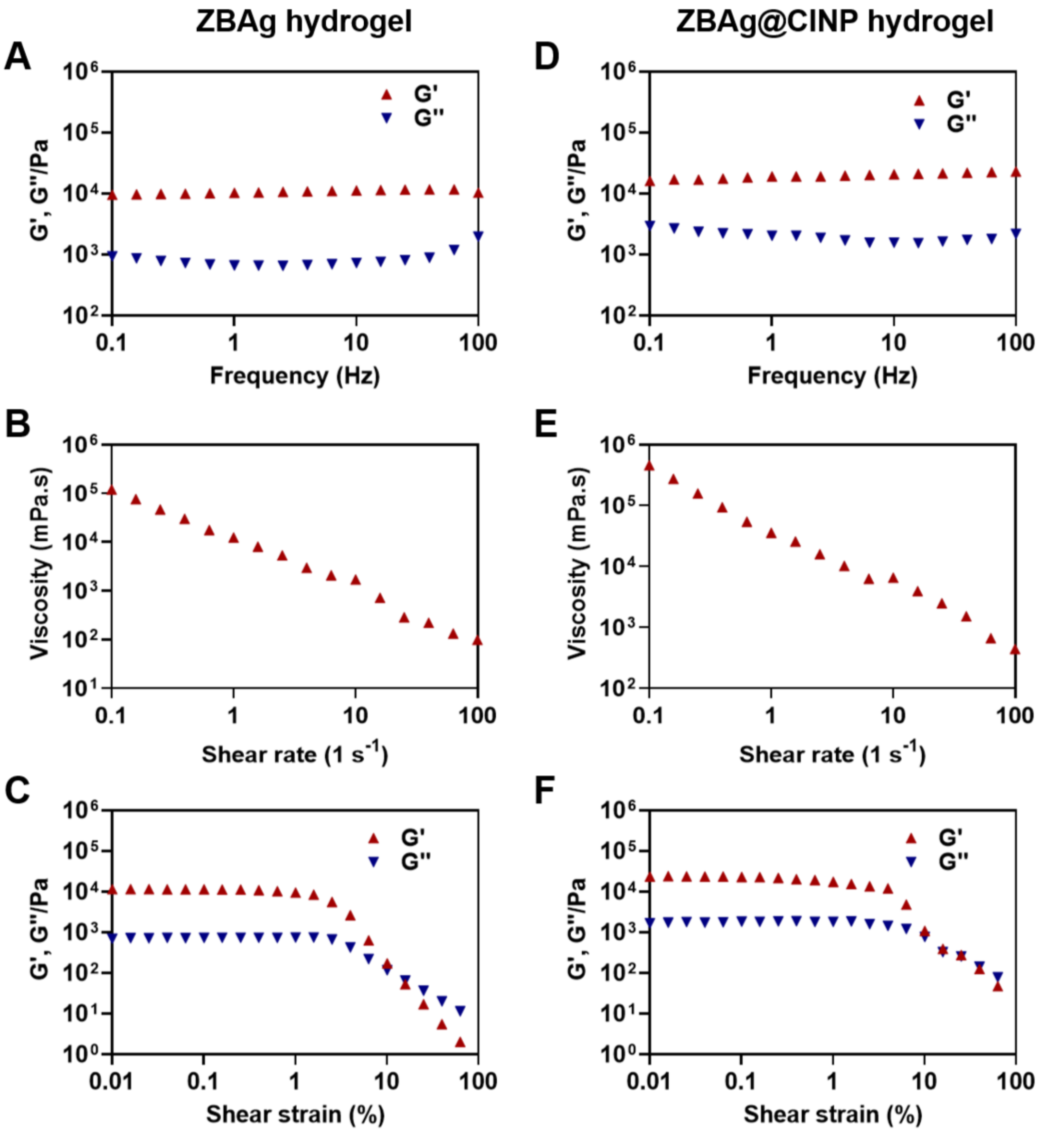

Figure S26. The rheological measurements of ZBAg and ZBAg@ClNP hydrogels. (A) Evolution of G’ and G” as a function of frequency sweep for ZBAg hydrogel. (B) Viscosity test of ZBAg hydrogel. (C) Evolution of G’ and G” as a function of strain for ZBAg hydrogel. (D) Evolution of G’ and G” as a function of frequency sweep for ZBAg@ClNP hydrogel. (E) Viscosity test of ZBAg@ClNP hydrogel. (F) Evolution of G’ and G” as a function of strain for ZBAg@ClNP hydrogel.

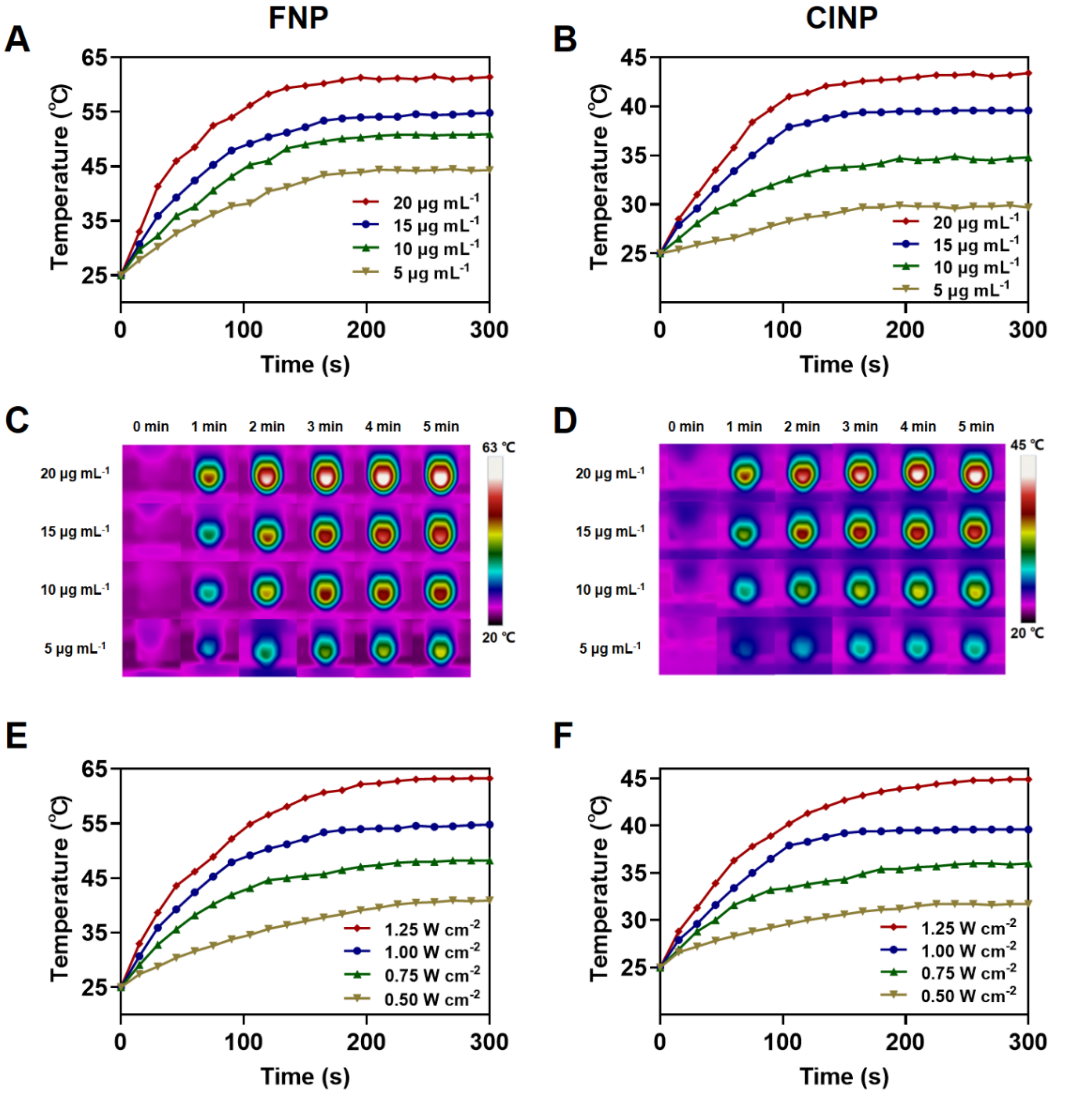

Figure S27. The photothermal performance of FNP and ClNP. (A-B) Temperature change curves of FNP and ClNP at a series of concentrations (5, 10, 15, 20 μg ml^{-1}) under 1.0 W cm^{-2} NIR irradiation. (C-D) Near-infrared imaging of FNP and ClNP at a series of concentrations (5, 10, 15, 20 μg ml^{-1}) under 1.0 W cm^{-2} NIR irradiation. (E-F) Temperature change curves of FNP and ClNP (15 μg ml^{-1}) at different ratios of irradiation intensity (0.50, 0.75, 1.00, 1.25 W cm^{-2}).

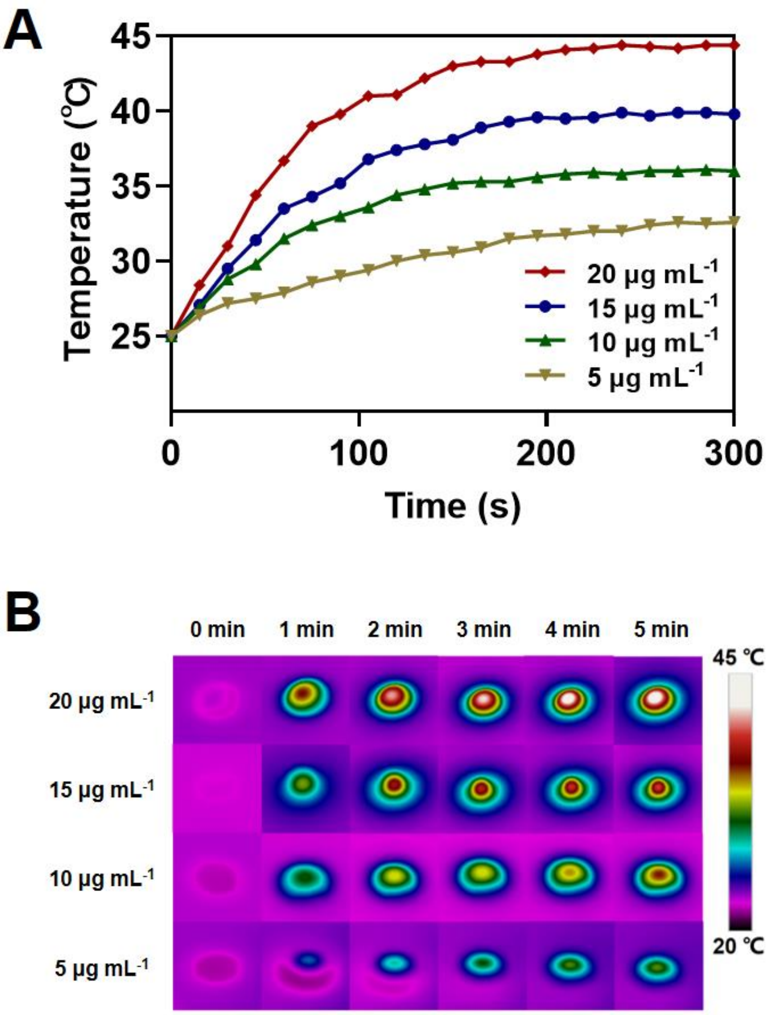

Figure S28. The photothermal performance of ZBAg@ClNP hydrogel. (A) Temperature change curves of ZBAg@ClNP hydrogel at a series of concentrations (5, 10, 15, 20 μg ml^{-1}) under 1.0 W cm^{-2} NIR irradiation. (B) Near-infrared imaging of ZBAg@ClNP hydrogel at a series of concentrations (5, 10, 15, 20 μg ml^{-1}) under 1.0 W cm^{-2} NIR irradiation.

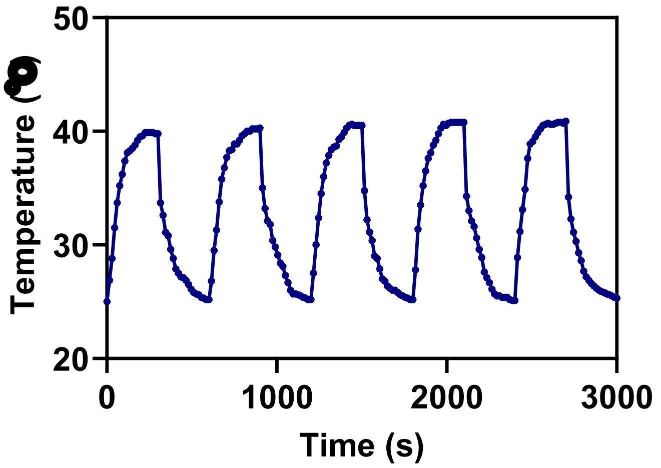

Figure S29. Temperature elevation of ZBAg@ClNP hydrogel (15 μg ml^{-1}, 1 W cm^{-2}) over five cycles of laser irradiation on/off.

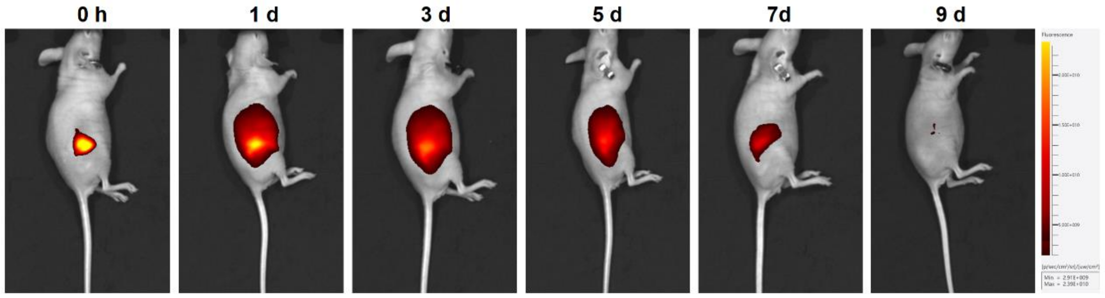

Figure S30. The *in vivo* fluorescence images of mice at 0, 1 ,3, 5, 7 and 9 days after subcutaneous injection of 100 μL ZBAg@FNP hydrogel.

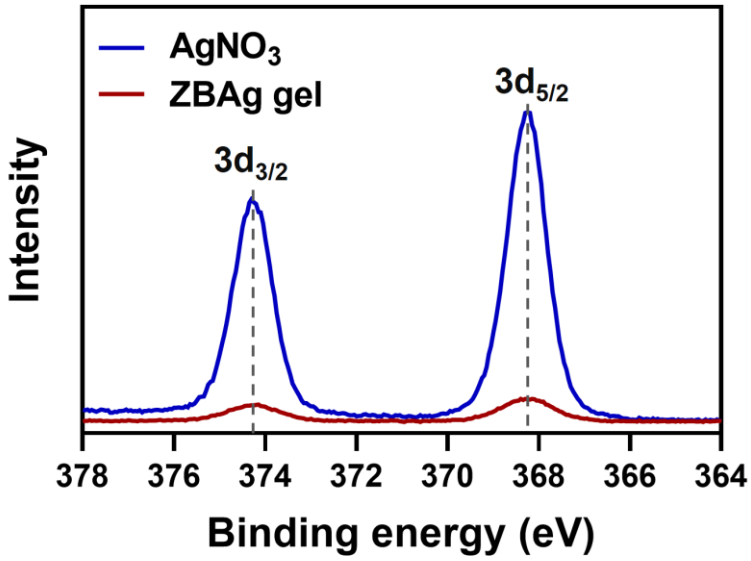

Figure S31. XPS spectra of $AgNO_3$ and ZBAg hydrogel.

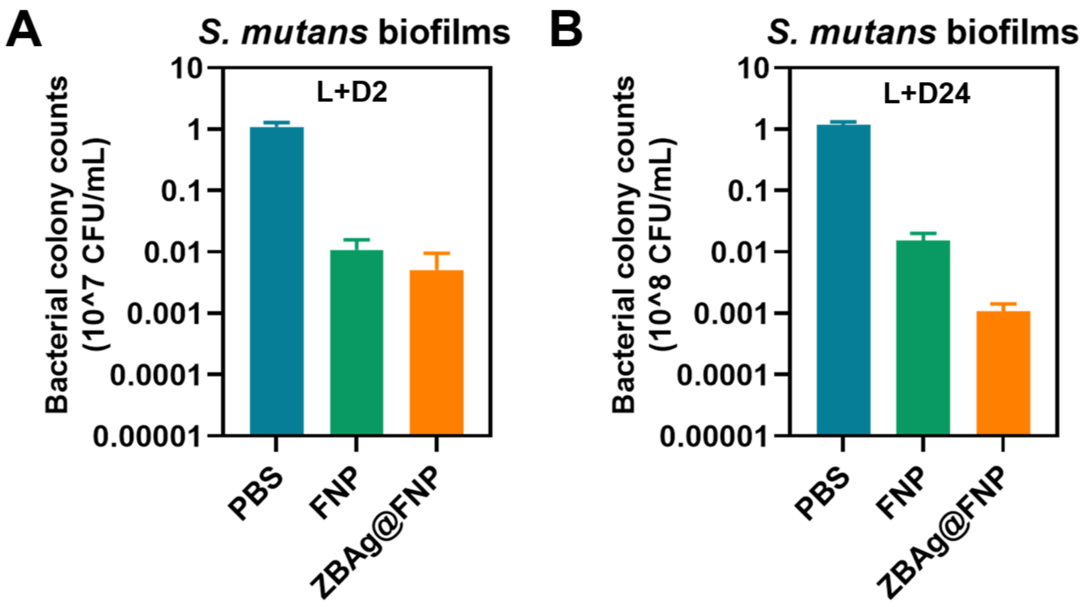

Figure S32. The CFU of *S. mutans* biofilms treated with PBS, FNP, ZBAg@FNP with irradiation at different time point. L+D2: Dark for 2 hours with 5 min light at initiation. L+D24: Dark for 24 hours with 5 min light at initiation.

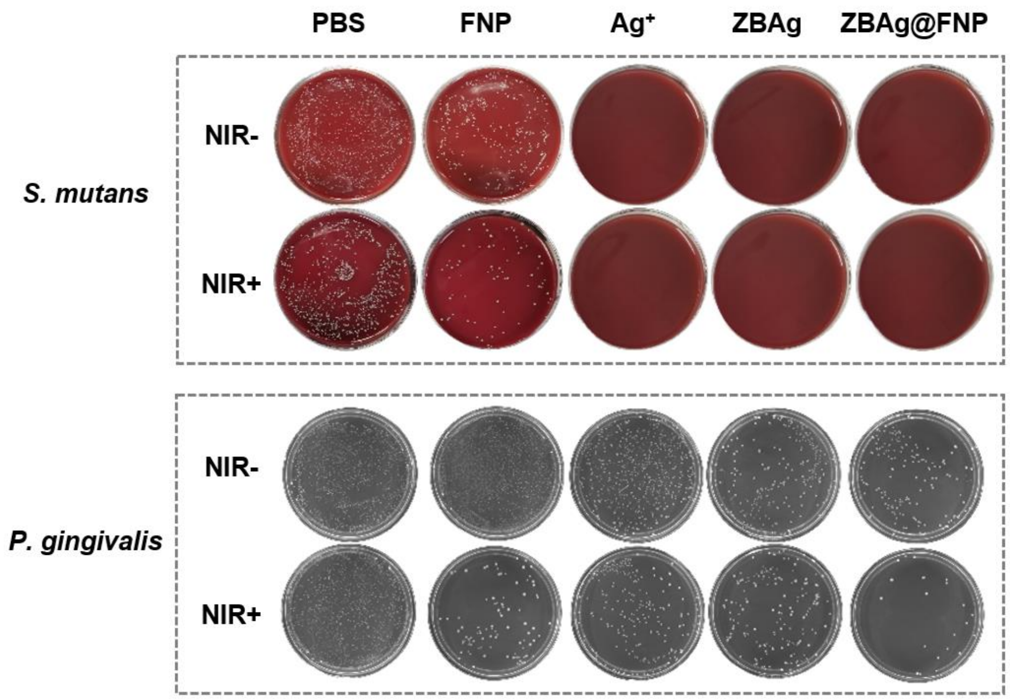

Figure S33. Photographs of bacterial colony in agar plate of *S. mutans* and *P. gingivalis* treated with PBS, FNP, Ag^{+}, ZBAg, and ZBAg@FNP for 24 h.

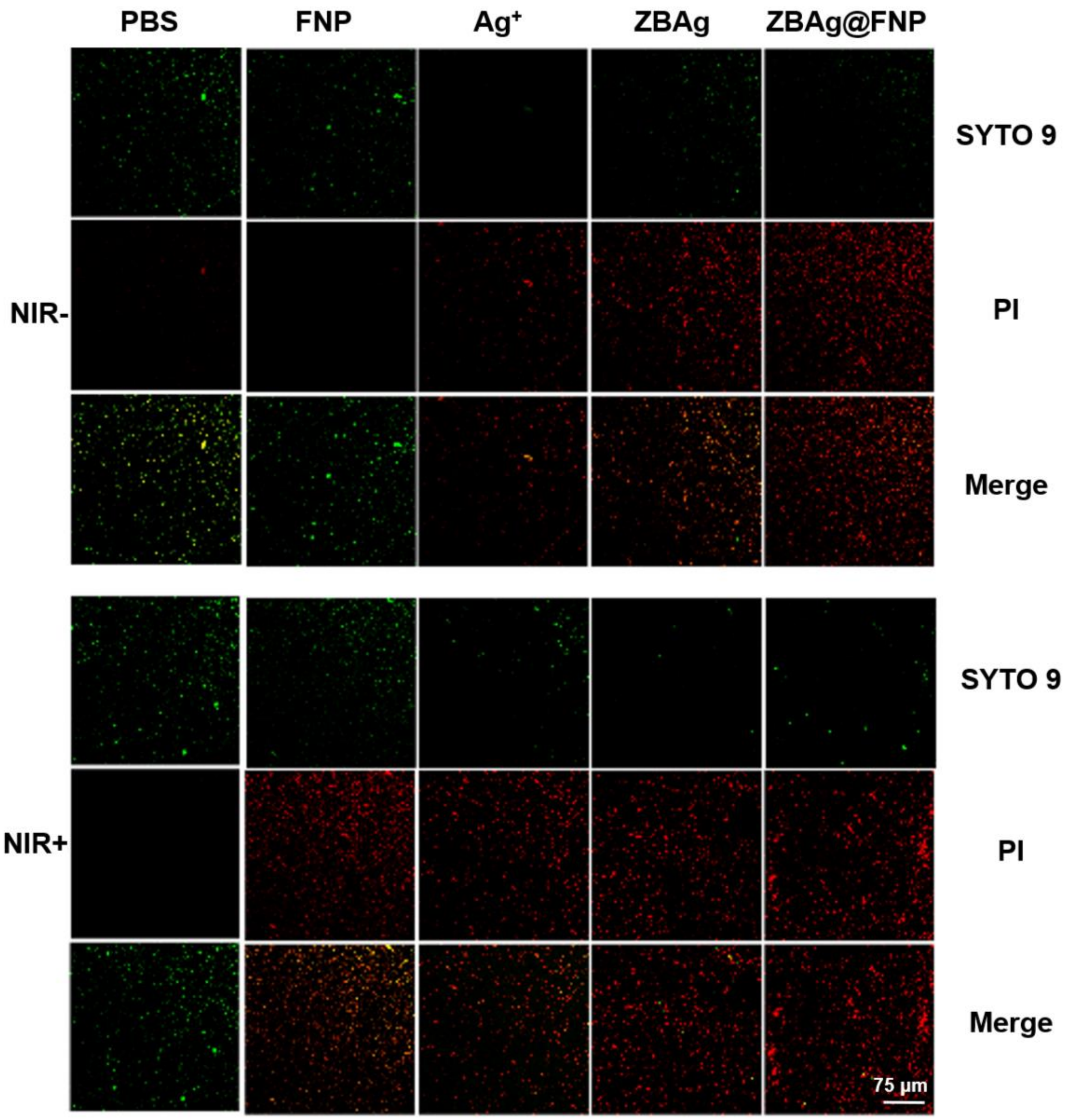

Figure S34. Fluorescence images of *S. mutans* by staining live bacteria (SYTO 9) and dead bacteria (PI) (scale bar: 75 μm).

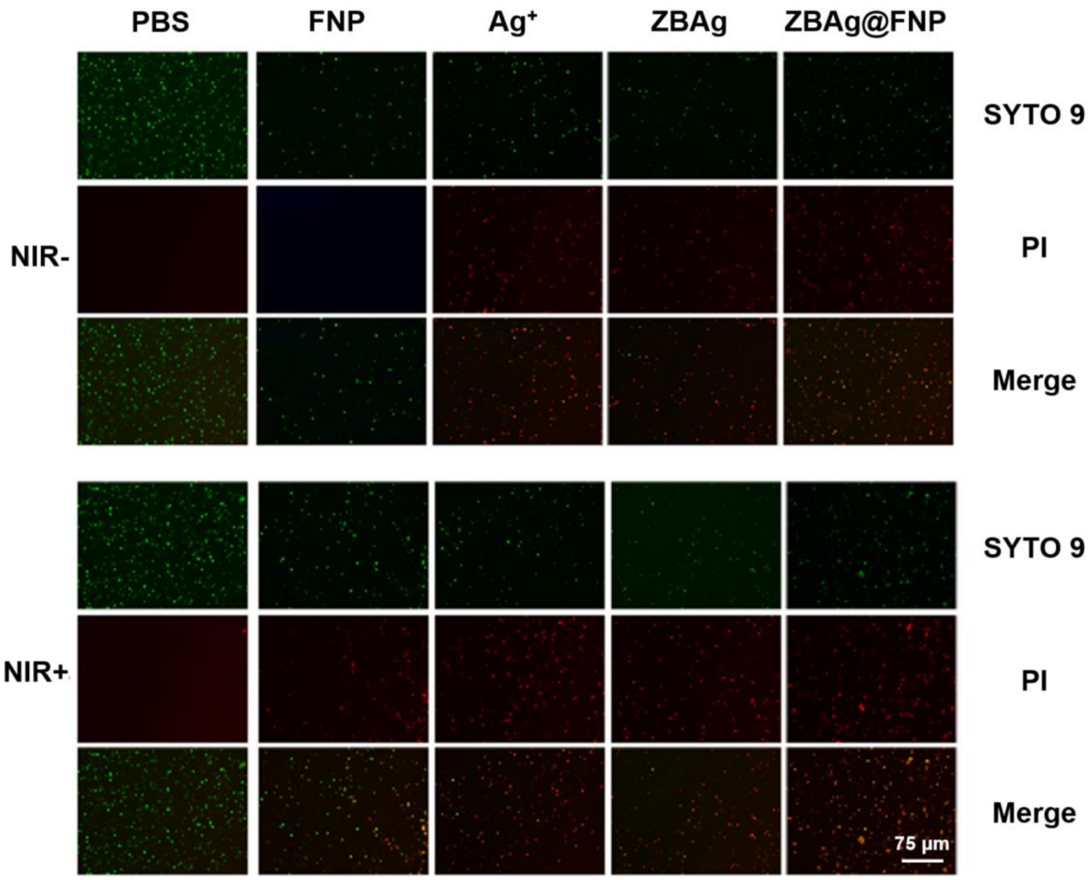

Figure S35. Fluorescence images of *P. gingivalis* by staining live bacteria (SYTO 9) and dead bacteria (PI) (scale bar: 75 μm).

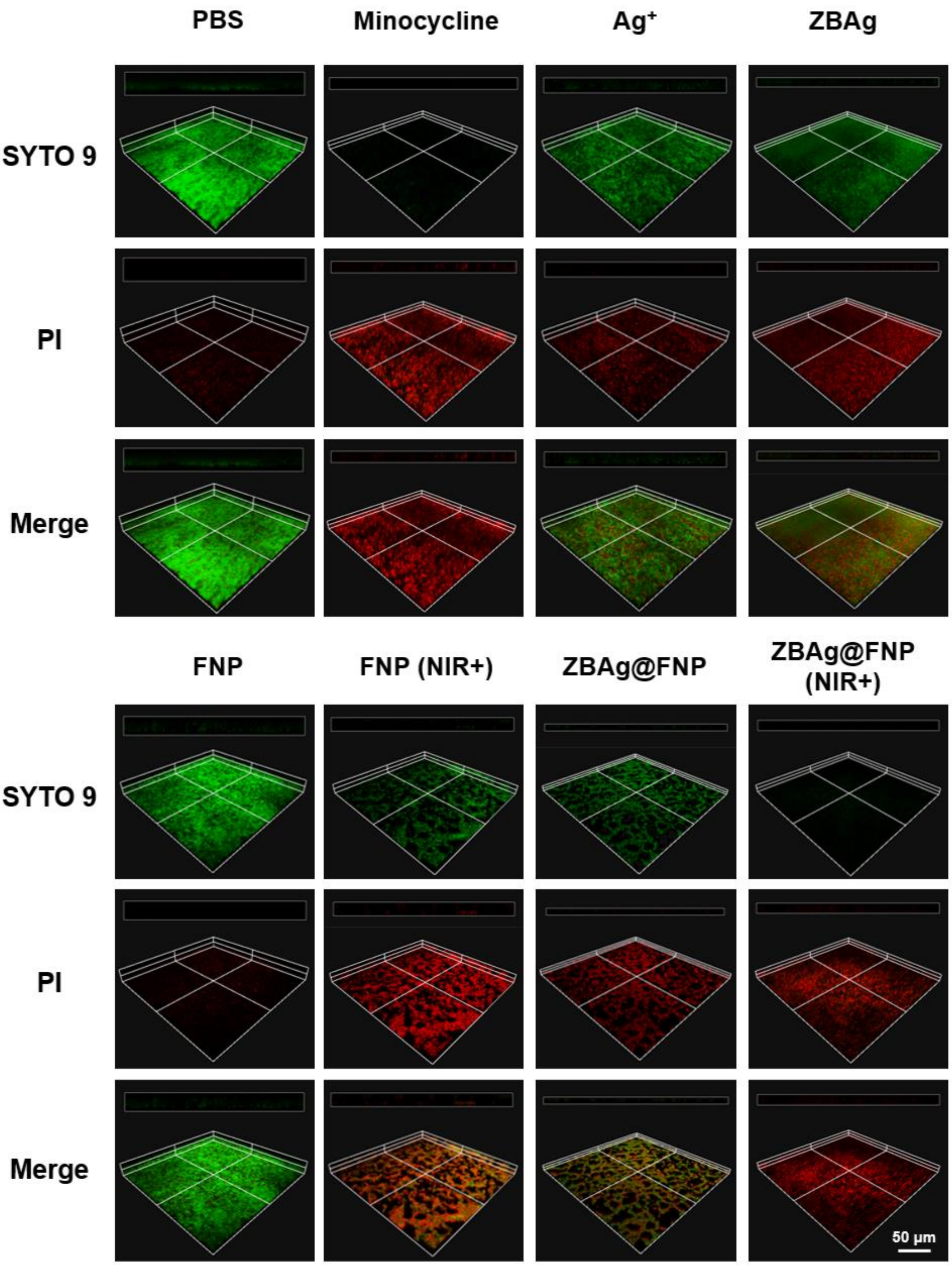

Figure S36. CLSM images of *P. gingivalis* biofilms treated with PBS, minocycline, FNP, Ag^{+}, ZBAg, and ZBAg@FNP for 24 h by staining live bacteria (SYTO 9) and dead bacteria (PI) (scale bar: 50 µm).

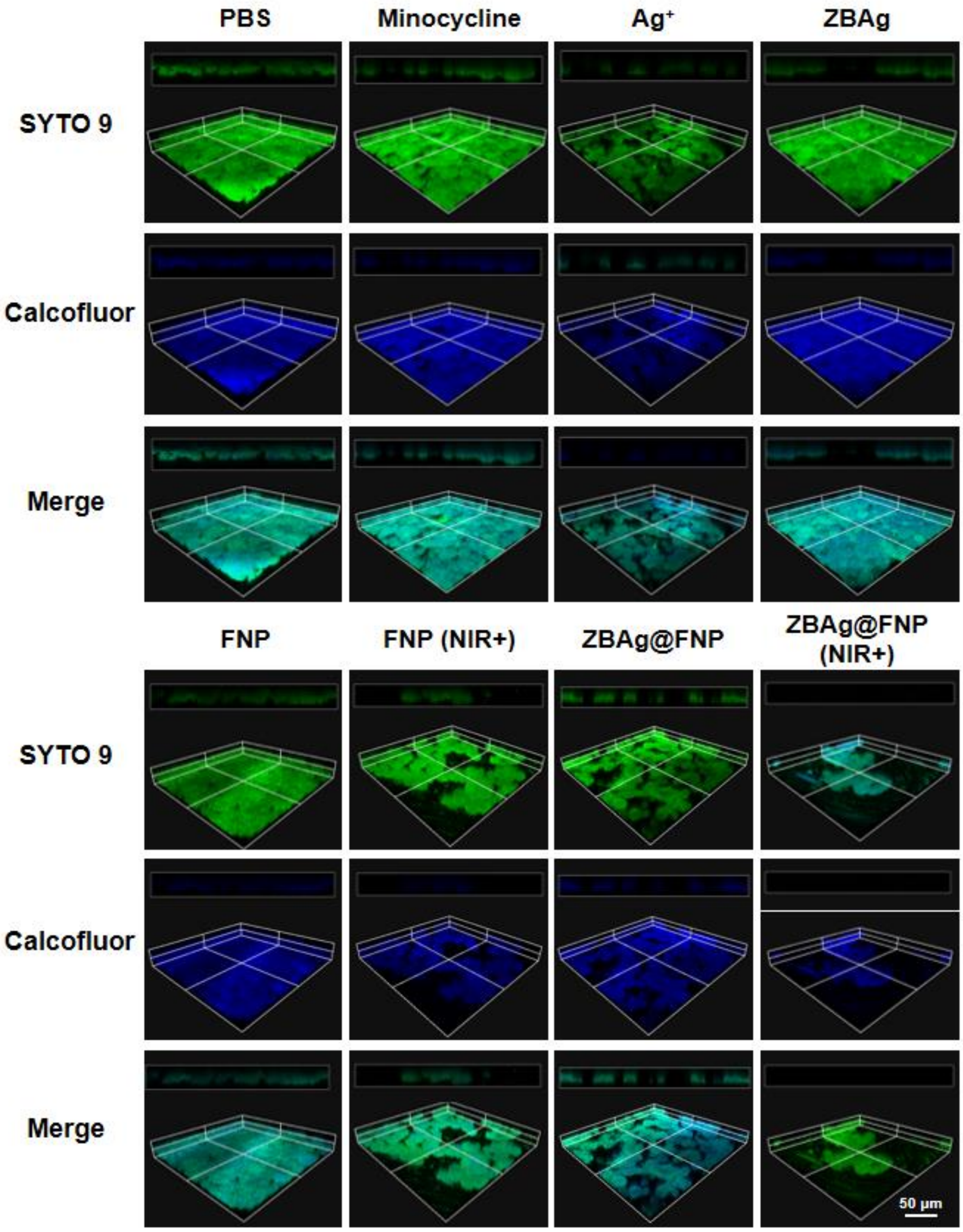

Figure S37. CLSM images of *S. mutans* biofilms treated with PBS, minocycline, FNP, Ag^+, ZBAg, and ZBAg@FNP for 24 h by staining live bacteria (SYTO 9) and polysaccharide (calcofluor) (scale bar: 50 μm).

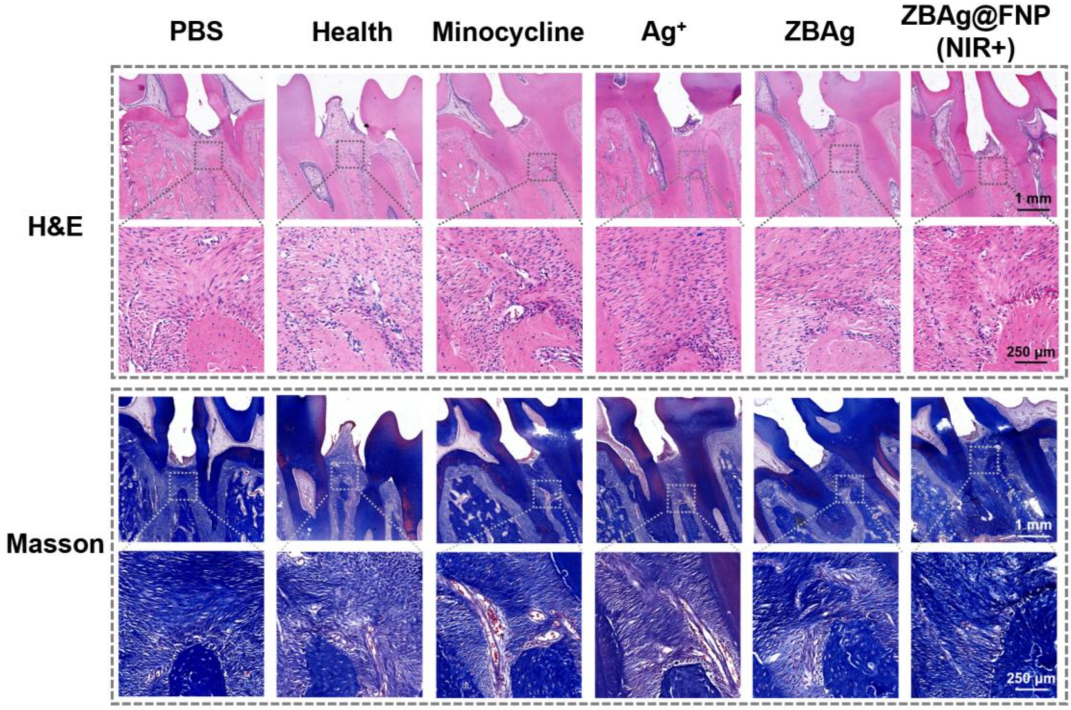

Figure S38. Histopathological characterization of periodontal tissue sections between the first and second molars of maxilla including hematoxylin and eosin (H&E) and Masson images (scale bars: 1 mm, 250 μm).

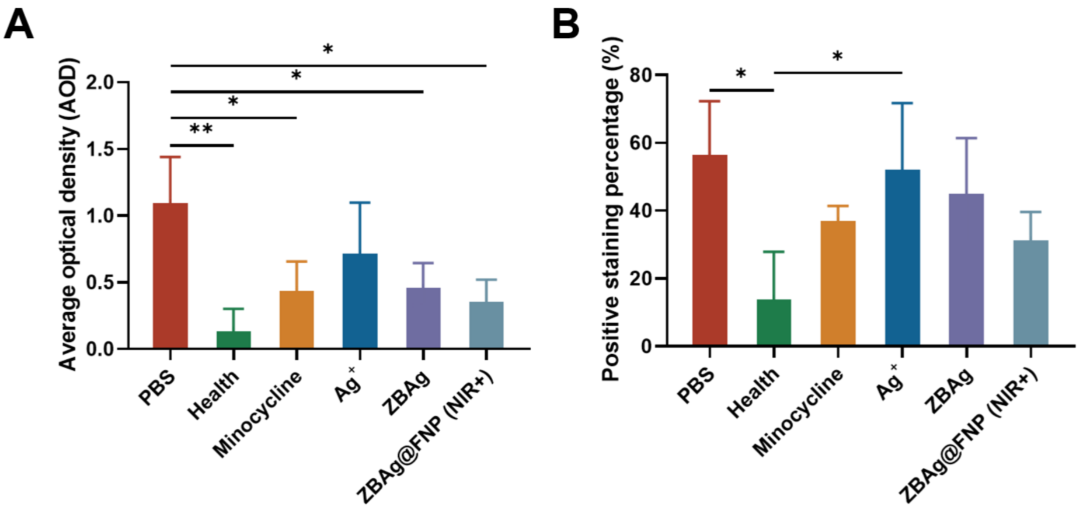

Figure S39. Quantification of the average optical density (AOD) and positive staining percentage in TNF-α immunohistochemical staining.

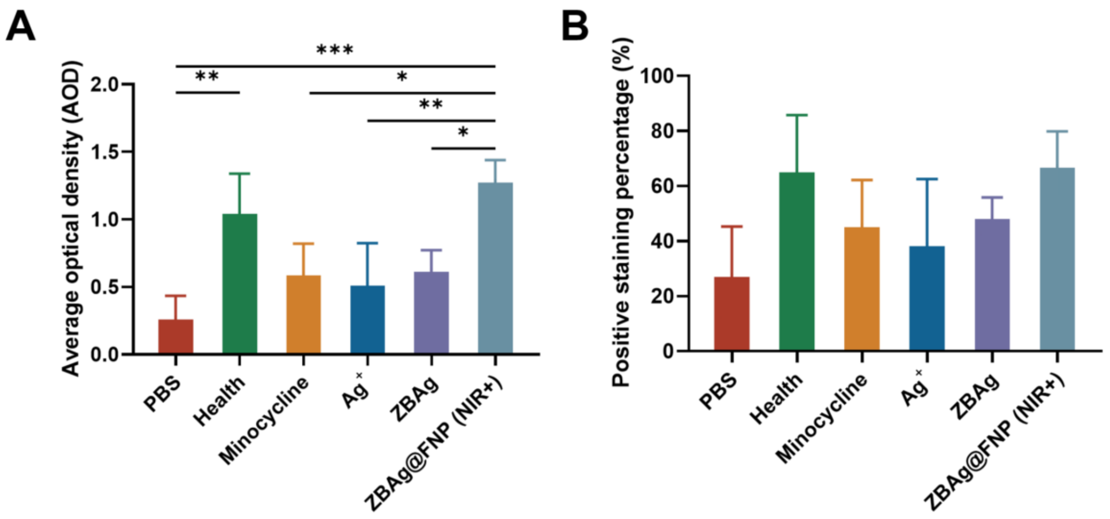

Figure S40. Quantification of the AOD and positive staining percentage in TGF-β immunohistochemical staining.

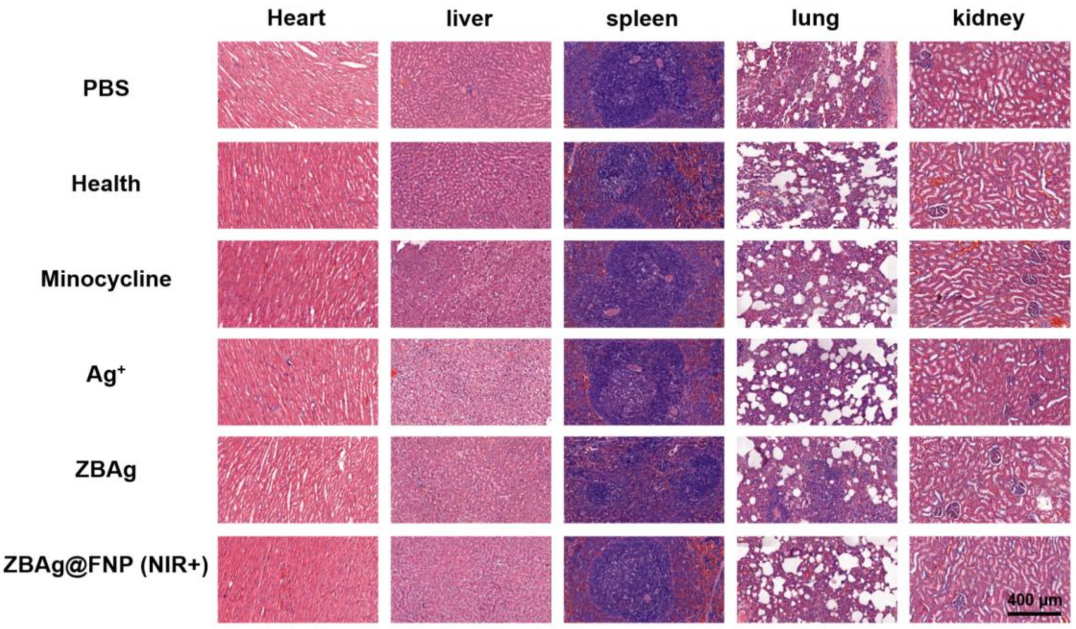

Figure S41. H&E staining images of organs from periodontitis rats subjected to different treatments (scale bar: 400 μm).

Supplementary Discussion

Herein, a comparison of the hydrogel in our manuscript with other literature reports is presented in Table S1 and S2, and a comprehensive summary of the current material is provided from the following aspects:

(1) Facile and green preparation of the ZBAg@FNP hydrogel

The **ZBAg@FNP** hydrogel was synthesized via a simple, one-pot, and environmentally friendly method. In comparison, most previously reported photothermal hydrogels are fabricated through multi-step processes, often involving free radical polymerization, sequential assembly, and amidation reactions. By contrast, the synthesis of the **ZBAg@FNP** hydrogel is significantly more straightforward and efficient.

(2) The multiple advantages and functions of the photothermal agent

The organic small molecule **BF** features a well-defined molecular structure, desirable photophysical properties, and superior biosafety compared to conventional inorganic materials and polymers. Its two-step synthesis is remarkably efficient, in contrast to the often laborious procedures required for NIR-II photothermal molecules. Moreover, the photothermal agent **FNP** exhibits a high photothermal conversion efficiency of 38.74%, surpassing that of commonly used photothermal agents such as Cu nanoparticles (NPs) (26.50%), Mn NPs (22.10%), Fe_3O_4 NPs (34.60%), and certain D–A–D-based zwitterion–liposome hybrid NPs (30.80%). Additionally, **FNP** displays multifunctional photothermal effects: (i) exerting antibacterial action, (ii) disrupting biofilm structures by inactivating proteins and eDNA, and (iii) triggering structural disruption of hydrogel to enable rapid short-term Ag+ release.

(3) Innovative sequential antibiofilm strategy of synergistic antibacterial and antibiofilm action through photothermal effect and released Ag^+

The **ZBAg@FNP** hydrogel's innovation lies in its sequential and multimodal antibacterial strategy. First, the photothermal effect of **ZBAg@FNP** strongly inactivates proteins and eDNA in biofilms and destabilizes the integrity of biofilms,

leading to further permeation by released Ag^+. Moreover, localized thermal energy accelerates the release of Ag^+ from the hydrogel matrix. The released Ag^+ ions penetrate the compromised biofilm structure, disrupt bacterial membranes and increase antibacterial efficacy. The synergistic interplay between photothermal disruption and Ag^+-mediated antibacterial action establishes a robust antibiofilm strategy, offering mechanistic insights for optimizing the design of antibiofilm materials.

Compared to materials that rely solely on single antibacterial agents, such as free Ag^+ ion or photothermal effect alone, the **ZBAg@FNP** hydrogel demonstrates a dramatically synergistic antibacterial and antibiofilm effect through photothermal therapy (PTT) and released Ag^+. The results demonstrate that a 5-log reduction in colony-forming unit (CFU) for *S. mutans*, and 6-log reduction in CFU for *P. gingivalis*. At the same time, a 4-log reduction in CFU for *S. mutans* biofilms, and 5-log reduction in CFU for *P. gingivalis* biofilms. The **ZBAg@FNP** hydrogel is designed to achieve effective antibacterial performance through synergistic PTT and silver ion release, thereby addressing the growing concerns about antibiotic resistance and biofilm-associated infections without relying on conventional antibiotics.

In summary, the photothermal performance of the **ZBAg@FNP** hydrogel fulfills the requirements for the combined antibiofilm strategy demonstrated in this study. It effectively enhances biofilm eradication through the synergistic integration of PTT and the controlled release of Ag^+ ions, offering a promising approach for clinical periodontal applications.

Tabel S1. A comparison of the antibacterial photothermal hydrogel in our manuscript with other literature reports.

Synthesis method of hydrogel	Photothermal agent	Photothermal conversion efficiency	Antibacterial and Antibiofilm Performance	Reference
Step-by-step assembly，free radical polymerization	Noble metal nanoparticles, chitosan-modified palladium nano-cube	43.50%	6-log reduction in colony-forming unit (CFU) for *S. mutans*, and 6-log reduction in CFU for *E. faecalis* and *C. albicans*. 6-log reduction in CFU for *E. faecalis, C. albicans, and polymicrobial biofilms.*	[1]
Dopamine-grafted oxidized chondroitin sulfate, adipic dihydrazide-modified hyaluronic acid, stirred in the dark at RT for 24 h	Metal oxide type, Mn_3O_4 nanoparticles	46.30%	/	[2]
Chitosan gel-forming precursor solution A mixed with 4-armed PEG_{10K}-$(CHO)_4$ gel-forming precursor solution B by ultrasound and crosslinked by the Schiff base	Au-modified nano-hydroxyapatite nanoparticles	/	89.68% clearance rate against Staphylococcus aureus (*S. aureus*) and a 73.03% removal efficiency of Escherichia coli (*E. coli*)	[3]
Amidation reaction	Ceria oxide-molybdenum disulfide nanoparticles with a polydopamine layer	39.40%	*S. aureus* colonies decreased by 55.71 ± 4.10% and *E. coli* colonies decreased by 58.93 ± 0.73%	[4]

Continued Tabel S1. A comparison of the antibacterial photothermal hydrogel in our manuscript with other literature reports.

Synthesis method of hydrogel	Photothermal agent	Photothermal conversion efficiency	Antibacterial and Antibiofilm Performance	Reference
Amidation reaction	Ag_3AuS_2 nanoparticles	39.00%	/	[5]
Chelation self-assembly method and Schiff base reactions	Silver (Ag)-fulvic acid (FA) nanoparticles	52.00%	The anti-*E.coli* antibacterial ratio: 97.16 ± 1.27% and for *S. aureus* 94.40 ± 0.91%.	[6]
Schiff base/ phenylboronate ester double dynamic crosslinking	Polydopamine-reduced graphene oxide compounded glycine-modified fullerene	/	Kill more than 95% of *E. coli* and MRSA within 3 min, and almost all bacteria in 5 min.	[7]
UV light crosslinking	D-type cysteine-modified polydopamine nanoparticles	/	The antibacterial rate of *S. aureus* colonies is 96.4 %, and inhibit biofilm formation.	[8]
By a combination of thermos-gelation and photo-curing strategies.	Black phosphorus nanosheet (BP NS)	/	Antibacterial ratio nearly 100 %	[9]
The dynamic Schiff-based crosslinking as well as the facile photo-triggered secondary crosslinking.	Polydopamine-capped graphene nanosheets (PrGOs)	/	The bacterial survival rates of *S. aureus* and *E. coli* were 6.4 ± 1.3% and 15.1 ± 6.2%, respectively.	[10]

Continued Tabel S1. A comparison of the antibacterial photothermal hydrogel in our manuscript with other literature reports.

Synthesis method of hydrogel	Photothermal agent	Photothermal conversion efficiency	Antibacterial and Antibiofilm Performance	Reference
Copolymerization	Graphene oxide (GO)	/	Significantly inhibited the bacterial growth	[11]
By a chemo-photothermal strategy.	Pd@Au nanoframe		Kill 98% E. coli.	[12]
Via an ultrasonic liquid phase exfoliation method and a bidirectional freeze-casting approach.	Antimonene nanosheets	45.2%	A broad-spectrum antibacterial property against *E. coli* (97.1%) and *S. aureus* (100%).	[13]
Multi-cross-linked	Aggregation-induced emission luminogens (AIEgens)	59.41%	A good bactericidal effect against *S. aureus* (100%) and *E. coli* (98.85%).	[14]
**A facile and green, one-pot method**	**Organic small molecule, FNP**	**38.74%**	**5-log reduction in colony-forming unit (CFU) for *S. mutans*, and 6-log reduction in CFU for *P. gingivalis*.** **4-log reduction in CFU for *S. mutans* biofilms, and 5-log reduction in CFU for *P. gingivalis* biofilms.**	**Our manuscript**

Tabel S2. A comparison of the photothermal agents in our manuscript with other literature reports.

Synthesis method of photothermal agents	Photothermal agents	Photothermal conversion efficiency	illumination conditions	Biocompatibility	Reference
Stiller cross coupling reaction	D-A-D type dual donor fluorescent molecules using benzodithiazole (BBTD), 4H-Cyclopenta[2,1-b:3,4-b'] dithiophene (CPDT) and 3,4-ethylenedioxythiophene (EDOT)	/	980 nm, 6 min, 0.7 W cm^{-2}	Good biological safety, the survival rates of mousederi-ved NIH-3T3 and human-derived LO2 cells are both above 80%	[15]
Mixing aqueous solutions of $K_3[Fe(CN)_6]$ and $ZnCl_2$ in the presence of poly (vinylpyrrolid-one), followed by the addition of HCl and subsequent thermal treatment at 80 °C for 20 hours.	Zinc-doped Prussian blue	39.79%	808 nm, 5 min, 1.2 W cm^{-2}	Have no apparent histological toxicology in *vivo,* excellent biosafety, no systemic toxicity.	[16]

Continued Tabel S2. A comparison of the photothermal agents in our manuscript with other literature reports.

Synthesis method of photothermal agents	Photothermal agents	Photothermal conversion efficiency	illumination conditions	Biocompatibility	Reference
A solution of $Pd(acac)_2$, PVP, and NaBr in a DMPA/water mixture was heated at 100 °C under CO pressure to produce a dark blue product, which was then isolated and purified by precipitation and centrifugation.	Pd nanosheets	52.00%	808 nm, 10 min, 1.0 W cm^{-2}	/	[17]
Self-assembly fine-tuning strategy based on organic-metal coordination interaction	Cu nanoparticles	26.50%	808 nm, 10 min, 0.3 W cm^{-2}	good biocompatibility	[18]
Employing the coordination-assisted polymerization self-assembly strategy	Mn nanoparticles	22.10%	1064 nm, 10 min, 1.2 W cm^{-2}	negligible toxicity	[19]

Continued Tabel S2. A comparison of the photothermal agents in our manuscript with other literature reports.

Synthesis method of photothermal agents	Photothermal agents	Photothermal conversion efficiency	illumination conditions	Biocompatibility	Reference
Modified two-step seedless ex situ growth process,	Au nanorods	40.62%	980 nm, 5 min, 0.1 W cm^{-2}	no noticeable tissue damage or adverse effects to the major organs of animals	[20]
Solvothermal method and emulsion-induced interface anisotropic assembly strategy	MnO_x/PDA nanobombs	34.80%	808 nm, 5 min, 2 W cm^{-2}	No obvious toxic side effects were observed in major organs.	[21]
Noncovalent interaction	flower-like MnO_2 nanoparticles(MnO2/GOx/AIBI)	21.30%	808 nm, 15 min, 1 W cm^{-2}	good biocompatibility in *vivo*	[22]
A thermal decomposition reaction.	protein-corona-coated Fe_3O_4 nanoparticles	34.60%/17.90%	808 nm, 10 min, 1 W cm^{-2} / 650 nm, 10 min, 0.5 W cm^{-2}	no apparent inflamma-tion or injury can be observed in organs.	[23]

Continued Tabel S2. A comparison of the photothermal agents in our manuscript with other literature reports.

Synthesis method of photothermal agents	Photothermal agents	Photothermal conversion efficiency	illumination conditions	Biocompatibility	Reference
A high-temperature thermal decomposition means.	polyethylene glycol modified iridium tungstate ($IrWO_x$-PEG) nanoparticles	27.00%	808 nm, 5 min, 1 W cm^{-2}	no observable system toxicity and good biocompatibility in *vivo*.	[24]
One-pot hydrothermal method	Ultrathin porous nitrogen-doped carbon-coated copper selenide (CuSe)nanoparticles	31.90%	808 nm, 10 min, 3 W cm^{-2}	acceptable biocompatibility	[25]
Modifying a commercial leuco dye (i.e., crystal violet lactone, CVL) via a ring-closing reaction.	spirolactone nanoparticles	36.90%	808 nm, 10 min, 1.5 W cm^{-2}	good biocompatibility and biosafety.	[26]

Continued Tabel S2. A comparison of the photothermal agents in our manuscript with other literature reports.

Synthesis method of photothermal agents	Photothermal agents	Photothermal conversion efficiency	illumination conditions	Biocompatibility	Reference
Pd-catalyzed Suzuki/ Stille coupling reactions	donor–acceptor–donor-based (D–A–D)-based zwitterion-liposome hybrid nanoparticles	30.80%	1064 nm, 6 min, 1.0 W cm^{-2}	excellent biocompatibility.	[27]
/	nanoparticles of donor-acceptor-donor (D-A-D) type conjugated small molecules	35.80%	1064 nm, 8 min, 1.0 W cm^{-2}	no apparent pathological abnormality or damage, biological security.	[28]
Self-assembly of an organometallic Ru(II)-arene complex in aqueous solution.	D-A conjugated Ru(II)-arene complex nanoparticles	24.20%	808 nm, 10 min, 0.5 W cm^{-2}	no obvious abnormalities or organ damages and have no sign of in vivo toxicity.	[29]

Continued Tabel S2. A comparison of the photothermal agents in our manuscript with other literature reports.

Synthesis method of photothermal agents	Photothermal agents	Photothermal conversion efficiency	illumination conditions	Biocompatibility	Reference
Self-assembled in a single-step sonication process. Mature DC membranes were coated the BPBBT dots using the extrusion method.	dendritic cell membranes-coated nanoparticles loaded with NIR-II photothermal agents (DC@BPBBT dots)	30.50%	808 nm, 5 min, 1.0 W cm^{-2}	excellent in *vivo* safety and biocompatibility and no detectable acute side effect.	[30]
Liquid-phase exfoliation strategy through sonicating 10 mm Sn powder in isopropanol by an ultrasound probe and water bath sonication.	Stanine-based nanosheets (SnNSs)	37.90%	808 nm, 10 min, 1.0 W cm^{-2}	No cytotoxicity for normal cells and no acute toxicities for mouse.	[31]

Continued Tabel S2. A comparison of the photothermal agents in our manuscript with other literature reports.

Synthesis method of photothermal agents	Photothermal agents	Photothermal conversion efficiency	illumination conditions	Biocompatibility	Reference
**donor-acceptorcombinations: 1 mg of compound BF/BCl was dissolved in 1 mL THF with 6 mg Pluronic F-127, sonicated until clear, then rapidly injected into 10 mL deionized water under vigorous stirring and stirred at room temperature for 24 hours.**	**FNP nanoparticles**	**38.74%**	680 nm, 5 min, 1.0 W cm^{-2}	**Excellent biodegradability, biocompatibility and biosafety in *vivo***	**Our manuscript**

References

[1] L. Chen, M. Peng, J. Zhou, X. Hu, Y. Piao, H. Li, R. Hu, Y. Li, L. Shi, Y. Liu, Supramolecular Photothermal Cascade Nano-Reactor Enables Photothermal Effect, Cascade Reaction, and In Situ Hydrogelation for Biofilm-Associated Tooth-Extraction Wound Healing, *Advanced Materials* **2023**, *35*, 2301664.

[2] Y. Chen, C. Xue, F. Ni, B. Hu, M. Yan, H. Zhang, Y. Hu, X. Peng, G. Li, Y. Han, H. Liu, Z. Shao, Q. Wang, Y. Wei, Photothermal Hydrogel with $Mn_3 O_4$ Nanoparticles Alleviates Intervertebral Disc Degeneration by Scavenging ROS and Regulating Extracellular Matrix Metabolism, *Advanced Functional Materials* **2025**, e22817.

[3] N. Shao, J. Yao, Y. Qi, Y. Huang, Design of an anti-scald photothermal hydrogel for rapid bacteria removal and hemostasis, *Chemical Engineering Journal* **2023**, *476*, 146642.

[4] Y. Wang, K. Liu, W. Wei, H. Dai, A Multifunctional Hydrogel with Photothermal Antibacterial and AntiOxidant Activity for Smart Monitoring and Promotion of Diabetic Wound Healing, *Advanced Functional Materials* **2024**, *34*, 2402531.

[5] J. Su, S. Lu, S. Jiang, B. Li, B. Liu, Q. Sun, J. Li, F. Wang, Y. Wei, Engineered Protein Photo-Thermal Hydrogels for Outstanding In Situ Tongue Cancer Therapy, *Advanced Materials* **2021**, *33*, 2100619.

[6] R. Xiong, M. Zhou, H. Li, L. Wang, G. Ling, P. Zhang, The Three-Pronged Strategy: A Bilayer Hydrogel Treats Diabetic Chronic Wound through Microalgae Oxygen Therapy, Ag─FA NP Antibacterial, and Synergistic Scavenging of ROS, *Small* **2025**, *21*, 2502205.

[7] G. Pan, M. Li, L. Mu, Y. Huang, Y. Liang, B. Guo, Photothermal/Photodynamic Synergistic Antibacterial Hydrogel Dressing with pH/Glucose Dual Responsive Pirfenidone Release for Diabetic Foot Ulcers, *Advanced Functional Materials* **2025**, *35*, 2416205.

[8] K. Li, E. Xie, C. Liu, J. Hu, Q. Chen, J. Li, H. Wang, Q. Meng, D. Liu, B. Meng, T. Liang, J. Ma, Z. Yuan, L. Wang, W. Shu, H. Mao, F. Han, B. Li, Disguise strategy' to bacteria: A multifunctional hydrogel with bacteria-targeting and photothermal conversion properties for the repair of infectious bone defects, *Bioactive Materials* **2025**, *47*, 343–360.

[9] C. Qin, F. Fei, Y. Wei, Y. Han, D. Hu, Q. Lin, Thermo-sensitive Poloxamer based antibacterial anti-inflammatory and photothermal conductive multifunctional hydrogel as injectable, in situ curable and adjustable intraocular lens, *Bioactive Materials* **2024**, *41*, 30–45.

[10] H. Wei, H. Jing, C. Cheng, Y. Liu, J. Hao, A Biomimetic One-Stone-Two-Birds Hydrogel with Electroconductive, Photothermally Antibacterial and Bioadhesive Properties for Skin Tissue Regeneration and Mechanosensation Restoration, *Advanced Functional Materials* **2025**, *35*, 2417280.

[11] H. Li, J. Sun, S. Qin, Y. Song, Z. Liu, P. Yang, S. Li, C. Liu, C. Shen, Zwitterion

Functionalized Graphene Oxide/Polyacrylamide/Polyacrylic Acid Hydrogels with Photothermal Conversion and Antibacterial Properties for Highly Efficient Uranium Extraction from Seawater, *Adv Funct Materials* **2023**, *33*, 2301773.

[12] F. Wang, S. Deng, C. Song, X. Fu, N. Zhang, Q. Li, Y. Li, J. Zhan, Y. Jiang, M. Liu, M. Chen, Y. Hu, K.-J. Huang, H. Yang, Z. Chen, R. Cai, W. Tan, Pd@Au Nanoframe Hydrogels for Closed-Loop Wound Therapy, *ACS Nano* **2025**, *19*, 15069–15080.

[13] Y. Liu, Y. Xiao, Y. Cao, Z. Guo, F. Li, L. Wang, Construction of Chitosan-Based Hydrogel Incorporated with Antimonene Nanosheets for Rapid Capture and Elimination of Bacteria *Advanced Functional Materials* **2020**, *30*, 2003196.

[14] J. Wu, W. Wang, J. Shen, N. Zhou, Y. Li, B. Z. Tang, M. Zhang, A Thermosensitive Hydrogel with Efficient NIR Photothermal Conversion as Injectable Wound Dressing for Accelerating Skin Wound Healing, *Advanced Functional Materials* **2024**, *34*, 2312374.

[15] C. Wang, F. Wang, W. Zou, Y. Miao, Y. Zhu, M. Cao, B. Yu, H. Cong, Y. Shen, Donor-acceptor-donor small molecules for fluorescence/photoacoustic imaging and integrated photothermal therapy, *Acta Biomaterialia* **2023**, *164*, 588–603.

[16] J. Li, X. Liu, L. Tan, Z. Cui, X. Yang, Y. Liang, Z. Li, S. Zhu, Y. Zheng, K. W. K. Yeung, X. Wang, S. Wu, Zinc-doped Prussian blue enhances photothermal clearance of Staphylococcus aureus and promotes tissue repair in infected wounds, *Nat Commun* **2019**, *10*, 4490.

[17] S. Tang, M. Chen, N. Zheng, Sub-10-nm Pd Nanosheets with Renal Clearance for Efficient Near-Infrared Photothermal Cancer Therapy *Small* **2014**, *10*, 3139–3144.

[18] E. Feng, Y. Liu, S. Lv, D. Liu, S. Huang, Z. Li, F. Song, Fine-Tuning Cu (II)-Induced Self-Assembly of Hydrophilic Cyanine Dyes for Enhanced Tumor Photothermal Therapy, *Advanced Functional Materials* **2022**, *32*, 2209258.

[19] J. Ye, W. Lv, C. Li, S. Liu, X. Yang, J. Zhang, C. Wang, J. Xu, G. Jin, B. Li, Y. Fu, X. Liang, Tumor Response and NIR-II Photonic Thermal Co-Enhanced Catalytic Therapy Based on Single-Atom Manganese Nanozyme, *Advanced Functional Materials* **2022**, *32*, 2206157.

[20] S. Zhang, Z. Li, Q. Wang, Q. Liu, W. Yuan, W. Feng, F. Li, An NIR-II Photothermally Triggered ‘Oxygen Bomb’ for Hypoxic Tumor Programmed Cascade Therapy, *Advanced Materials* **2022**, *34*, 2201978.

[21] S. Liu, T. Zhang, S. Li, Q. Wu, K. Wang, X. Xu, M. Lu, R. Shao, W. Zhao, H. Liu, Biomimetic Nanobomb for Synergistic Therapy with Inhibition of Cancer Stem Cells, *Small* **2023**, *19*, 2206503.

[22] H. Li, K. Yang, L. Hai, Z. Wang, Y. Luo, L. He, W. Yi, J. Li, C. Xu, L. Deng, D. He, Photothermal-triggered release of alkyl radicals and cascade generation of hydroxyl radicals via a versatile hybrid nanocatalyst for hypoxia-irrelevant synergistic antibiofilm therapy, *Chemical Engineering Journal* **2023**, *455*, 140903.

[23] P. Zhang, Y. Qiao, L. Zhu, M. Qin, Q. Li, C. Liu, Y. Xu, X. Zhang, Z. Gan, Y. Hou, Nanoprobe Based on Biominerals in Protein Corona for Dual-Modality MR

Imaging and Therapy of Tumors, *ACS Nano* **2023**, *17*, 184–196.
[24] M. Wang, Y. Liang, F. Liao, M. R. Younis, Y. Zheng, X. Zhao, X. Yu, W. Guo, D.-Y. Zhang, Iridium Tungstate Nanozyme-Mediated Hypoxic Regulation and Anti-inflammation for Duplex Imaging Guided Photothermal Therapy of Metastatic Breast Tumors, *ACS Appl. Mater. Interfaces* **2022**, *14*, 56471–56482.
[25] H. Yan, J. Dong, X. Luan, C. Wang, Z. Song, Q. Chen, J. Ma, X. Du, Ultrathin Porous Nitrogen-Doped Carbon-Coated CuSe Heterostructures for Combination Cancer Therapy of Photothermal Therapy, Photocatalytic Therapy, and Logic-Gated Chemotherapy, *ACS Appl. Mater. Interfaces* **2022**, *14*, 56237–56252.
[26] J. Wang, B. Hao, K. Xue, H. Fu, M. Xiao, Y. Zhang, L. Shi, C. Zhu, A Smart Photothermal Nanosystem with an Intrinsic Temperature-Control Mechanism for Thermostatic Treatment of Bacterial Infections, *Advanced Materials* **2022**, *34*, 2205653.
[27] P. Chen, F. Qu, S. Chen, J. Li, Q. Shen, P. Sun, Q. Fan, Bandgap Modulation and Lipid Intercalation Generates Ultrabright D–A–D-Based Zwitterionic Small-Molecule Nanoagent for Precise NIR-II Excitation Phototheranostic Applications, *Advanced Functional Materials* **2022**, *32*, 2208463.
[28] S. Chen, Y. Pan, K. Chen, P. Chen, Q. Shen, P. Sun, W. Hu, Q. Fan, Increasing Molecular Planarity through Donor/Side-Chain Engineering for Improved NIR-IIa Fluorescence Imaging and NIR-II Photothermal Therapy under 1064 nm, *Angew Chem Int Ed* **2023**, *62*, e202215372.
[29] G. Xu, C. Li, C. Chi, L. Wu, Y. Sun, J. Zhao, X.-H. Xia, S. Gou, A supramolecular photosensitizer derived from an Arene-Ru(II) complex self-assembly for NIR activated photodynamic and photothermal therapy, *Nat Commun* **2022**, *13*, 3064.
[30] X. Yang, T. Yang, Q. Liu, X. Zhang, X. Yu, R. T. K. Kwok, L. Hai, P. Zhang, B. Z. Tang, L. Cai, P. Gong, Biomimetic Aggregation-Induced Emission Nanodots with Hitchhiking Function for T Cell-Mediated Cancer Targeting and NIR-II Fluorescence-Guided Mild-Temperature Photothermal Therapy, *Advanced Functional Materials* **2022**, *32*, 2206346.
[31] W. Chen, C. Liu, X. Ji, J. Joseph, Z. Tang, J. Ouyang, Y. Xiao, N. Kong, N. Joshi, O. C. Farokhzad, W. Tao, T. Xie, Stanene-Based Nanosheets for β-Elemene Delivery and Ultrasound-Mediated Combination Cancer Therapy, *Angew Chem Int Ed* **2021**, *60*, 7155–7164.
